# A syntelog-based pan-genome provides insights into rice domestication and de-domestication

**DOI:** 10.1186/s13059-023-03017-5

**Published:** 2023-08-03

**Authors:** Dongya Wu, Lingjuan Xie, Yanqing Sun, Yujie Huang, Lei Jia, Chenfeng Dong, Enhui Shen, Chu-Yu Ye, Qian Qian, Longjiang Fan

**Affiliations:** 1 Hainan Institute of Zhejiang University, Sanya, 572025 China; 2 Institute of Crop Science, Zhejiang University, Hangzhou, 310058 China; 3 Center for Evolutionary & Organismal Biology, Zhejiang University, Hangzhou, 310058 China; 4 State Key Laboratory of Rice Biology, China National Rice Research Institute, Hangzhou, 310006 China

**Keywords:** Rice pan-genome, Syntelog, Domestication, De-domestication, Introgression

## Abstract

**Background:**

Asian rice is one of the world’s most widely cultivated crops. Large-scale resequencing analyses have been undertaken to explore the domestication and de-domestication genomic history of Asian rice, but the evolution of rice is still under debate.

**Results:**

Here, we construct a syntelog-based rice pan-genome by integrating and merging 74 high-accuracy genomes based on long-read sequencing, encompassing all ecotypes and taxa of *Oryza sativa* and *Oryza rufipogon*. Analyses of syntelog groups illustrate subspecies divergence in gene presence-and-absence and haplotype composition and identify massive genomic regions putatively introgressed from ancient Geng/*japonica* to ancient Xian/*indica* or its wild ancestor, including almost all well-known domestication genes and a 4.5-Mbp centromere-spanning block, supporting a single domestication event in main rice subspecies. Genomic comparisons between weedy and cultivated rice highlight the contribution from wild introgression to the emergence of de-domestication syndromes in weedy rice.

**Conclusions:**

This work highlights the significance of inter-taxa introgression in shaping diversification and divergence in rice evolution and provides an exploratory attempt by utilizing the advantages of pan-genomes in evolutionary studies.

**Supplementary Information:**

The online version contains supplementary material available at 10.1186/s13059-023-03017-5.

## Background

In spite of the indispensable roles of Asian rice (*Oryza sativa*) in food supply and plant biology, its origination and domestication have been under debate for decades, although a considerable amount of archeological and genetic evidence has been proposed to infer the evolutionary trajectory of rice [1–9]. Multiple taxa in classification, recurrent artificial hybridization during breeding, and long-distance dispersal by global trade have hindered our understanding of rice evolution. The most disputed issue is whether domestication events have happened once or independently multiple times, i.e., single domestication with multiple origins [1, 2, 5, 6] versus multiple independent domestication [3, 7–10]. Regardless, many domestication- and improvement-related genes have been identified to underlie domestication syndromes [11, 12].

Recently, rice feralization or de-domestication has drawn increasing interest in agricultural production because the de-domesticated ecotype of rice (weedy rice, *Oryza sativa* ssp. *spontanea*) has severely threatened rice yield and quality. Feralization is an atavistic process in domesticates that has been studied in multiple crops and livestock, as an extension of post-domestication [13]. Previous studies have suggested that weedy rice genomes were mostly derived from local cultivated rice by recurrent and independent de-domestication events and that genetic introgression from wild rice may have contributed to weediness [14–16].

Pan-genomic studies have been conducted in a wide range of crops, including rice [2, 7, 17–21]. By comparing de novo assemblies, large SVs have been discovered, underlying important traits that could not be explained by small-scale variations. How to utilize pan-genomes in evolutionary studies has been little explored. Here, we integrate high-accuracy rice genomes covering all ecotypes and taxa, to revisit the origin, domestication, diversification, and de-domestication of rice by utilizing a syntelog-based pangenome and highlight the significance of genomic introgression in rice domestication and de-domestication.

## Results

### A catalog of high-quality rice genome assemblies

To fully and accurately capture the genomic diversity, we first created a panel of high-quality rice genome assemblies, including newly generated assemblies of 11 weedy and one cultivated accession, using PacBio HiFi mode with an average sequencing depth of 32.4 × . Contigs were anchored on chromosomes using a reference-guided approach, and Hi-C interaction confirmed the order and orientation accuracy for four accessions (Additional file 1: Fig. S1). Average contig N50 of the new assemblies was 19.16 Mbp, and the long-terminal repeat assembly index (LAI) score was 21.71 (Additional file 1: Fig. S2a). Averagely, 97.32% of the core conserved Poales genes (BUSCO) were assembled (Additional file 2: Table S1). The whole-genome synteny against the reference assembly Nipponbare and gapless assembly MH63RS3 [22] suggested high completeness (Additional file 1: Fig. S3).

**Fig. 1:**
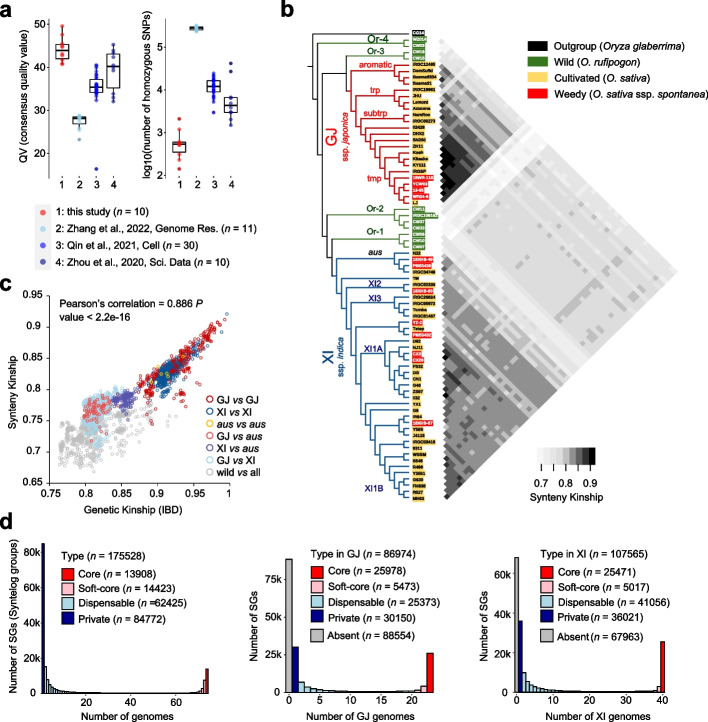
A syntelog-based pangenome of rice. **a** Consensus quality values and number of homozygous SNPs based on self-mapping for genomes in four pan-genome projects. **b** Phylogeny of rice genomes used in this study and their pairwise synteny kinship. **c** The relationship among rice genomes measured by genetic distance (IBD, identity-by-descent) based on SNPs and synteny based on gene orders. **d** Number of syntelog groups (SGs) represented in all 74 rice genomes versus the number of genomes. The subspecies pan-genome compositions for GJ and XI were extracted from the whole rice pan-genome. The numbers of core (present in the genomes of all accessions), soft-core (present in the genomes of > 90% accessions), dispensable (present in the genomes of less than 90% of all but at least two accessions), private (only present in a single genome), and absent SGs (identified by using all populations but absent in sub-population) were counted

We systematically evaluated the base-level accuracy and assembly completeness on rice genomes, including previously released assemblies (Additional file 1: Fig. S4, Additional file 3: Supplemental Note 1). By a combination of several parameters (contig N50, LAI, BUSCO score, consensus quality value (QV), self-calling homozygous single nucleotide polymorphism and sampling size), 74 genomes (including 11 wild, 51 cultivated and 12 weedy rice) were finally used in the following pan-genomic analysis (Additional file 2: Table S1). Phylogeny based on whole-genome SNPs revealed four aromatic (aro), four tropical (trp), two subtropical (subtrp), and 13 temperate (tmp) accessions in GJ subspecies (*n* = 23 in total) and four *aus*, three XI2, seven XI3, ten XI1A, and 16 XI1B accessions in XI subspecies (*n* = 40) (Fig. 1b). Whole-genomic features (e.g., genome size, annotated gene number, and transposon element size) were significantly differentiated between subspecies (Additional file 1: Fig. S2b). The wild population includes two accessions from Or-3 (ancestral group of GJ), four from Or-2, three from Or-1 (ancestral group of XI), and two from Or-4 (basal group of wild rice) (Fig. 1b). The diversity representativeness of the 74 genomes was validated by principal component analysis (PCA) using approximately seven thousand rice accessions (Additional file 1: Fig. S5).

### A syntelog-based pan-genome of rice

Pairwise alignments for each pair of genomes were performed first to identify inter-individual syntelogs (syntenic orthologs) (Additional file 1: Fig. S6). A coalescence-free kinship was built based on pairwise whole-genome synteny (Fig. 1b). Generally, the affinity measured by synteny was linearly correlated with that using identity-by-descent based on SNPs (Pearson’s correlation = 0.886, *P* value < 2.2e − 16) (Fig. 1c). For GJ groups, each group showed a closer relationship to the other GJ groups, and Or-3 was the closest wild-type group. For XI groups, *aus* exhibited differences from the other XI groups in genomic arrangements (Fig. 1b). Although *aus* is sister to other XI groups and nested within Or-1 in the phylogeny (Fig. 1b), the kinship between *aus* and Or-1 measured by synteny was larger than that between *aus* and Or-4, which implied that the wild ancestor of *aus* is distinct from that of the other XI groups (Additional file 1: Fig. S7a). Notably, some XI accessions suggested closer affinity with GJ, suggesting the inter-subspecies hybridization in modern breeding (e.g., Y58S and its offspring J4115) (Fig. 1b, Additional file 1: Fig. S7b). Y58S is an XI-type photothermosensitive genic-male-sterile (PTGMS) line with the characteristics of high-light-efficiency use and disease and stress resistance, and it is widely used for the breeding of two-line hybrid rice varieties. The GJ accession Lemont is one of the parental lines used in the breeding of Y58S (China Rice Data Center, https://ricedata.cn/).

Using a synteny-based gene family clusterer SynPan, approximately 3.10 million genes from the 74 rice genomes were categorized into 175,528 syntelog groups (SGs), consisting of 13,908 core, 14,423 soft-core, 62,425 dispensable, and 84,772 private SGs (Fig. 1d, Additional file 1: Fig. S8a). The SG size is 1.43–1.62 times larger than the number of ortholog groups (OGs) clustered using Markov clustering (MCL) algorithm (Additional file 1: Fig. S9). SynPan performed better than MCL method in distinguishing paralogs against orthologs, especially in the core and soft-core groups (Additional file 1: Fig. S8b). Approximately 34.5%, 34.9%, 27.7%, and 2.8% of genes were assigned to core, soft-core, dispensable, and private SGs, respectively (Additional file 1: Fig. S10a). As essential resistance genes in plant immune response, totally 37,079 nucleotide-binding leucine-rich repeat receptor (NLR) genes were categorized into 998 SGs as rice NLRome (Additional file 3: Supplemental Note 2). Differences in NLR functional types and genomic arrangements were observed between (sub)species (Additional file 1: Fig. S11).

Protein domains could be identified in 81.5% and 68.9% of core and soft-core genes, nearly twice as high as 36.9% and 28.7% for dispensable and private genes, respectively (Additional file 1: Fig. S10b). In total, 1.10% of core genes from 14.1% of core SGs suggested domain gain-and-loss variation, lower than those for soft-core and dispensable types (2.0% of genes in 19.5% of soft-core SGs and 5.0% of genes in 18.9% of dispensable SGs) (Additional file 1: Fig. S10c). For example, two adjacent genes *SaM* and *SaF* encode a ubiquitin-like modifier E3 ligase-like protein and an F-box protein, respectively. Their interactions are responsible for XI-GJ hybrid male sterility [23]. The SGs of *SaM* and *SaF* were both soft-core, present in 68 and 72 genomes, respectively. The domains of all *SaF* syntelogs are completely annotated, while domains of 29 *SaM* syntelogs were not found. Furthermore, the domain presence-and-absence (PAV) variations were typically observed in rice NLRome, which resulted in different NLR types within one single SG (Additional file 1: Fig. S12).

In subspecies level, 49.5% (*n* = 86,974) and 61.3% (*n* = 107,565) of SGs were present in GJ and XI, respectively, indicating the great diversity of wild rice (Fig. 1d). Totally 4662 SGs showed gene PAV biases (frequency difference ≥ 0.6) between subspecies. For example, in the casbene-derived diterpenoid biosynthetic gene cluster DGC7, *CYP71Z21*, *TPS28*, and *CYP71Z2* were almost absent in XI, but fixed in GJ (Additional file 1: Fig. S13), and this may be related to the differential responses of subspecies to biotic stress [24]. *RePRP1* and *RePRP2* are functionally redundant suppressors of root cell expansion [25]. Both *RePRP1* and *RePRP2* were present in GJ, while only *RePRP2* was found in most XI and wild accessions, which implied that the copies of RePRP genes may underlie the differential root development in different subspecies (Additional file 1: Fig. S13).

### Rice evolution based on the mosaic genomic map of syntelog haplotypes

**Fig. 2:**
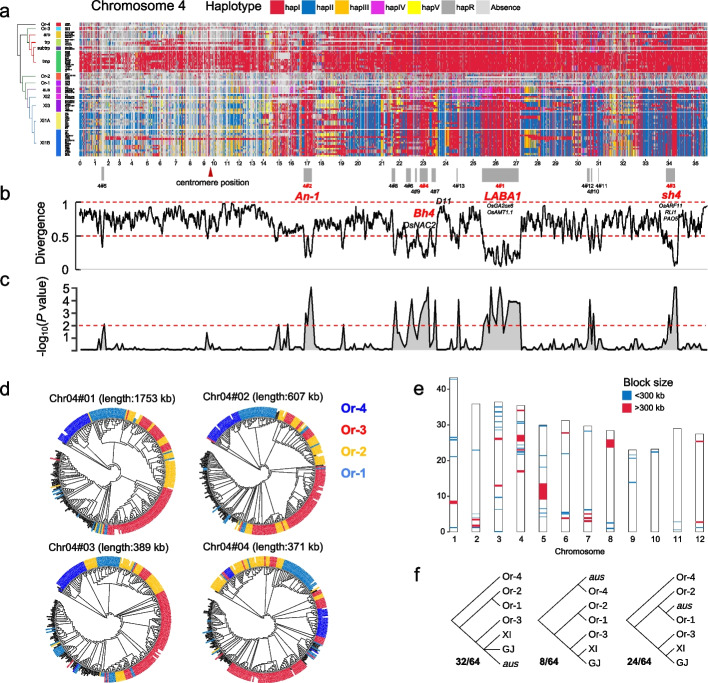
Syntelog-based ancestral haplotypes suggest widespread genomic introgression in rice evolution. **a** Ancestral haplotype landscape of SGs on chromosome 4. An SNP-based phylogeny of rice groups is illustrated on the left. For each SG, seven blocks with different colors represent different haplotypes of predicted protein sequences. Candidate introgression regions are numbered by genomic length and marked by gray blocks. The centromere position information of chromosome 4 is obtained from the Rice Genome Annotation Project and indicated by a red triangle. Functionally important genes are annotated within each block, and domestication genes are highlighted in red. **b** Haplotype divergence between XI and GJ for all SGs on chromosome 4. **c** Significance test on the non-random distribution of clustered SGs in introgression blocks by sampling 100,000 replicates. The horizontal red dashed line represents a *P* value of 0.01. **d** Phylogeny analysis of four large introgression blocks (length greater than 300 Kbp) indicates a single origin of domestication alleles from the Or-3 wild group. Four wild groups are highlighted in different colors. **e** Introgression blocks from proto-GJ to XI based on the evidence of haplotype similarity, phylogeny and ABBA-BABA tests. **f** Complex genomic contributions from wild groups to the emergence of *aus* group revealed by the phylogenetic trees in 64 introgression blocks

Using a semi-supervised approach (Additional file 1: Fig. S14), haplotypes were reassigned according to their abundance in different groups for each SG, labeled as hapI to hapV, hapR (all other rare haplotypes), and absence (represented by red, blue, orange, yellow, green, dark gray, and light gray in Fig. 2a and Additional file 1: Fig. S15) to represent the ancestral sources of rice haplotypes. Whole-genome haplotype maps visually reflected the shared haploblocks at the individual and group levels. For example, mosaic genomes of SN265, DHX2, and 02428 from GJ tmp group and Y58S, J4115, and FH838 from XI1B group suggested massive introgression from the other subspecies (Fig. 2a, Additional file 1: Fig. S15).

We calculated haplotype divergence (HDG) between GJ and XI (Additional file 1: Fig. S14b). The average HDG for all SGs was 0.667, and 46.0% of SGs suggested high haplotype divergence (HDG > 0.8), including 14 of 21 well-known improvement genes [11], implying independent selection for yield- and flowering-related genes in the improvement of XI and GJ (Additional file 2: Table S2). Notably, 720 SGs showed weak divergence (HDG < 0.2), including domestication genes *Prog1* (HDG = 0.025), *GAD1* (0.110), and *sh4* (0.155). The SGs with HDG < 0.5 were merged into 73 blocks, with a total length of 23.38 Mbp (6.24% of Nipponbare assembly) (Fig. 2b, Additional file 1: Fig. S15). Introgression and incomplete lineage sorting (ILS) would both result in haplotype similarity and low divergence between sequences from two lineages. To distinguish introgression from ILS, which is likely to be randomly distributed along chromosomes [26], we tested the significance of lowly divergent SG clustering by random sampling. The significant nonrandom distribution of the lowly divergent SGs in these blocks supported inter-subspecies introgression (Fig. 2c, Additional file 1: Fig. S15). Additionally, divergence measured by synonymous substitution rate (*K*_s_) between XI and GJ genes in the putative introgression blocks (IBs) was significantly lower than that of genes in adjacent genomic regions (Additional file 1: Fig. S16).

Most domestication genes (9/10) underlying key domestication syndromes were found in the IBs (Additional file 1: Fig. S15, Additional file 2: Table S3). On chromosome 4, four IBs larger than 300 Kbp were found, including *LABA1* and *An-1* responsible for awn presence-and-absence and length in blocks 4#1 and 4#2, *sh4* for grain shattering in 4#3, and *Bh4* for hull color in 4#4 (Fig. 2a). *GW5* from block 5#3 is a quantitative trait locus (QTL) for grain width and weight. *Prog1* was located in block 7#1, underlying the transition from prostrate plant architecture to erectness. *GAD1*, encoding an awn development-related secreted peptide, and *IPA1*, which is considered a typical improvement gene controlling ideal plant architecture and immunity [12], were both located in block 8#1. Interestingly, except for *IPA1*, no other improvement genes were found in the IBs, suggesting that introgression events occurred in the initial period of domestication (Additional file 2: Table S2). The largest IB 5#1, with a length of 4.48 Mbp, spanned the centromere region on chromosome 5 (Additional file 1: Fig. S15). The low divergence between XI and GJ in block 5#1 was probably due to suppressed recombination over the centromere region rather than hitchhiking effect under selection, given that no known domestication-related genes were found, which provides an ideal clue of introgression.

**Fig. 3:**
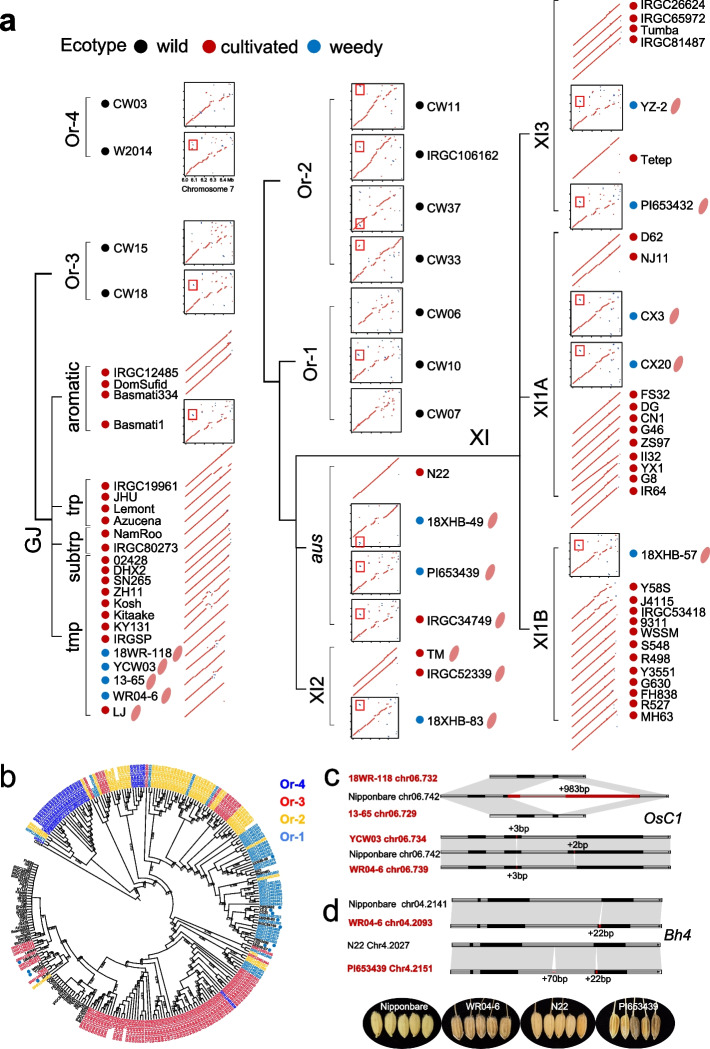
Structural variations in rice de-domestication. **a** Dot plots comparing all 74 assemblies against the de-domestication genomic island (from 6.0 to 6.5 Mbp) on chromosome 7 of Nipponbare. The predominant translocations in wild and weedy accessions are highlighted by red boxes. The pink seed icons behind the accession IDs represent red or brown pericarp. **b** Phylogeny revealed by SNPs in the *Rc* region indicates introgression from Or-1 and Or-3 to XI and GJ, respectively. Labels with green, red, yellow, and blue represent accessions from Or-4, Or-3, Or-2, and Or-1, respectively. Blue dots represent weedy accessions. The numbers on each branch indicate bootstrap values of less than 90%, based on 1000 replicates. **c** Structural variations in *OsC1* between the weedy and cultivated GJ accessions. Black rectangles represent exons. **d** Structural variations in *Bh4* between weedy and cultivated accessions and their seed appearances

High-depth sequencing wild genomes were used to confirm the introgression and trace the spread of domestication haplotypes (Additional file 1: Fig. S17). The introgression inference in 64 blocks (96.2% in length) was supported by phylogeny (Additional file 2: Table S3). Phylogenetic trees of these blocks (including all 18 blocks longer than 300 Kbp) indicated that GJ and XI were nested within Or-3 group, indicating that Or-3 was the shared wild progenitor of GJ and XI in most IBs and that the domesticated alleles in XI were likely to be derived from proto-GJ by introgression to local wild Or-1 rice (Fig. 2d, Additional file 2: Table S3). Several introgression events from cultivated to wild rice were observed, as the presence of wild accessions within domesticated clade [27]. It was noteworthy that between the major clade of domestication haplotypes and Or-3 clade, some wild accessions from Or-1 or Or-2 group were observed (Fig. 3d). We speculate that a gene pool under early domestication was introduced into South Asia and was partially maintained in the genomes of present Or-1 or Or-2 wild rice as relict alleles of proto-GJ.

Statistical ABBA-BABA tests were also performed to confirm the introgression inference. High *f*_d_ values in 57 IBs (21.2Mbp in length) were calculated in the models with introgression direction from tmp to XI groups (Additional file 1: Figs. S18 and S19). By combing haplotype inference, phylogeny, and ABBA-BABA test, 65 blocks (22.6 Mbp in length) were finally determined as introgression regions from GJ to XI (Fig. 2e, Additional file 2: Table S3). Auxin-related pathways were significantly enriched in the IBs (Additional file 2: Table S4). Besides well-known domestication genes for awn presence, shattering, and tiller angle, many seed dormancy and germination related genes were located in the blocks (Additional file 2: Table S5). For example, block 3#5 included a seed dormancy-related gene *OsG*, which has been parallelly selected in multiple crops [28], and *qLTG3-1*, a major QTL controlling low-temperature germinability [12]. *OsC1*, which regulates hull pigmentation and pre-harvest sprouting, was located in block 6#3 (Wang et al., 2020). Yield-related genes included at least *HOX3* (1#2), *SPL6* (3#6), *GPA3* (3#6), *OsNAC2* (4#4), *D11* (4#7), and *FZP* (7#5).

We noticed that the *aus* group exhibited differences from GJ and XI groups in the most IBs. Phylogeny revealed that XI and *aus* belonged to two subclades, although both were nested within Or-1 group (Additional file 1: Fig. S17a). There were 32 blocks where the closest wild group to *aus* was Or-3, while in the other 24 and 8 blocks, Or-1 and Or-4 were the wild progenitors of *aus*, respectively (Fig. 2f). Similarly, only 23 blocks showed gene flow signals from GJ to *aus* in the ABBA-BABA tests (Additional file 1: Fig. S18, Additional file 2: Table S3). Therefore, we speculate that the introgression from proto-GJ or Or-3 to XI probably occurred after the divergence between XI and *aus*, and some domesticated alleles were subsequently transferred from XI to *aus*. Combining the evidence from whole-genome pairwise synteny (Fig. 1b), the *aus* group differs from other XI groups and has a novel evolutionary process.

### Structural variations during rice de-domestication

Besides crop domestication and improvement process, de-domestication or feralization has drawn increasing interest recently [13]. Previous studies have found a 0.5-Mbp de-domestication genomic island that plays essential roles in rice feralization, containing gene *Rc*, responsible for the red pericarp, and a cluster of seed storage-related genes [16]. Genomic landscape of XI weedy rice in this region showed distinct patterns against their corresponding closest cultivated genomes but similar as wild genomes (Fig. 3a). A remarkable 10-Kbp translocation (including one gene encoding an RNA polymerase II transcription subunit) was found in all XI weedy rice but was absent in XI cultivated rice (except cultivar IRGC34749 with a red pericarp) and GJ accessions (except Basmati1) (Fig. 3a). Thus, it was speculated that genomic introgression of the de-domestication genomic island from wild rice contributed to the feralization of XI cultivated rice. A phylogenetic tree based on SNPs around the *Rc* region further supported the introgression and suggested that Or-1 group from Southeast Asia was the ancestral progenitor of the de-domestication genomic island in XI weedy rice (Fig. 3b). For GJ weedy rice, no obvious signals of wild introgression were found (Fig. 3a). However, from the phylogeny of *Rc*, weedy accessions were clustered together and nested within some Or-3 accessions, implying that Or-3 may be the donor of the *Rc* haplotype underlying red pericarp (Fig. 3b). However, it should be noted that some cultivated rice also showed red pericarp (e.g., LJ from GJ), suggesting another potential origin from local landraces of red rice.

We compared the genomic sequences between weedy rice and their phylogenetically closest cultivars (Fig. 1b). Averagely 73,111 small insertions and deletions (InDels, ≤ 50 bp) and 2810 large structural variants (SVs, > 50 bp) were identified in ten weed-cultivar pairs (Additional file 1: Fig. S20). The number of structural variants was lower for GJ weedy-cultivated pairs than those for XI and *aus* pairs. This could owe to recent origin of GJ weedy rice relative to XI and *aus* [16]. XI cultivar NJ11 and its weedy descendant CX20 were reported as a typical case of recent de-domestication events [16]. A minimum number of SVs (*n* = 1299) were detected, where six SVs were larger than 100 Kbp, and the largest one spanned 823 Kbp on chromosome 7 (Additional file 1: Fig. S21). *OsGWD1*, which is involved in transitory starch degradation in source tissues and is also a positive regulator of seed germination, was lost in the CX20 genome, which may be related to the poor eating quality of weedy rice [29].

Totally 3614 SGs were found to have structural variations in at least four de-domestication pairs, implying potential convergent genetic mechanisms underlying feralization. Besides the previously known 14-bp PAV in *Rc*, SVs were found in other domestication-related genes regulating seed shattering, dormancy, and hull color, of which most were located in regulatory regions (Table S6). For shattering, a 2-bp insertion in exon 1 of *sh4*, and a 12-bp deletion in exon 1 and a 146-bp deletion in exon 6 of *SHAT1* were found in *aus* and XI weedy genomes. For seed dormancy, *OsC1*, a R2R3-MYB transcriptional regulator that interacts with *Rc* and *OsVP1*, plays important roles in regulating rice preharvest sprouting tolerance [30]. Two different variants in *OsC1* were found in GJ weedy accessions, which were a 983-bp deletion resulting in incompleteness of exons in accession 18WR-118 from Korea and 13–65 from Italy, and a 3-bp insertion combined with a 2-bp deletion in accessions YCW03 and WR04-6 from China (Fig. 3c, Additional file 1: Fig. S22). A 22-bp insertion in *Bh4* was found in both GJ weedy genome (WR04-6) and *aus* weedy genome (PI653439), which corresponded to their seed hull phenotypes (Fig. 3d, Additional file 1: Fig. S22). The 22-bp PAV was under selection during rice domestication [31]. The phylogeny of *Bh4* confirmed that the 22-bp PAV in weedy rice was likely derived from different groups of wild rice (Additional file 1: Fig. S23). Briefly, although structural differences could be found between weedy and cultivated rice, almost no single causative mutation could explain the convergent phenotypic change for different weedy rice groups, with the only exceptional being *Rc* for red pericarp and seed dormancy; in other words, weedy rice from different groups may have experienced independent evolution after the acquisition of *Rc* from wild rice or local landraces of red rice.

## Discussion

**Fig. 4:**
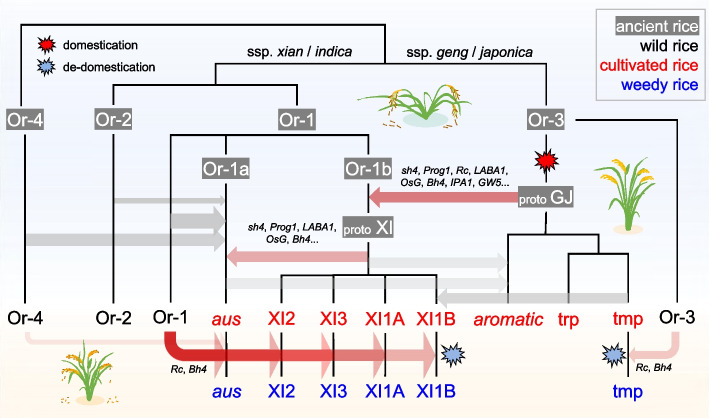
A brief schematic illustration of Asian rice evolution. The evolutionary scenario highlights that complex introgression events have contributed indispensably to rice domestication and de-domestication

There is almost no dispute that rice subspecies have different wild progenitors at whole-genome level (Fig. 4). However, the sources of domestication alleles are hotly debated. For well-known domestication genes, phylogeny analysis has supported single domestication (e.g., *Prog1*, *sh4*, *LABA1*, *Bh4*, *OsC1*) [2, 6], while some wild accessions with domesticated alleles were located within cultivated rice, also observed in this study (Fig. 2d). Gene flow from cultivated to wild rice could be used to explain the sporadic distribution of wild rice within cultivated sub-trees [27]. However, some studies interpreted these as ancestral domesticated alleles in wild rice prior to domestication and highlighted possibility of independent acquisition of domestication alleles from wild populations [10, 32], although this inference seems not reasonable. If these wild alleles emerged before domestication, their phylogenetic positions would not been within cultivated accessions.

It is not enough to conclude the evolutionary trajectory just based on the allele presence of domestication genes [7], and the effects by genetic drift should be considered. Cultivated rice genomes we have sequenced now are only a subset of ancestral proto-GJ or proto-XI genetic pool and cannot fully represent the diversity during domestication. GJ has suffered a dramatic genetic bottleneck around three thousand years ago, while XI shows no obvious decline in effective population size in the last few thousand years [16, 33]. Here, in our phylogeny analysis on putative introgression blocks, some Or-1 or Or-2 accessions were located between domestication clade and Or-3 clade, as relict alleles of proto-GJ and genomic footprints of ancient introgression, which are absent in current GJ gene pool but present in wild Or-1 or Or-2 (Fig. 2d). Therefore, the genetic drift probably played unneglectable roles in the presence of non-GJ domesticated alleles in XI. Indeed, the non-GJ alleles in XI domestication genes have been observed in this study. *Rc* (HDG = 0.803), *An-1* (0.886) and *GW5* (0.875) have high haplotype divergence between XI and GJ, but the genomic regions they are located in showed robust introgression signals (Additional file 2: Tables S2 and S3). Haplotype analysis on a single gene sometimes will mislead the conclusion, due to the gene diversification after domestication.

By taking advantage of 74 high-quality rice genomes, we revisited the domestication processes of rice subspecies and groups from a pan-genome view (Fig. 4). Compared to whole-genome resequencing, genome assembly enables us to analyze full-length sequences of all genetic elements, which eliminates systematic errors caused by read mapping and sequencing depth. We compressed the variations within a gene, providing us with a visual landscape of haplotype similarity among taxa in each SG. Similar approaches using gene haplotypes or blocks have been adopted in recent studies [9, 34]. A haplotype map based on SNPs from coding regions was constructed for 3010 cultivated and 15 wild rice accessions [9]. The inferred proto-ancestors of different groups correlated strongly with wild rice from the same geographic regions, which was considered to support a multi-domestication model of rice. However, in spite of extremely insufficient number of wild rice, the whole-genome haplotype similarity among populations cannot indicate how domesticated alleles come from. Here, by assigning ancestral or dominant haplotypes of each SG, we determined a total of > 20 Mbp genomic regions as putative introgression blocks, which avoided potential mis-assignment by hard thresholds used in clustering based on genetic distance or phylogeny. Such an ancestral genomic haploblock dissection method has also been employed in tracing the origin of polyploid wheat, and mosaic genomic graphs have suggested dispersed emergence and protracted domestication in wheat [34]. In the shared haplotype blocks between GJ and XI, the majority of their phylogenetic trees, combined with the relatively low divergence or genetic distances within these regions, supported the introgression of domestication alleles from proto-GJ to Or-1 group. Interestingly, the low-recombination centromere region on chromosome 5 suggested clear genomic affinity between subspecies, as a relict footprint of ancient introgression (Additional file 1: Fig. S15). Statistical ABBA-BABA tests using high-depth sequenced wild genomes and larger genome sampling also confirmed the reliability of putative introgression blocks (Additional file 1: Fig. S18). Although the haplotype inference based on syntelog protein sequences have lost non-coding variations, the potential in tracing evolutionary trajectory is unnegligible.

Utilizing domestication alleles from other geo-isolated populations or species has facilitated the generation of local domesticates. In wheat, dispersal domestication events generated domesticated alleles or haplotypes for different genes in different locations. Genomic introgression among different populations or species by human activities has gathered domestication alleles of different genes together, leading to the emergence and popularity of hexaploid wheat [34]. Here, our results confirmed the previous hypothesis that genomic introgression of domestication alleles from proto-GJ to wild Or-1 led to the emergence of XI (Fig. 4). Despite limited sampling of the *aus* type due to high requirement in genome quality, we inferred that the ancestral wild population of *aus* was different from that of XI, although they were both clustered within the Or-1 wild group. Genomic introgression from local wild rice and domesticated XI rice may have led to the birth of *aus* rice. More genomes and detailed analysis in the future will uncover the complex evolutionary process of *aus* and other groups (e.g., aromatic).

De-domestication is an atavistic process in domesticates that has been studied in crops (e.g., rice, wheat and sunflower) and livestock (e.g., chicken and dog) [13]. Here, our analysis combining both structural comparison and phylogenetic analysis highlighted the influences of wild introgression on the emergence of weedy rice, despite independent introgression events for GJ and XI (Fig. 4). For the brown hull of weedy accessions, the causative structural mutation was indicated to be derived from the corresponding wild groups Or-1 and Or-3 for XI and GJ, respectively. Although pairwise whole-genome comparison identified thousands of structural variants between weedy and cultivated rice, structural convergence in different weedy-cultivated lineages was seldom observed, which implied the recurrent independent emergence of weedy traits. The mechanism underlying high shattering in weedy rice was still not well resolved from the view of structural variations in known domestication genes. Given that our known shattering-related genes are almost all transcription factors, variations in other regulatory elements or even epigenetic factors may lead to high shattering in weedy rice. Overall, genomic introgression plays an indispensable role throughout the entire evolutionary trajectory of rice, from initial domestication, improvement, modern breeding, and feralization.

## Conclusions

In summary, we performed an integrated analysis across the genomes of wild-to-domesticated-to-weedy continuum to revisit the rice evolutionary trajectory and highlight the significance of introgression in rice domestication and de-domestication. We constructed a syntelog-based pangenome, compressed the variations in units of genes, assigned and merged ancestral haplotypes, and finally determined a total of > 20 Mbp putative introgression regions. We envision that such ancestral genomic haploblock dissection approaches could improve the inference about complex history of crop domestication and dispersal. Our results support the hypothesis of single domestication with multiple origin for rice. We also examined the structural genomic differences between cultivated and weedy genomes and highlighted the influences of wild introgression on the emergence of weedy rice, though weedy genomes were descents of cultivated rice. Overall, we utilized a syntelog-based pangenome to investigate rice evolutionary trajectory.

## Methods

### Genome sequencing and assembly

To capture the genetic diversity from all ecotypes of rice, we collected 11 accessions of weedy rice from seven countries based on their phylogeny with cultivated rice and geographic positions; we also included one XI cultivar NJ11, or Nanjing 11, which is presumed to be the direct ancestor of weedy rice in the Yangtze River Basin [16]. Genomic DNA samples were extracted from young leaves of the 12 rice accessions, and their genomes were sequenced by PacBio HiFi mode according to the instructions from the manufacturer. For each accession, the sequencing depth of HiFi subreads ranged from 21.6 × for accession 13–65 to 45.0 × for accession YZ-2, with an average of 32.4 × . Following the standard protocol, Hi-C libraries of four accessions (NJ11, CX20, YCW03 and 18XHB-83) were constructed using fresh young leaves digested with the 4-cutter restriction enzyme MboI. Hi-C libraries were sequenced on an Illumina HiSeq 4000 platform with 2 × 150-bp paired reads.

The genomes were first assembled using hifiasm (v0.15.1-r334, default parameters) [35]. For each accession, HiFi subreads were mapped against the corresponding assembly, and Purge_dups [36] was applied to purge duplicates and remove redundant sequences according to the mapping depth. We further used Racon (v1.4.0) [37] to polish the assemblies with HiFi subreads for three rounds under default parameters. Contigs less than 10 Kbp were removed from the final version. For each accession, contigs were anchored into pseudochromosomes by using a reference-guiding approach RaGOO [38]. By aligning contigs against the Nipponbare assembly, the contigs were ordered and oriented along 12 chromosomes with no further chimeric splitting.

Previously released rice assemblies based on third-generation sequencing platforms were collected, including PacBio [18, 19, 22, 39–43] and Nanopore sequencing [20, 21, 44]. Before adopting assemblies in the construction of the rice pangenome, we first systematically evaluated the assembly qualities and ruled out assemblies that did not meet our criteria.

### Quality assessment of rice genome assemblies

We first assessed the quality of our 12 newly assembled rice genomes. Synteny against the reference assembly Nipponbare and gapless assembly MH63 [22] confirmed their high completeness. The paired Hi-C reads of four accessions (YCW03, 18XHB-83, CX20 and NJ11) were cleaned and mapped to the corresponding assembly using Bowtie2 (v2.3.5.1) [45]. After retaining high-quality and validated paired reads (mapping quality ≥ 30, edit distance ≤ 5, number of mismatches in the alignment ≤ 3, number of gap opens ≤ 2 and number of gap extensions ≤ 2), chromosome interaction maps were plotted, and they revealed high accuracy in contig ordering and orientation (Additional file 1: Fig. S1).

Genome assemblies of *Oryza sativa* and *Oryza rufipogon* based on third-generation sequencing (through May 2022) were collected (Additional file 2: Table S1). DXCWR [42] was excluded considering its low contig N50 of less than 200 Kbp. IRGC109232 [17] was removed due to the abnormal size of the assembly obtained from the public database. Eleven assemblies in Zhang et al. (2022) [20] were randomly selected from 75 newly generated genomes and used in subsequent quality assessment. Five indices were applied to evaluate the genome quality of all rice assemblies (Additional file 1: Fig. S2a). Assembly continuity was evaluated by contig N50 and LAI, which was revealed by the assembly completeness of long-terminal-repeat (LTR) retrotransposons [46]. The LTR elements of each assembly were identified by RepeatMasker and RepeatModeler (http://repeatmasker.org/). BUSCO (v4.1.2) [47] metrics were calculated to evaluate the completeness by using dataset poales_odb10 containing 4896 genes. As expected, assemblies based on long-read sequencing performed well on the above quality indices (Additional file 1: Fig. S2a). Hence, in addition to the above evaluations at the whole-genome level, base-resolution accuracy and completeness were measured by QVs and the number of homozygous variants called by self-short-read mapping. Reference-free QVs were calculated by Merqury [48] and yak (https://github.com/lh3/yak) by comparing *k*-mers derived from unassembled, high-accuracy sequencing reads to a genome assembly. Homozygous variants (SNPs and InDels) called with short reads by self-mapping are regarded as potential assembly errors. Variants with high effects (including stop loss and gain, start loss, frame-shift variant) and moderate effects (including in-frame insertion/deletion, missense variant) will directly impact the reliability of gene haplotypes and downstream haplotype analysis. The assembly quality of wild accessions from Shang et al. (2022) [21] and one weedy accession (YCW03) were not assessed at the base level due to the unavailability of NGS data. Assemblies with low base-level quality were removed for cultivated rice, mainly including Nanopore sequencing-based genomes, except three accessions (DomSufid, Basmati334 and JHU) from aromatic and tropical groups of GJ (*O. sativa* ssp. *japonica*), which were kept to balance the sampling of each group. For wild genomes, only two accessions, W2014 from Ma et al. (2020) [42] and IRGC106162 from Xie et al. (2021) [43], sequenced using the PacBio platform were available. Thus, nine assemblies in different wild groups from Shang et al. (2022) [21] sequenced by the Nanopore platform were adopted for analysis.

### Phylogenetic relationship of rice assemblies

Together with the 12 new assemblies in this study, a total of 75 rice assemblies, including 11 wild accessions (*O. rufipogon*), 12 weedy accessions (*O. sativa* ssp. *spontanea*), 51 cultivated accessions (*O. sativa*), and an African cultivated rice outgroup (*Oryza glaberrima*) accession CG14, were used in this study. We first confirmed their phylogenetic relationship and assigned them to taxonomic groups using whole-genome SNPs. The assemblies were aligned against the reference assembly Nipponbare using nucmer implemented in the MUMmer package (v4.0.0) [49], and called SNPs were used to build their phylogeny by FastTreeMP [50] with 1000 bootstrap replicates. According to their phylogeny and prior knowledge of the 74 *Oryza sativa* and *Oryza rufipogon* accessions, each accession was assigned to groups. Briefly, the subspecies GJ (*O. sativa* ssp. *japonica*, *n* = 23 in total) includes four *aromatic* (aro), four tropical (trp), two subtropical (subtrp), and 13 temperate (tmp) accessions; subspecies XI (*O. sativa* ssp. *indica*, *n* = 40) includes four *aus*, three XI2, seven XI3, ten XI1A, and 16 XI1B accessions. The wild population (*O. rufipogon*, *n* = 11) includes two accessions from Or-3, four from Or-2, three from Or-1, and two from Or-4. Genotype data for approximately seven thousand rice accessions used in PCA were adopted from a previous study [51]. PCA and IBD (Identity-by-descent) calculation were performed using Plink (v1.9) [52] with a pruned subset of SNPs based on linkage disequilibrium (10 SNPs in each 50-Kbp sliding window with pairwise Pearson’s correlation efficient *r*^2^ less than 0.5).

### Genome annotation

In total, 75 rice assemblies (including African rice CG14 as an outgroup) were annotated in a unified pipeline. First, transposon elements (TEs) were identified by using the Extensive de novo TE Annotator (EDTA) approach (https://github.com/oushujun/EDTA). For each accession, gene models were predicted on the repeat-masked genome using an approach integrating ab initio predictions and homology-based prediction. For ab initio prediction, Augustus [53] and Fgenesh [54] were performed with default parameters. For homology-based prediction, previously predicted protein sequences of the Nipponbare (IRGSP v1.0), gapless MH63RS3, and ZS97RS3 assemblies [22] and over 30 other well-annotated assemblies [18] were used to search putative protein-coding gene models. The predictions were integrated into non-redundant consensus gene models using EVidenceModeler (v1.1.1) [55]. Short gene models (less than 50 amino acids) and gene models with homology to sequences in Repbase (*e*-value ≤ 1e − 5, identity ≥ 30%, coverage ≥ 25%) were further removed from the final annotation. The protein domains of all predicted coding gene models were inferred using InterProScan (v5.24–63.0) [56].

### Pan-genome construction using MCL

A Markov clustering (MCL) approach OrthoFinder (v2.4.1) [57] was applied to cluster all the predicted gene models of 74 rice genomes (African rice accession CG14 was excluded) with default parameters (diamond all-versus-all *e*-value < 1e − 5 and inflation parameter = 1.5). Finally, over 3.10 million predicted genes were clustered into 67,080 orthogroups (OGs). Increasing the inflation parameter can be used to achieve higher precision at the cost of lower recall and have a larger size of OGs. Conversely, a smaller value of the inflation parameter could achieve higher recall at the cost of lower precision and produce smaller sized OGs, which easily clusters paralogs together [57]. Thus, three additional inflation parameters (*I* = 1.8, 2.0 and 2.5) were set to profile the effects on clustering (Additional file 1: Fig. S9). Clustered gene families were categorized into core (present in all genomes, *n* = 74), soft-core (present in at least 90% of genomes, *n* = 67 to 73), dispensable (present in more than one but less than 90% of genomes, *n* = 2 to 66), and private (only present in one genome) on the basis of the number of rice accessions in which they were identified. When the inflation parameter is 1.5, totally 15,749 core, 11,725 soft-core, 18,901 dispensable, and 20,705 private OGs were classified.

### Pan-genome construction using synteny

Given that the MCL approach does not efficiently and accurately distinguish paralogs from orthologs with high sequence similarity, we utilized the availability of genomic coordinates of gene models to build the synteny-based pan-genome. Pairwise all-to-all alignments using protein sequences were performed for all 74 genomes. We first compared the performance of aligner Diamond [58] and BLASTP in determining syntenic orthologs or syntelogs. Diamond runs much faster for large protein sequence data than BLASTP. We compared the recall performance between BLASTP and Diamond in detecting syntelogs between genomes (Additional file 1: Fig. S6). Diamond was run for each pair under three modes (default, sensitive and ultra-sensitive). The alignments were filtered to keep only the best hits, and DAGchainer [59] was used to detect syntenic genomic regions and syntelogs (parameters: -Z 12 -D 200000 -g 1 -A 5). No differences were observed among the three Diamond modes in the number of detected syntelogs (Additional file 1: Fig. S6). BLASTP was performed under default parameters, and syntelogs were identified using the same pipeline. The BLASTP approach searched more syntelogs (with an average addition of 669.1 pairs per genome alignment), and thus, the syntelogs using BLASTP were used in downstream pan-genome construction (Additional file 1: Fig. S6). Instead of a reciprocal best hit search, the roles of reference and query in a pairwise BLASTP search sometimes result in differences due to individual-specific tandem duplicates, and such individual-specific tandem duplicates were considered as a single gene in the final pan-genome. Synteny kinship was calculated as the proportion of syntelogs in all annotated genes of reference genomes. Pairwise syntelog information was then merged together with Nipponbare as an initial framework one by one using SynPan (https://github.com/dongyawu/PangenomeEvolution). If a gene from an additional genome was syntenic to a previously merged pan-genome, this gene was assigned to an existing SG. If a gene from an additional genome was not syntenic to any gene in the merged iterative pan-genome, a new SG was created. In total, 74 genomes were merged together as a synteny-based pan-genome, including 175,528 SGs. The SGs were further categorized as core, soft-core, dispensable and private following the criteria used in OG.

### Construction of the rice NLRome

To capture the diversity of NLRs in rice, we integrated multiple software predictions and gene synteny in rice genomes to obtain a comprehensive and complete rice NLRome (Additional file 3: Supplemental Note 2). NLR genes were defined to have at least one NB-ARC, TIR, or CCR (RPW8) canonical domain. LRR or CC motifs alone were not considered sufficient for NLR identification. Finally, NLRs in rice genomes were identified and categorized as CNLs (containing CC, NB-ARC, and LRR domains), CNs (containing CC and NB-ARC domains), and NLs (containing only canonical NB-ARC domains). Non-canonical architectures of some NLRs have additional integrated domains (IDs), while canonical architectures contain only NB-ARC (Pfam ID PF00931), TIR (PF01582), RPW8 (PF05659), or LRR (PF00560, PF07725, PF13306, PF13855) domains or CC motifs. For ID identification, we used the genome protein sequences as the input for InterProScan, and annotations were processed with in-house scripts to obtain ID information. There are currently several known cases in which two NLR genes are required to affect the resistance function, with one protein in the pair acting in effector recognition and the other acting in signaling activation. Since all known functional pairs are present in head-to-head arrangement in the rice genome, we identified head-to-head NLR pairs by searching for NLR genes less than 10 Kbp away.

### Ancestral haplotype assignment

To understand the haplotype-aware origin of genes from different rice groups, we inferred the ancestral haplotype composition for each SG by determining the dominant haplotype in each rice group and assigning ancestral haplotype IDs for all genes in a group priority-based referring strategy (Additional file 1: Fig. S14a). Group information is prior based on whole-genome phylogeny or population structure. We labeled five haplotype IDs (hapI to hapV) to represent the dominant haplotypes in groups tmp, XI1A, XI1B, *aus*, and XI3 in order, presented by red, blue, orange, yellow, and green in Fig. 4a and Additional file 1: Fig. S15, respectively. HapI was first defined as the most dominant sequence in group tmp. If the dominant haplotype in the current group was defined by a former group, the haplotype ID was skipped and set as missing. For example, if the dominant haplotype in XI1A was the same as that in tmp (hapI), hapII was then not defined. The frequency of a dominant haplotype within a group should be at least three. All other rare haplotypes were compressed as hapR in gray to simplify the ancestral haplotype graphs. Gene absence is represented by white blocks. Different group priority orders have no influences on the following calculation of haplotype divergence but only change the ancestral haplotype graphs (Additional file 1: Fig. S14c). A total of 49,438 SGs whose syntelog members were present in at least ten genomes were analyzed.

### Inter-subspecies introgression blocks

As observed from the ancestral haplotype landscape, haplotypes in some large genomic regions were shared between XI and GJ. We used inter-population haplotype divergence (HDG) to quantify haplotype sharing by calculating the average differences in haplotypes from the two populations (Additional file 1: Fig. S14b). As observed from the mosaic genomic map, *aus* showed obvious differences from the other XI groups; thus, the *aus* group was excluded from XI in the GJ-XI introgression analysis (Fig. 2a, Additional file 1: Fig. S15). We merged adjacent SGs whose divergence values between tmp and XI (excluding *aus*) were less than 0.5 into lowly divergent blocks between subspecies as candidate introgression blocks. At least 10 SGs were required within a single introgression block. To examine the significance of the nonrandom clustered distribution of these lowly divergent SGs in blocks, we randomly sampled the same number of SGs as lowly divergent SGs observed on each chromosome and calculated the density of sampled SGs in sliding windows of every 10 SGs. A total of 100 thousand random samplings were replicated, and the *P* values were determined by counting the sampling times where the density of sampled SGs was higher than that observed for each window. *P* = 0.01 was empirically set as a cutoff value. Assuming that the low divergence in the detected blocks was caused by inter-subspecies introgression after their divergence, the divergence time between GJ and XI should be younger in the identified blocks than in their neighboring regions. We used the synonymous substitution rate (*K*_s_) to measure the relative divergence time between GJ and XI, independent of selection. Significantly lower *K*_s_ values were observed in the majority of 18 large blocks (> 300 Kbp) than in their flanking regions (Additional file 1: Fig. S16).

### Phylogeny of introgression blocks

To investigate the origin and gene flow of the 73 candidate introgression blocks, we utilized recently released genomic sequences of 184 wild accessions with high sequencing depth (8 × on average, much higher than < 2 × on average in a previously used wild population) [2, 60]. Raw sequencing reads were first cleaned and mapped against the reference assembly Nipponbare (IRGSP v1.0) using Bowtie2. Assemblies of two wild rice accessions (W1943 and DWCWR) from group Or-3 were added. Combing the 77 assemblies and 184 wild genomes, high-quality SNPs were called following a previous pipeline [16]. The population structure was first surveyed by PCA using Plink and the phylogeny was produced by FastTreeMP with 1000 bootstrap replicates based on 6.85 million high-quality SNPs (minor allele frequency of 0.02 and maximum missing rate of 0.1). Four wild groups were identified: Or-1, Or-2, Or-3, and Or-4. The SNPs in each introgression block were extracted and used to build the phylogeny using IQ-TREE (v1.6.12) with the best substitution model TIM2e + R2 determined by ModelFinder implemented in IQ-TREE [61] and FastTreeMP with 1000 bootstrap replications, where the African cultivated rice accession CG14 was set as the outgroup. To avoid over-interpretation, the phylogeny in which the wild accessions were not obviously and empirically clustered into four groups, as defined by the whole-genome SNPs, was discarded.

### ABBA-BABA test

The available high-depth sequencing genomic data of wild rice from four groups (Or-1, Or-2, Or-3, and Or-4), enables us to perform comprehensive statistical *D* tests, a widely used and robust method for gene flow detection [26, 62]. We randomly selected genomes of XI1A rice accessions (*n* = 100), XI1B (*n* = 100), XI2 (*n* = 80), XI3 (*n* = 100), and *aus* (*n* = 60) from 3 K RGP [7]. We employed *f*_d_ statistic to indicate the introgression from GJ to XI in sliding genomic windows [63]. Under a given quartet topology ((P1, P2), P3, O), positive *f*_d_ statistic values indicate the introgression from P3 to P2, zero represents no introgression, and negative *f*_d_ statistic values have no biological meaning and thus are converted to zero. We estimated the *f*_d_ statistic values under topology ((Or-1, *X*), tmp, Or-4), where *X* is Or-2, XI1A, XI1B, XI2, XI3, and *aus* in topology T1 to T6, respectively (Additional file 1: Fig. S18). T1 is set as a background control in detecting introgression. To eliminate the influence of modern breeding where inter-subspecies hybridization is frequently performed on ancient introgression inference, we used genomes of only landraces in each group to repeat the *f*_d_ calculation, where tmp, XI1A, XI2, XI3, and *aus* included 47, 24, 21, 52, and 32 landrace accessions, respectively. Python scripts are available at https://github.com/simonhmartin/genomics_general. The parameters were set as “window size: 20 Kbp, step size: 2 Kbp, minimum good sites per window: 50, and minimum proportion of samples genotyped per site: 0.4.” The final putative introgression regions were determined by integrating evidences form haplotype divergence, phylogeny, and ABBA-BABA tests. The functional enrichment analysis was performed using ShinyGO (v0.77) [64].

### Structural variations in de-domestication

To gain a more detailed understanding of structural variations in de-domestication, we compared the genome assemblies of weedy and cultivated rice. It has been found that the sensitivity of detecting deletions is higher than that of insertions, and we adopted a pairwise genome alignment strategy as mentioned previously in Jayakodi et al. (2020) [65]. Each pair contains a weedy accession and a cultivar accession, which compose a monophyly in the phylogenetic tree, and the genome assembly of cultivated rice was considered a query or reference genome. Nucmer in the MUMmer package was used to obtain the results of these two alignments, and then PAVs (presence-and-absence variants, including insertions and deletions) were called using Assemblytics (v.1.2.1) [66]. The structural PAVs were classified as InDels (≤ 50 bp) and SVs (> 50 bp). Only deletions were kept in both alignments and converted into PAVs according to the reference genome in each pair. Genes located in or intersected with each PAV were obtained, as well as corresponding gene annotations. The variations in domestication genes and their flanking regions (15 Kbp for *qSH1* and 2 Kbp for other genes) were manually investigated, and the causative mutations during the domestication process were checked. To validate the reliability of structural variations in domestication or improvement genes (e.g., *OsC1* and *Bh4*), HiFi subreads of weedy rice were mapped against themselves and corresponding cultivated assemblies to check the local alignments using IGV (https://igv.org/). To infer the source of the causative mutation in *Bh4* in weedy accessions, the *Bh4* phylogeny based on SNPs was analyzed.

The 73 rice genome assemblies were aligned against the Nipponbare reference genome using the nucmer program implemented in the MUMmer package with the default parameter, and only the best position of each query on the reference was preserved. The alignments from 6.0 to 6.5 Mbp on chromosome 7 in the Nipponbare assembly were extracted for visualization by synteny plots and comparison among wild, cultivated, and weedy assemblies. To validate the candidate introgression event inferred from the synteny plots, SNPs around the *Rc* gene (including its flanking 2-Kbp regions) were extracted and used in phylogeny construction by FastTreeMP under the GTR + CAT model with 1000-times bootstrapping.

### Supplementary Information

**Additional file 1: Fig. S1.** Hi-C interaction heatmaps for each chromosome from four rice accessions. **Fig. S2.** Statistical information of the rice genomes used in this study. **Fig. S3.** Dot plots of newly generated rice assemblies in this study against the reference assembly Nipponbare (IRGSP) and gapless assembly MH63RS3. **Fig. S4.** Assembly quality assessment in base accuracy. **Fig. S5.** PCA reveals the representativeness and diversity of genome assemblies used in this study. **Fig. S6.** Performance of BLASTP and Diamond (under different modes) in syntelog identification. **Fig. S7.** Pairwise synteny reveals evolutionary signatures in groups and individuals. **Fig. S8.** Comparison of MCL and synteny-based clustering. **Fig. S9.** Benchmarking analysis on the influences of inflation parameters in the MCL clustering in rice genomes. **Fig. S10.** Composition and features of the rice syntelog-based pangenome. **Fig. S11.** Comparison of NLR genes in rice genomes from different ecotypes and subspecies. **Fig. S12.** Genomic features of NLRs in rice genomes. **Fig. S13.** Frequency differences in gene presence between GJ and XI along chromosome 7. **Fig. S14.** Haplotype analysis on rice syntelogs. (a) Definition of haplotype diversity and divergence. **Fig. S15.** Ancestral haplotype landscape on chromosome 1 to chromosome 12. **Fig. S16.** Synonymous substitution rates (Ks) of genes in putative introgression blocks and their neighboring left and right regions. **Fig. S17.** Population structure of wild rice accessions used in this study.** Fig. S18.** Introgression fd distributions of ABBA-BABA test on chromosome 1 to chromosome 12. **Fig. S19.** Comparison of fd using all genomes and landraces only under different topologies on chromosome 1. **Fig. S20.** Summary of structural variations between weedy and cultivated rice genomes. **Fig. S21.** Structural variations (>50 bp) between assemblies of cultivar accession NJ11 and weedy rice accession CX20 on 12 chromosomes. **Fig. S22.** The Integrative Genomics Viewer (IGV) snapshots show the structural variations in OsC1 and Bh4 between weedy and cultivated rice. **Fig. S23.** Phylogeny of Bh4 in rice and morphology of rice seed hulls. Bootstrap values less than 0.90 are indicated on branches.

**Additional file 2: Table S1.** Meta information of 75 rice genome assemblies used in this study. **Table S2.** Haplotype divergence between XI and GJ for domestication and improvement genes. **Table S3.** Putative introgression blocks identified by haplotype divergence between GJ and XI and validation by phylogeny and ABBA-BABA tests. **Table S4.** Gene functional enrichment in final putative introgression blocks. **Table S5.** Cloned genes in the putative introgression blocks. **Table S6.** Structural variations between each pair of weedy and cultivated accessions in agronomy-related gene.

**Additional file 3: Supplemental Note 1.** Base-level quality evaluation of rice genomes. **Supplemental Note 2.** Rice NLRome.

**Additional file 4.** Review history.

## Data Availability

All the PacBio HiFi subreads for 12 rice accessions and Hi-C data for four rice accessions generated in this study have been deposited in NGDC (https://ngdc.cncb.ac.cn/) under the accession code PRJCA012143 [67]. The newly generated assemblies for 12 accessions and the annotations (including GFF, CDS sequences and predicted protein sequences) for all 74 rice accessions can be found under project accession PRJCA012309 in NGDC [68]. The raw resequencing data of previously published wild accessions can be downloaded from NCBI under accession number PRJNA657701 [69]. The VCF file of SNPs from all 75 assemblies and an additional 184 wild rice accessions with high sequencing depth is available at Zenodo (https://doi.org/10.5281/zenodo.7196576) [70] and also deposited with the project accession number PRJCA018336 in NGDC [71]. The gene re-annotations, NLR annotations, and Pfam annotations for all 75 rice accessions are deposited at Zenodo (https://doi.org/10.5281/zenodo.7248110) [72]. The in-house scripts used in this study have been deposited under MIT license in GitHub (https://github.com/dongyawu/PangenomeEvolution) [73] and in Zenodo (https://doi.org/10.5281/zenodo.8157689) [74].

## References

[1] Molina J, Sikora M, Garud N, Flowers JM, Rubinstein S, Reynolds A (2011). Molecular evidence for a single evolutionary origin of domesticated rice. Proc Natl Acad Sci U S A.

[2] Huang X, Kurata N, Wei X, Wang Z, Wang A, Zhao Q (2012). A map of rice genome variation reveals the origin of cultivated rice. Nature.

[3] Civáň P, Craig H, Cox CJ, Brown TA (2015). Three geographically separate domestications of Asian rice. Nat Plants.

[4] Gross BL, Zhao Z (2014). Archaeological and genetic insights into the origins of domesticated rice. Proc Natl Acad Sci U S A.

[5] Choi JY, Platts AE, Fuller DQ, Hsing Y, Wing RA, Purugganan MD (2017). The rice paradox: multiple origins but single domestication in Asian rice. Mol Biol Evol.

[6] Choi JY, Purugganan MD (2018). Multiple origin but single domestication led to Oryza sativa. G3.

[7] Wang W, Mauleon R, Hu Z, Chebotarov D, Tai S, Wu Z (2018). Genomic variation in 3,010 diverse accessions of Asian cultivated rice. Nature.

[8] Carpentier M, Manfroi E, Wei F, Wu H, Lasserre E, Llauro C (2019). Retrotranspositional landscape of Asian rice revealed by 3,000 genomes. Nat Commun.

[9] Zhang F, Wang C, Li M, Cui Y, Shi Y, Wu Z (2021). The landscape of gene-CDS-haplotype diversity in rice: properties, population organization, footprints of domestication and breeding, and implications for genetic improvement. Mol Plant.

[10] Civáň P, Brown TA (2018). Misconceptions regarding the role of introgression in the origin of Oryza sativa subsp. indica. Front Plant Sci.

[11] Chen E, Huang X, Tian Z, Wing RA, Han B (2019). The genomics of Oryza species provides insights into rice domestication and heterosis. Annu Rev Plant Biol.

[12] Chen R, Deng Y, Ding Y, Guo J, Qiu J, Wang B (2022). Rice functional genomics: decades’ efforts and roads ahead. Science China Life Sciences.

[13] Wu D, Lao S, Fan L (2021). De-domestication: an extension of crop evolution. Trends Plant Sci.

[14] Song B, Chuah T, Tam SM, Olsen KM (2014). Malaysian weedy rice shows its true stripes: wild Oryza and elite rice cultivars shape agricultural weed evolution in southeast Asia. Mol Ecol.

[15] Li L, Li Y, Jia Y, Caicedo AL, Olsen KM (2017). Signatures of adaptation in the weedy rice genome. Nat Genet.

[16] Qiu J, Jia L, Wu D, Weng X, Chen L, Sun J (2020). Diverse genetic mechanisms underlie worldwide convergent rice feralization. Genome Biol.

[17] Zhao Q, Feng Q, Lu H, Li Y, Wang A, Tian Q (2018). Pan-genome analysis highlights the extent of genomic variation in cultivated and wild rice. Nat Genet.

[18] Qin P, Lu H, Du H, Wang H, Chen W, Chen Z (2021). Pan-genome analysis of 33 genetically diverse rice accessions reveals hidden genomic variations. Cell.

[19] Zhou Y, Chebotarov D, Kudrna D, Llaca V, Lee S, Rajasekar S (2020). A platinum standard pan-genome resource that represents the population structure of Asian rice. Sci Data.

[20] Zhang F, Xue H, Dong X, Li M, Zheng X, Li Z (2022). Long-read sequencing of 111 rice genomes reveals significantly larger pan-genomes. Genome Res.

[21] Shang L, Li X, He H, Yuan Q, Song Y, Wei Z (2022). A super pan-genomic landscape of rice. Cell Res.

[22] Song J, Xie W, Wang S, Guo Y, Koo D, Kudrna D (2021). Two gap-free reference genomes and a global view of the centromere architecture in rice. Mol Plant.

[23] Long Y, Zhao L, Niu B, Su J, Wu H, Chen Y (2008). Hybrid male sterility in rice controlled by interaction between divergent alleles of two adjacent genes. Proc Natl Acad Sci U S A.

[24] Zhan C, Lei L, Liu Z, Zhou S, Yang C, Zhu X (2020). Selection of a subspecies-specific diterpene gene cluster implicated in rice disease resistance. Nat Plants.

[25] Tseng I, Hong C, Yu S, Ho TD (2013). Abscisic acid- and stress-induced highly proline-rich glycoproteins regulate root growth in rice. Plant Physiol.

[26] Wu D, Shen E, Jiang B, Feng Y, Tang W, Lao S (2022). Genomic insights into the evolution of Echinochloa species as weed and orphan crop. Nat Commun.

[27] Wang H, Vieira FG, Crawford JE, Chu C, Nielsen R (2017). Asian wild rice is a hybrid swarm with extensive gene flow and feralization from domesticated rice. Genome Res.

[28] Wang M, Li W, Fang C, Xu F, Liu Y, Wang Z (2018). Parallel selection on a dormancy gene during domestication of crops from multiple families. Nat Genet.

[29] Wang Z, Wei K, Xiong M, Wang JD, Zhang CQ, Fan XL (2021). Glucan, Water-Dikinase 1 (GWD1), an ideal biotechnological target for potential improving yield and quality in rice. Plant Biotechnol J.

[30] Wang J, Deng Q, Li Y, Yu Y, Liu X, Han Y (2020). Transcription factors Rc and OsVP1 coordinately regulate preharvest sprouting tolerance in red pericarp rice. J Agric Food Chem.

[31] Zhu B, Si L, Wang Z, Jingjie Zhu YZ, Shangguan Y, Lu D (2011). Genetic control of a transition from black to straw-white seed hull in rice domestication. Plant Physiol.

[32] Civáň P, Brown TA (2017). Origin of rice (Oryza sativa L.) domestication genes. Genet Resour Crop Evol.

[33] Gutaker RM, Groen SC, Bellis ES, Choi JY, Pires IS, Bocinsky RK (2020). Genomic history and ecology of the geographic spread of rice. Nat Plants.

[34] Wang Z, Wang W, Xie X, Wang Y, Yang Z, Peng H (2022). Dispersed emergence and protracted domestication of polyploid wheat uncovered by mosaic ancestral haploblock inference. Nat Commun.

[35] Cheng H, Concepcion GT, Feng X, Zhang H, Li H (2021). Haplotype-resolved de novo assembly using phased assembly graphs with hifiasm. Nat Methods.

[36] Guan D, McCarthy SA, Wood J, Howe K, Wang Y, Durbin R (2020). Identifying and removing haplotypic duplication in primary genome assemblies. Bioinformatics.

[37] Vaser R, Sović I, Nagarajan N, Šikić M (2017). Fast and accurate de novo genome assembly from long uncorrected reads. Genome Res.

[38] Alonge M, Soyk S, Ramakrishnan S, Wang X, Goodwin S, Sedlazeck FJ (2019). Ragoo: fast and accurate reference-guided scaffolding of draft genomes. Genome Biol.

[39] Du H, Yu Y, Ma Y, Gao Q, Cao Y, Chen Z (2017). Sequencing and de novo assembly of a near complete indica rice genome. Nat Commun.

[40] Sun J, Ma D, Tang L, Zhao M, Zhang G, Wang W (2019). Population genomic analysis and de novo assembly reveal the origin of weedy rice as an evolutionary game. Mol Plant.

[41] Wang L, Zhao L, Zhang X, Zhang Q, Jia Y, Wang G (2019). Large-scale identification and functional analysis of NLR genes in blast resistance in the Tetep rice genome sequence. Proc Natl Acad Sci.

[42] Ma X, Fan J, Wu Y, Zhao S, Zheng X, Sun C (2020). Whole-genome de novo assemblies reveal extensive structural variations and dynamic organelle-to-nucleus DNA transfers in African and Asian rice. Plant J.

[43] Xie X, Du H, Tang H, Tang J, Tan X, Liu W (2021). A chromosome-level genome assembly of the wild rice Oryza rufipogon facilitates tracing the origins of Asian cultivated rice. Science China Life Sciences.

[44] Choi JY, Lye ZN, Groen SC, Dai X, Rughani P, Zaaijer S (2020). Nanopore sequencing-based genome assembly and evolutionary genomics of circum-basmati rice. Genome Biol.

[45] Langmead B, Salzberg SL (2012). Fast gapped-read alignment with bowtie 2. Nat Methods.

[46] Ou S, Jiang N (2018). LTR_retriever: a highly accurate and sensitive program for identification of long terminal repeat retrotransposons. Plant Physiol.

[47] Simão FA, Waterhouse RM, Ioannidis P, Kriventseva EV, Zdobnov EM (2015). BUSCO: assessing genome assembly and annotation completeness with single-copy orthologs. Bioinformatics.

[48] Rhie A, Walenz BP, Koren S, Phillippy AM (2020). Merqury: reference-free quality, completeness, and phasing assessment for genome assemblies. Genome Biol.

[49] Marçais G, Delcher AL, Phillippy AM, Coston R, Salzberg SL, Zimin A (2018). MUMmer4: a fast and versatile genome alignment system. Plos Comput Biol.

[50] Price MN, Dehal PS, Arkin AP (2009). FastTree: computing large minimum evolution trees with profiles instead of a distance matrix. Mol Biol Evol.

[51] Wu D, Qiu J, Sun J, Song B, Olsen KM, Fan L (2022). Weedy rice, a hidden gold mine in the paddy field. Mol Plant.

[52] Chang CC, Chow CC, Tellier LC, Vattikuti S, Purcell SM, Lee JJ (2015). Second-generation PLINK: rising to the challenge of larger and richer datasets. GigaScience.

[53] Stanke M, Keller O, Gunduz I, Hayes A, Waack S, Morgenstern B (2006). AUGUSTUS: ab initio prediction of alternative transcripts. Nucleic Acids Res.

[54] Salamov AA, Solovyev VV (2000). Ab initio gene finding in Drosophila genomic DNA. Genome Res.

[55] Haas BJ, Salzberg SL, Zhu W, Pertea M, Allen JE, Orvis J (2008). Automated eukaryotic gene structure annotation using EVidenceModeler and the program to assemble spliced alignments. Genome Biol.

[56] Jones P, Binns D, Chang HY, Fraser M, Li W, McAnulla C (2014). Interproscan 5: genome-scale protein function classification. Bioinformatics.

[57] Emms DM, Kelly S (2019). Orthofinder: phylogenetic orthology inference for comparative genomics. Genome Biol.

[58] Buchfink B, Reuter K, Drost H (2021). Sensitive protein alignments at tree-of-life scale using diamond. Nat Methods.

[59] Haas BJ, Delcher AL, Wortman JR, Salzberg SL (2004). DAGchainer: a tool for mining segmental genome duplications and synteny. Bioinformatics.

[60] Zheng X, Pang H, Wang J, Yao X, Song Y, Li F (2022). Genomic signatures of domestication and adaptation during geographical expansions of rice cultivation. Plant Biotechnol J.

[61] Nguyen L, Schmidt HA, von Haeseler A, Minh BQ (2015). IQ-TREE: a fast and effective stochastic algorithm for estimating maximum-likelihood phylogenies. Mol Biol Evol.

[62] Green RE, Krause J, Briggs AW, Maricic T, Stenzel U, Kircher M (2010). A draft sequence of the Neandertal genome. Science.

[63] Martin SH, Davey JW, Jiggins CD (2015). Evaluating the use of ABBA-BABA statistics to locate introgressed loci. Mol Biol Evol.

[64] Ge SX, Jung D, Yao R (2020). ShinyGO: a graphical gene-set enrichment tool for animals and plants. Bioinformatics.

[65] Jayakodi M, Padmarasu S, Haberer G, Bonthala VS, Gundlach H, Monat C (2020). The barley pan-genome reveals the hidden legacy of mutation breeding. Nature.

[66] Nattestad M, Schatz MC (2016). Assemblytics: a web analytics tool for the detection of variants from an assembly. Bioinformatics.

[67] Wu D, Xie L, Sun Y, Huang Y, Jia L, Dong C, et al., A syntelog-based pan-genome provides insights into rice domestication and de-domestication. Datasets. Genome Sequence Archive. 2023. https://ngdc.cncb.ac.cn/bioproject/browse/PRJCA012143. 10.1186/s13059-023-03017-5 PMC10401782 37537691

[68] Wu D, Xie L, Sun Y, Huang Y, Jia L, Dong C, et al., A syntelog-based pan-genome provides insights into rice domestication and de-domestication. Datasets. Genome Sequence Archive. 2023. https://ngdc.cncb.ac.cn/bioproject/browse/PRJCA012309. 10.1186/s13059-023-03017-5 PMC10401782 37537691

[69] Zheng X, Pang H, Wang J, Yao X, Song Y, Li F, et al. Genomic signatures of domestication and adaptation during geographical expansions of rice cultivation. Datasets. European Nucleotide Archive. 2023. https://www.ebi.ac.uk/ena/browser/view/PRJNA657701. 10.1111/pbi.13730 PMC8710896 34664353

[70] Wu D, Xie L, Sun Y, Huang Y, Jia L, Dong C, et al. A syntelog-based pan-genome provides insights into rice domestication and de-domestication. 2023. Zenodo Code. 10.5281/zenodo.7196576. 10.1186/s13059-023-03017-5 PMC10401782 37537691

[71] Wu D, Xie L, Sun Y, Huang Y, Jia L, Dong C, et al., A syntelog-based pan-genome provides insights into rice domestication and de-domestication. Datasets. Genome Variation Map. 2023. https://ngdc.cncb.ac.cn/bioproject/browse/PRJCA018336. 10.1186/s13059-023-03017-5 PMC10401782 37537691

[72] Wu D, Xie L, Sun Y, Huang Y, Jia L, Dong C, et al. A syntelog-based pan-genome provides insights into rice domestication and de-domestication. 2023. Zenodo. 10.5281/zenodo.7248110. 10.1186/s13059-023-03017-5 PMC10401782 37537691

[73] Wu D, Xie L, Sun Y, Huang Y, Jia L, Dong C, et al., A syntelog-based pan-genome provides insights into rice domestication and de-domestication. Github. 2023. https://github.com/dongyawu/PangenomeEvolution. 10.1186/s13059-023-03017-5 PMC10401782 37537691

[74] Wu D, Xie L, Sun Y, Huang Y, Jia L, Dong C (2023). A syntelog based pan genome provides insights into rice domestication and de domestication. Zenodo..

