# Supplementary material for: A syntelog-based pan-genome provides insights into rice domestication and de-domestication
Source: Genome Biol. 2023 Aug 3;24:179. doi: 10.1186/s13059-023-03017-5 (PMC10401782; doi:10.1186/s13059-023-03017-5)
Supplement: Supplementary file 1 — Additional file 1: Fig. S1. Hi-C interaction heatmaps for each chromosome from four rice accessions. Fig. S2. Statistical information of the rice genomes used in this study. Fig. S3. Dot plots of newly generated rice assemblies in this study against the reference assembly Nipponbare (IRGSP) and gapless assembly MH63RS3. Fig. S4. Assembly quality assessment in base accuracy. Fig. S5. PCA reveals the representativeness and diversity of genome assemblies used in this study. Fig. S6. Performance of BLASTP and Diamond (under different modes) in syntelog identification. Fig. S7. Pairwise synteny reveals evolutionary signatures in groups and individuals. Fig. S8. Comparison of MCL and synteny-based clustering. Fig. S9. Benchmarking analysis on the influences of inflation parameters in the MCL clustering in rice genomes. Fig. S10. Composition and features of the rice syntelog-based pangenome. Fig. S11. Comparison of NLR genes in rice genomes from different ecotypes and subspecies. Fig. S12. Genomic features of NLRs in rice genomes. Fig. S13. Frequency differences in gene presence between GJ and XI along chromosome 7. Fig. S14. Haplotype analysis on rice syntelogs. (a) Definition of haplotype diversity and divergence. Fig. S15. Ancestral haplotype landscape on chromosome 1 to chromosome 12. Fig. S16. Synonymous substitution rates (Ks) of genes in putative introgression blocks and their neighboring left and right regions. Fig. S17. Population structure of wild rice accessions used in this study. Fig. S18. Introgression fd distributions of ABBA-BABA test on chromosome 1 to chromosome 12. Fig. S19. Comparison of fd using all genomes and landraces only under different topologies on chromosome 1. Fig. S20. Summary of structural variations between weedy and cultivated rice genomes. Fig. S21. Structural variations (>50 bp) between assemblies of cultivar accession NJ11 and weedy rice accession CX20 on 12 chromosomes. Fig. S22. The Integrative Genomics Viewer (IGV) snapshots show the [file 13059_2023_3017_MOESM1_ESM.pdf]

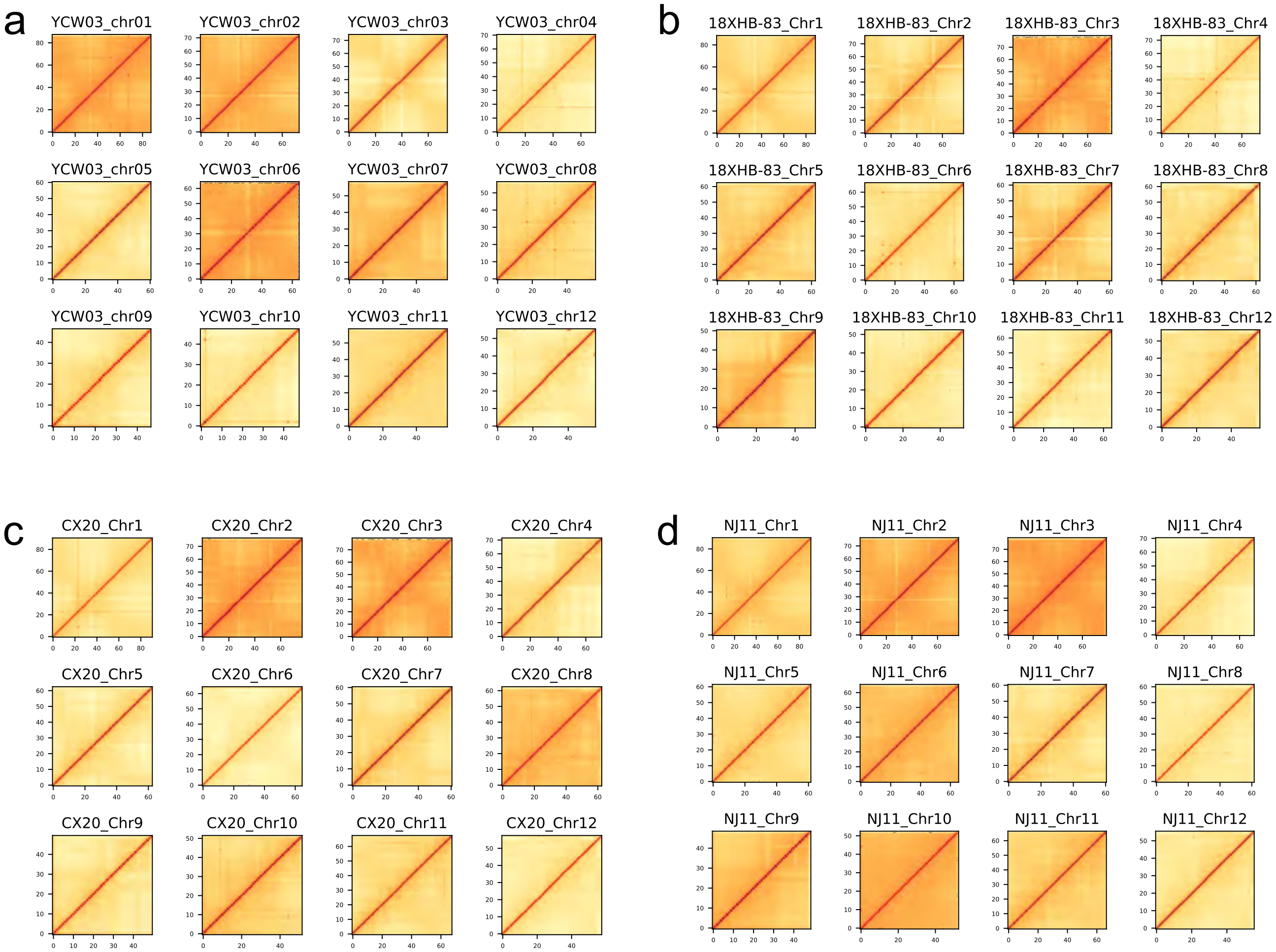

Fig. S1 Hi-C interaction heat-maps for each chromosome of four rice accessions (**a**, YCW03; **b**, 18XHB-83; **c**, CX20; **d**, NJ11).

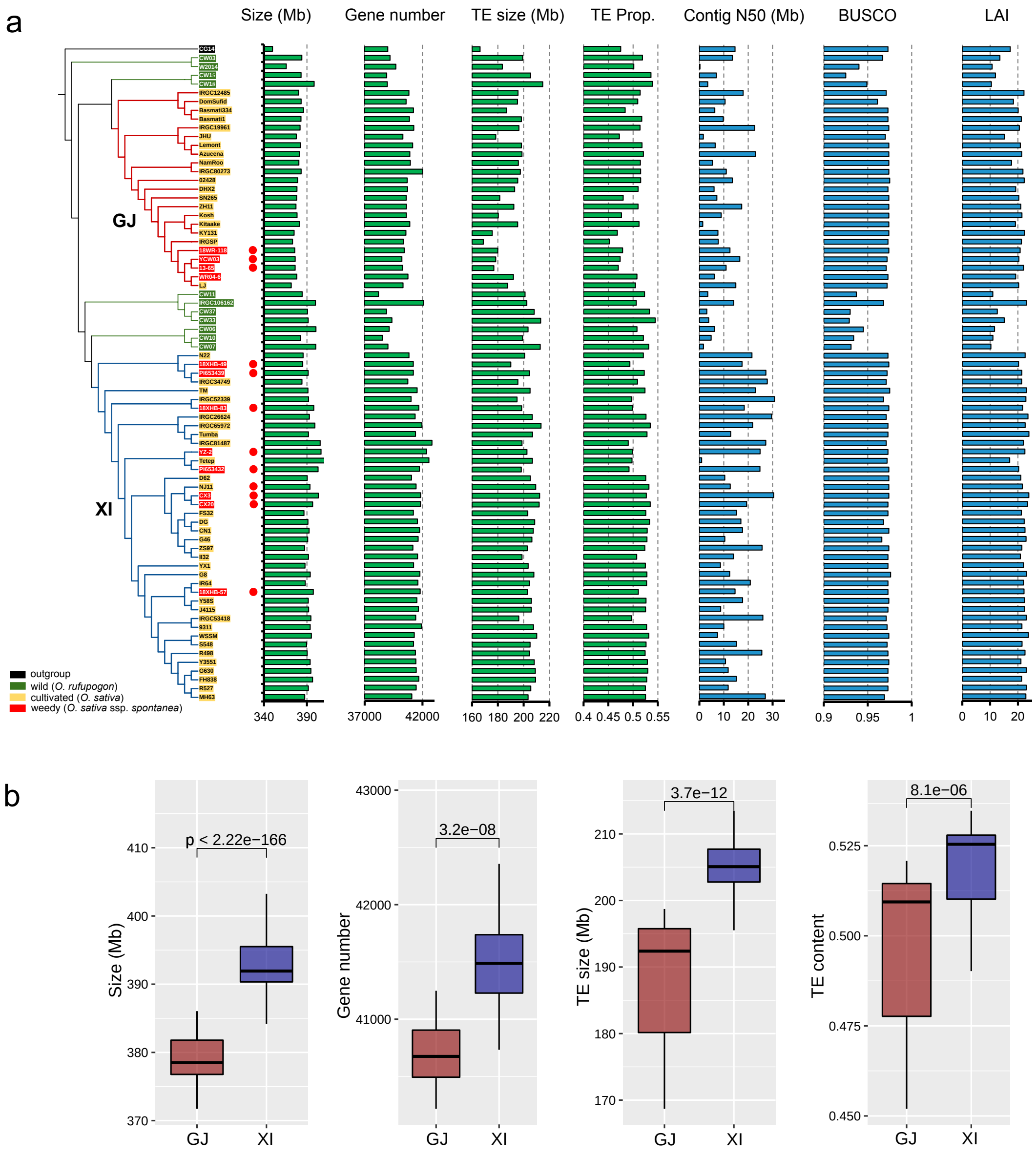

Fig. S2 Statistical information of rice genomes used in this study. (**a**) assembly (size), annotation (gene number, TE size and proportion) and quality assessment (Contig N50, BUSCO and LAI) of rice genomes. (**b**) Differences in genomic features between subspecies GJ and XI. In the boxplots, the horizontal line shows the median value, and the whiskers show the 25% and 75% quartile values of each genomic feature. *P* values are calculated by Student's *t* test.

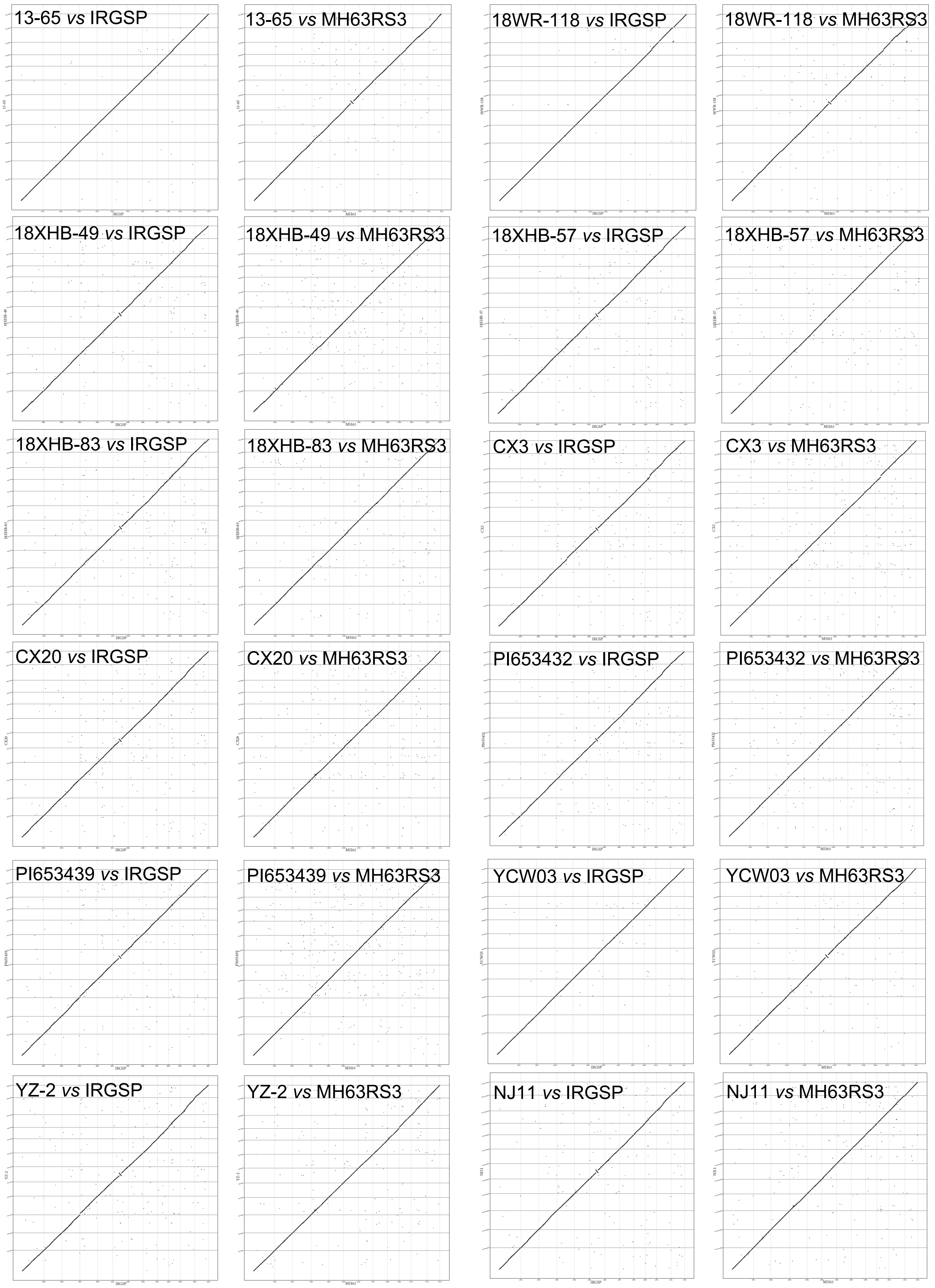

Fig. S3 Dot plots of newly generated rice assemblies in this study against reference assembly Nipponbare (IRGSP) and gapless assembly MH63RS3.

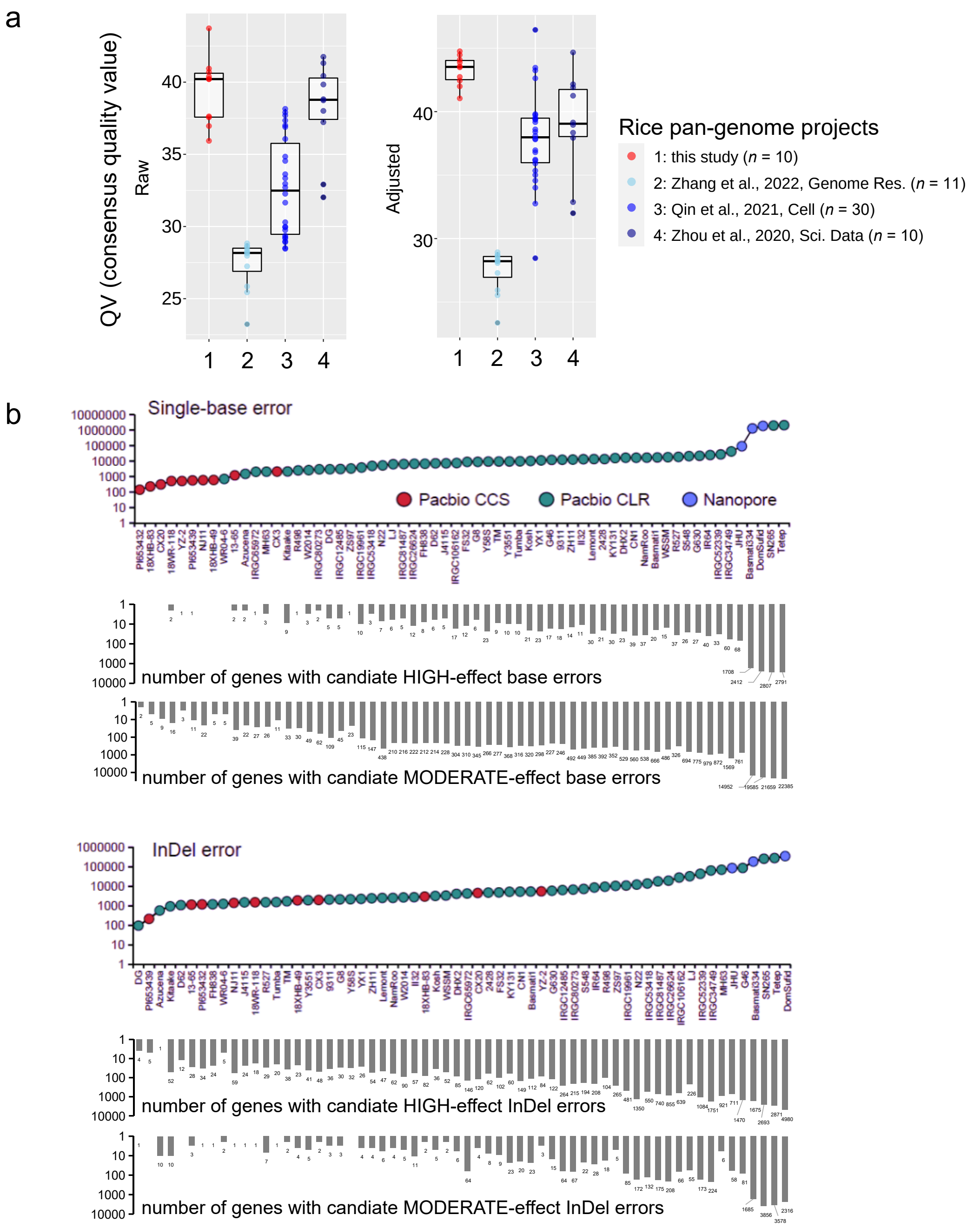

Fig. S4 Assembly quality assessment in base accuracy. (**a**) QVs for rice genomes in four rice pan-genome projects, using yak (https://github.com/lh3/yak). In the boxplots, the horizontal line shows the median value, and the whiskers show the 25% and 75% quartile values of QVs. (**b**) The number and annotation to SNVs and InDels for each genome by mapping NGS short reads against itself assembly.

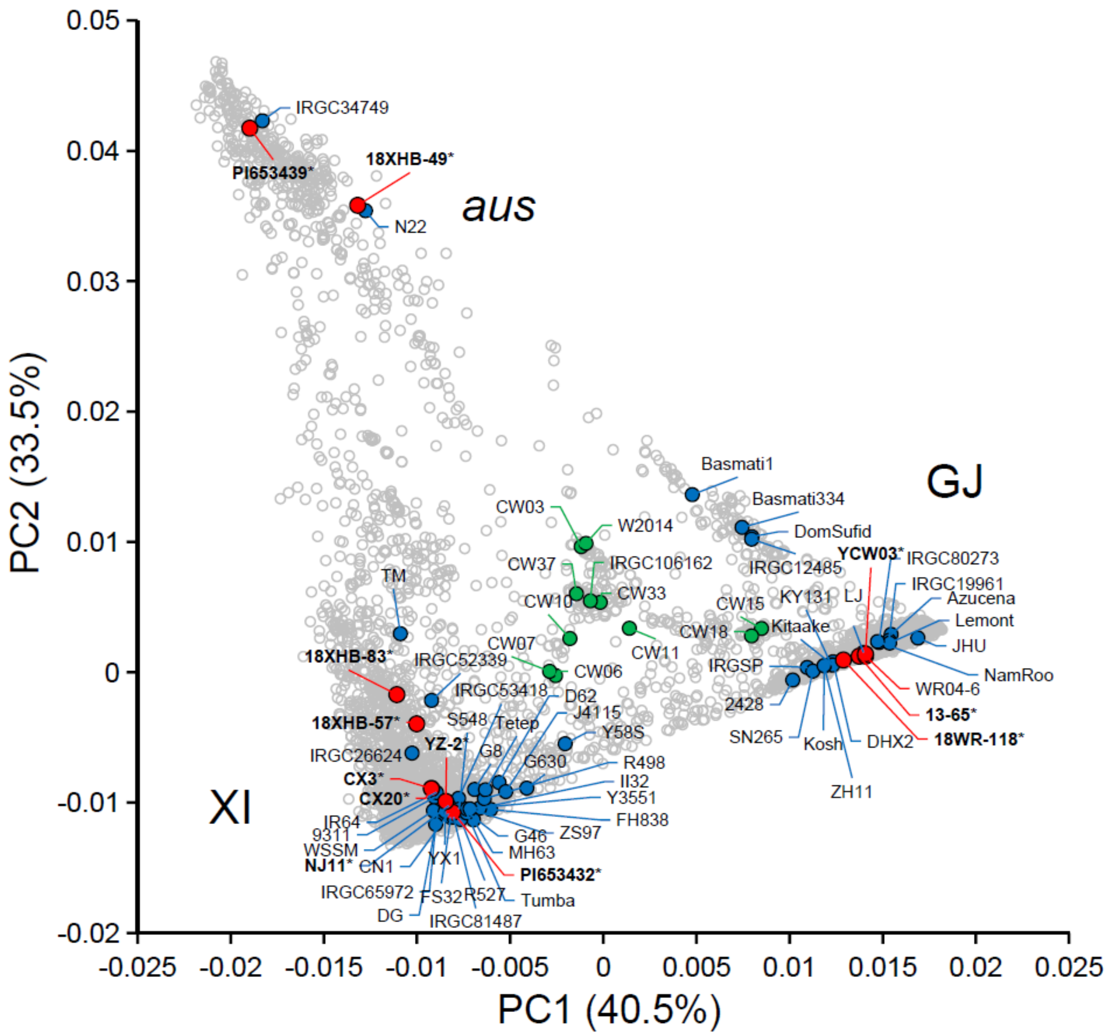

Fig. S5 PCA analysis reveals the representativeness and diversity of genome assemblies used in this study. The first two principle components are shown. Filled circles indicate the assemblies used in this study. Green, blue and red represent wild, cultivated and weedy assemblies, respectively.

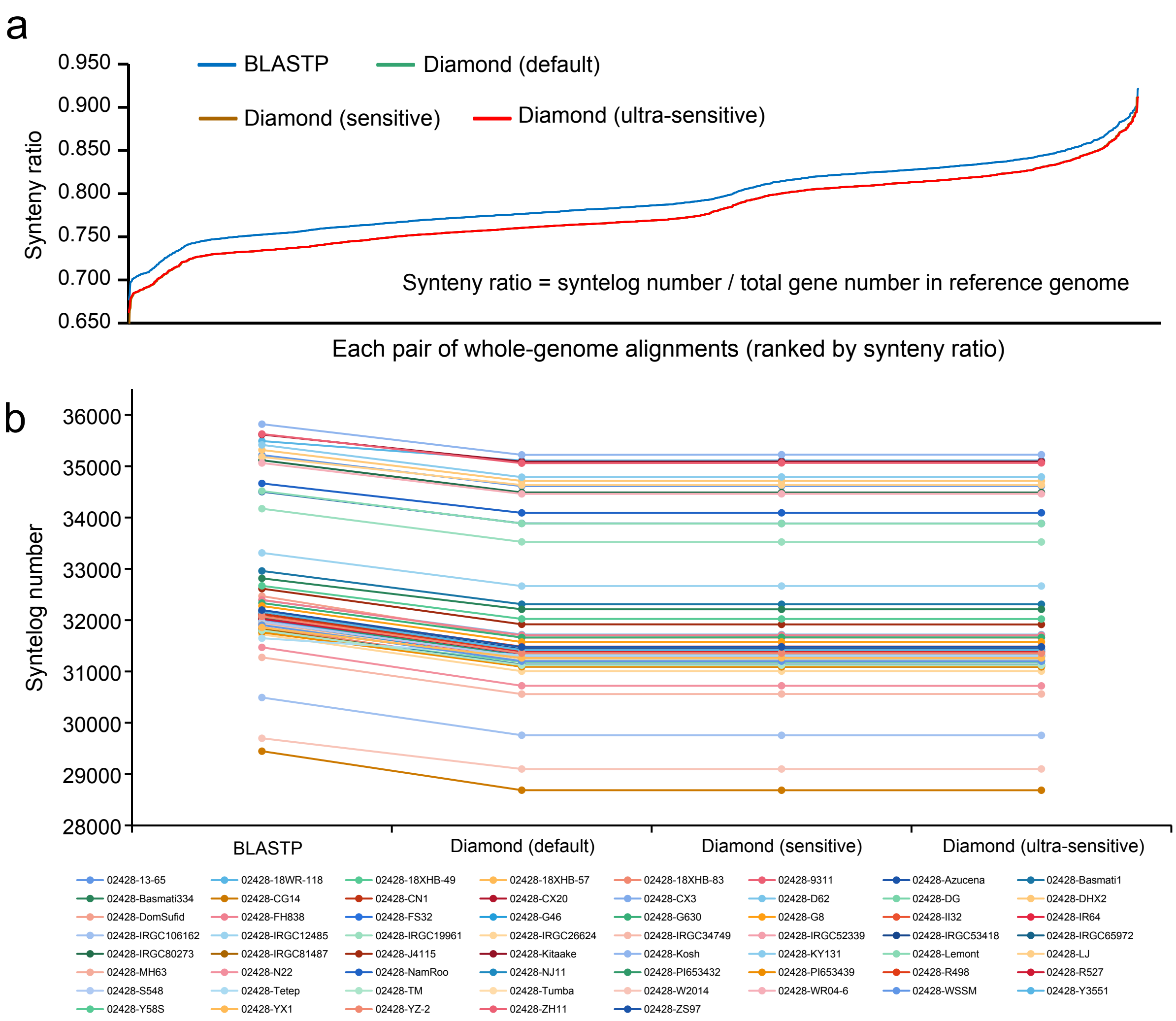

Fig. S6 Performance of BLASTP and Diamond (under different modes) in syntelog identification. (**a**) BLASTP approach identifies more syntelogs than Diamond. (**b**) Syntelog number between accession 02428 with other accessions identified using BLASTP and Diamond under different modes.

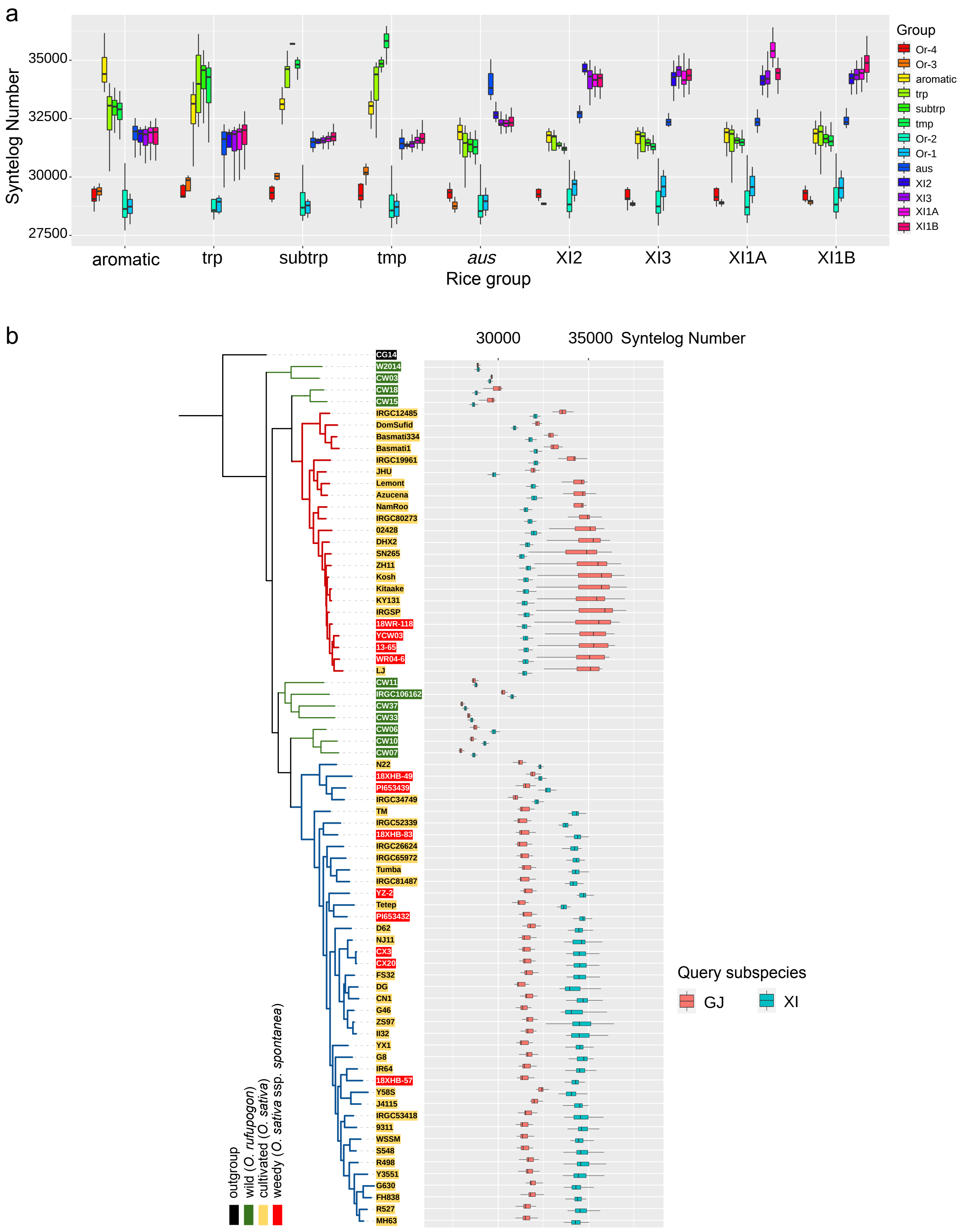

Fig. S7 Pairwise synteny reveals evolutionary signatures in groups and individuals. (**a**) Syntelog numbers between rice groups. (**b**) Syntelog numbers between each accession to other accessions from GJ and XI (including *aus*) subspecies. In the boxplots, the horizontal line shows the median value, and the whiskers show the 25% and 75% quartile values of syntelog numbers.

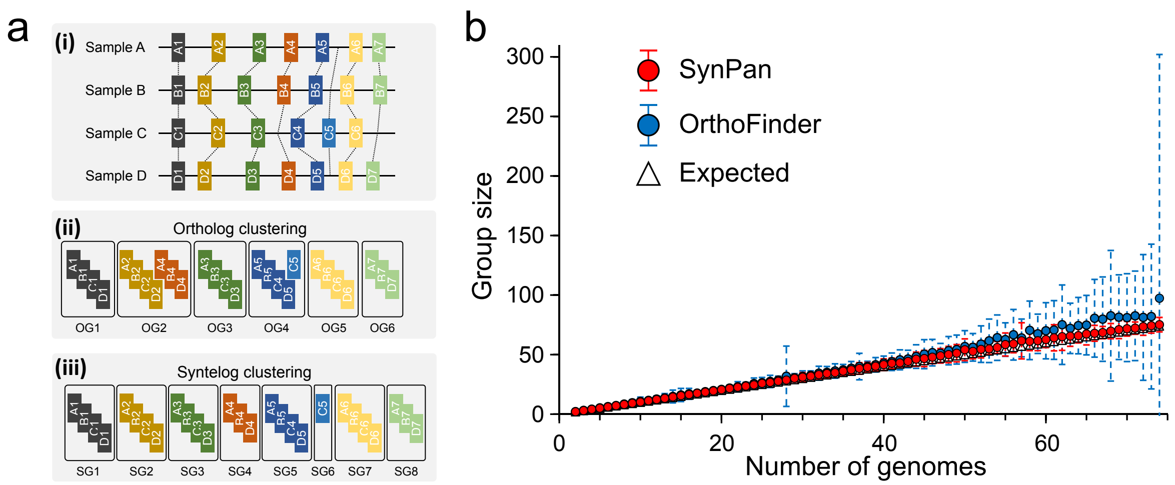

Fig. S8 Comparison between MCL and synteny-based clustering. (**a**) A brief scheme illustrating MCL ortholog clustering and syntelog clustering. (**b**) Group size comparison using synteny-based clustering (SynPan) and MCL clustering (OrthoFinder).

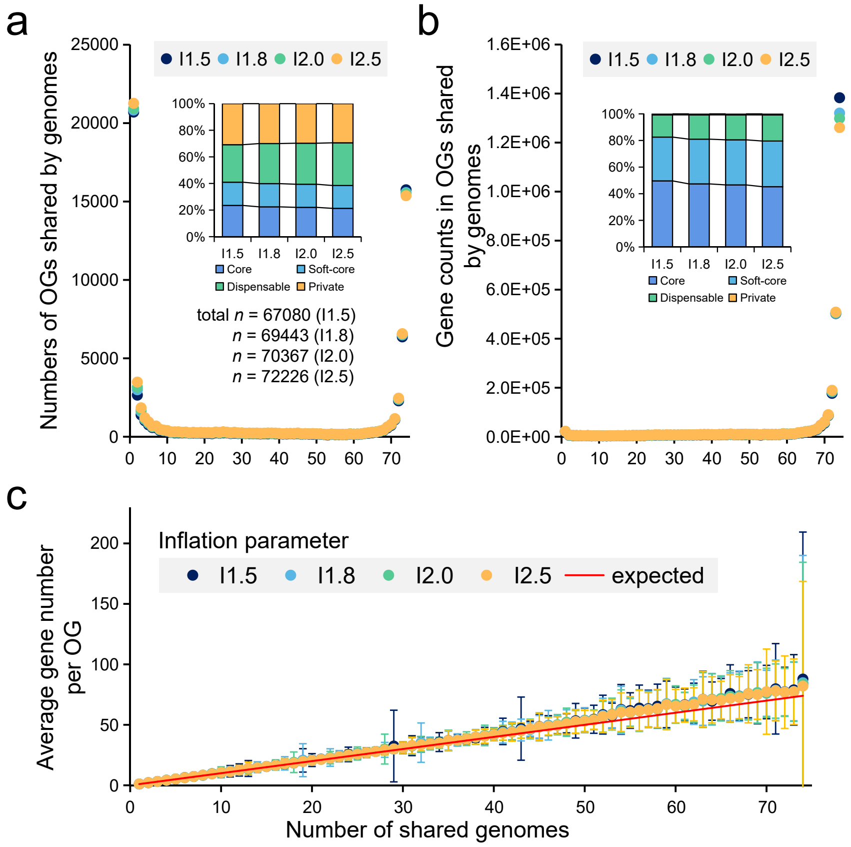

Fig. S9 Benchmarking analysis on the influences of inflation parameters in the MCL clustering in rice genomes. (**a**) The OG numbers shared by different sizes of rice genomes under different inflation parameters. (**b**) Gene counts in OGs shared by different sizes of genomes. (**c**) Average gene number per OG under different inflation parameters

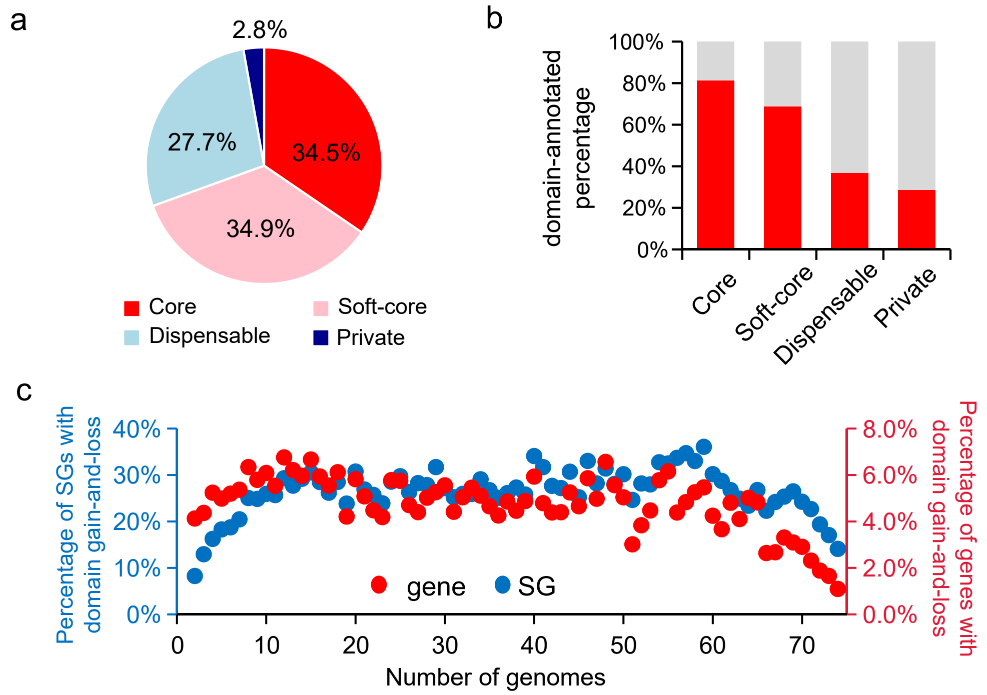

Fig. S10 Composition and features of rice syntelog-based pan-genome. (**a**) Pan-gene composition (core, soft-core, dispensable and private) of rice pan-genome. (**b**) Proportions of domain annotated genes in four categories of rice pan-genome. (**c**) Percentage of SGs (blue) and genes (red) with domain gain-and-loss.

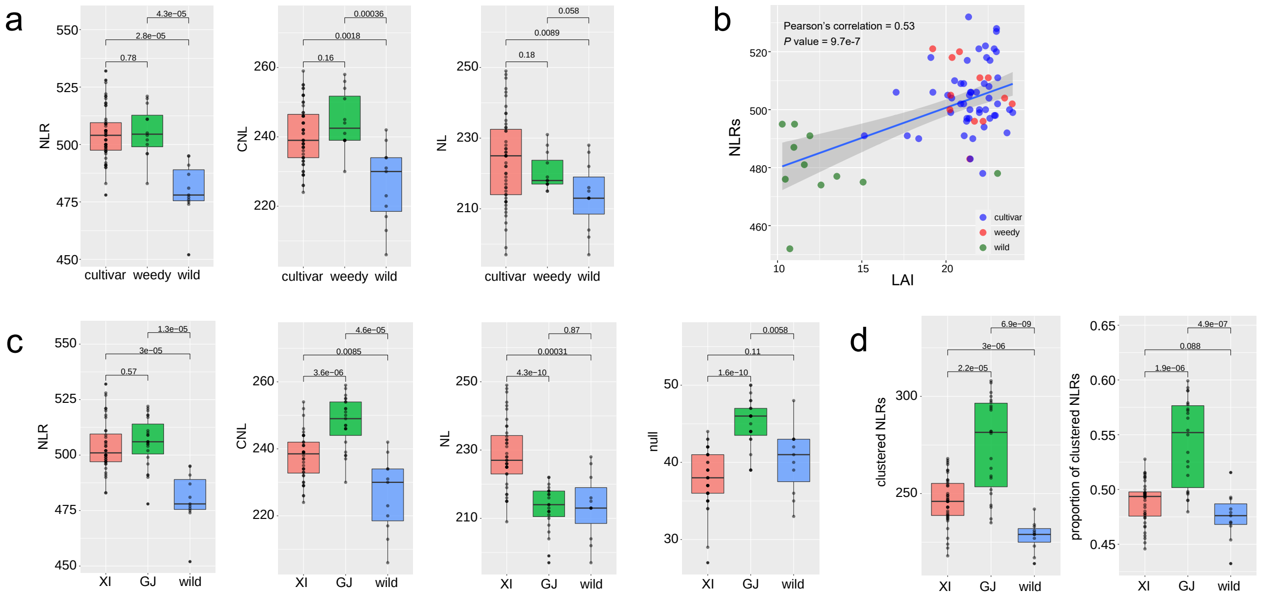

Fig. S11 Comparison of NLR genes in rice genomes from different ecotypes and subspecies. (a) Distribution of different NLR genes in wild, cultivated and weedy accessions. (b) Relationship between genome assembly completeness (as indicated by LAI) and NLR size. In the boxplots, the horizontal line shows the median value, and the whiskers show the 25% and 75% quartile values of NLR sizes. (c) Distribution of different NLR genes in wild rice, XI and GJ. (d) Clustered NLRs in wild rice, XI and GJ. P values are calculated using Wilcoxon test.

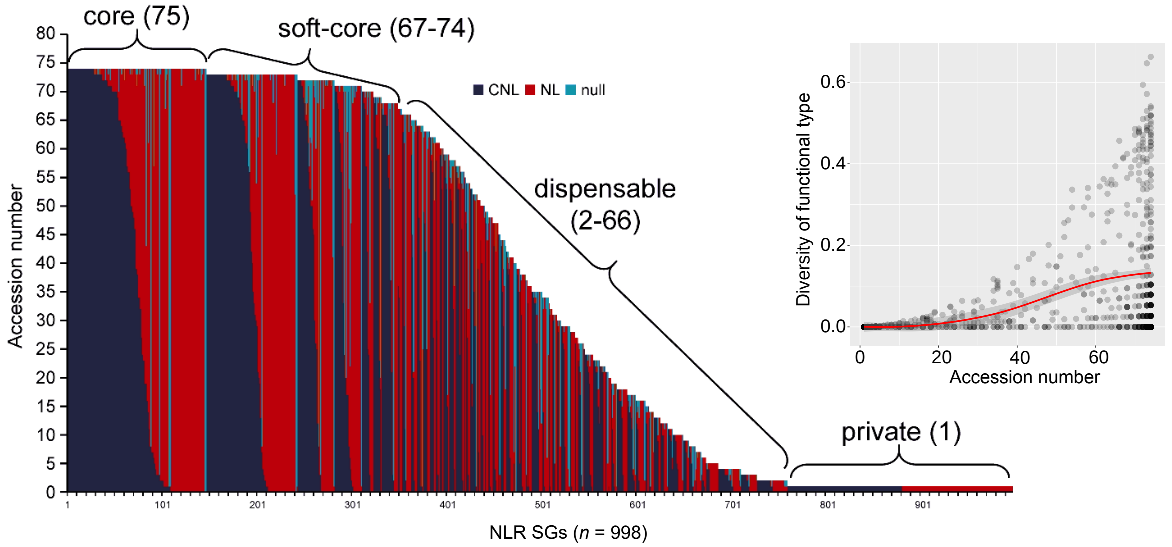

Fig. S12 Dynamics of domain architectures in the rice NLRome. CNL, NL and null (no canonical architectures) are defined as three NLR architecture types in rice NLRome. Multiple types are observed for most SGs.

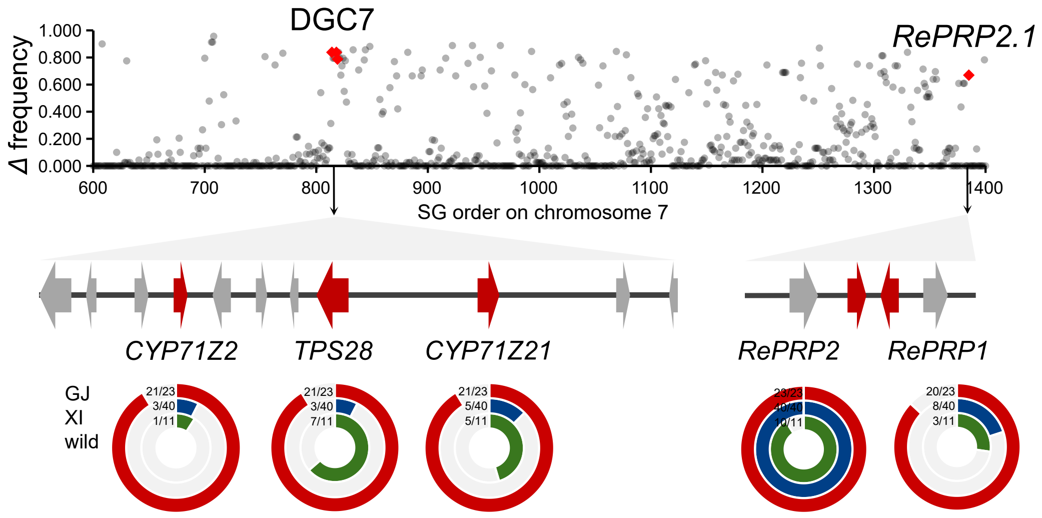

Fig. S13 Frequency differences in gene presence between GJ and XI along chromosome 7. Each dot represents one SG. The gene PAV profiling of the antimicrobial diterpenoid biosynthetic gene cluster DGC7 and tandem duplicates *RePRP2.1* and *RePRP2.2*, suppressors of root cell expansion, is shown.

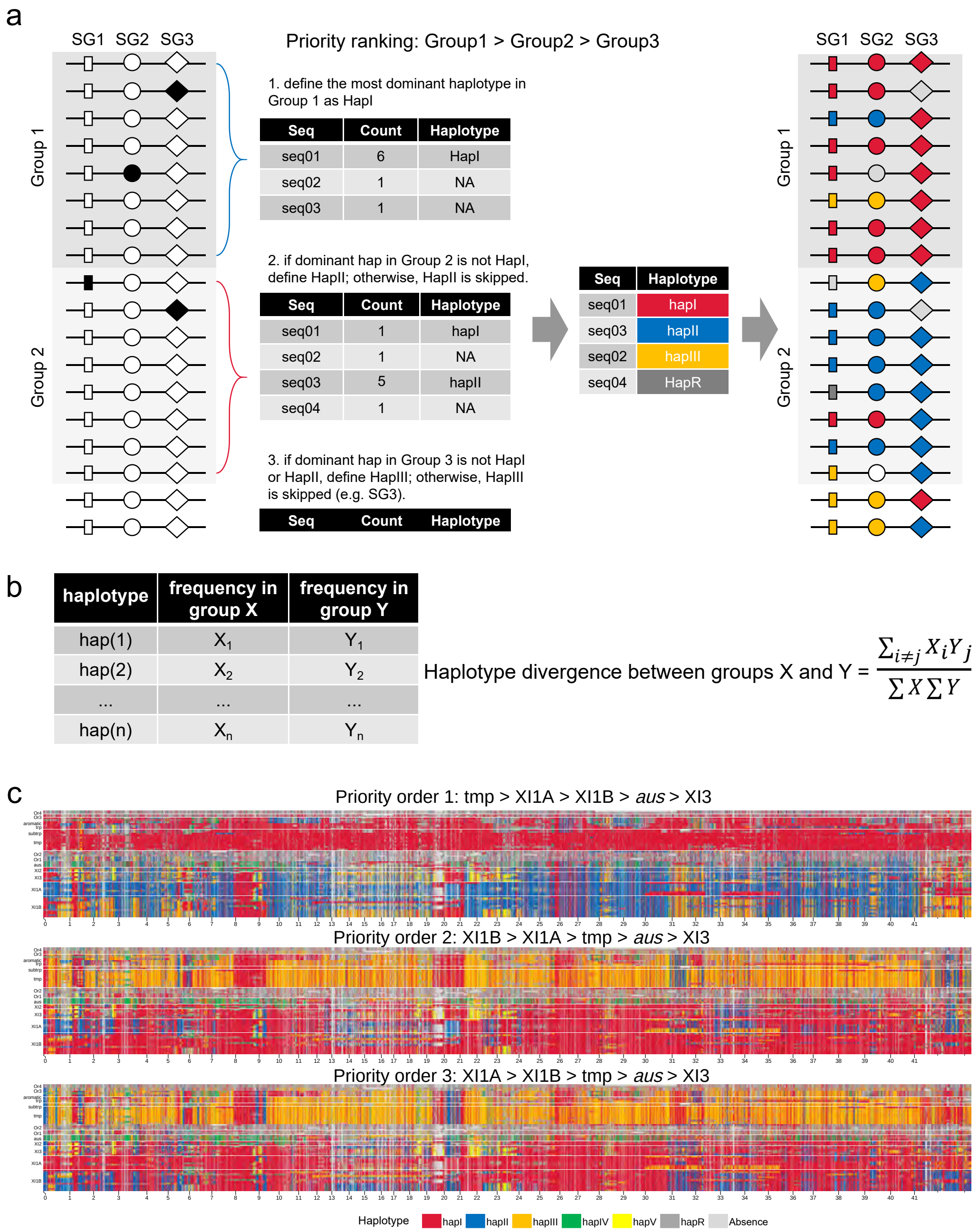

Seq	Count	Haplotype
seq01	6	HapI
seq02	1	NA
seq03	1	NA

Seq	Count	Haplotype
seq01	1	hapI
seq02	1	NA
seq03	5	hapII
seq04	1	NA

Seq	Count	Haplotype

Seq	Haplotype
seq01	hapI
seq03	hapII
seq02	hapIII
seq04	HapR

haplotype	frequency in group X	frequency in group Y
hap(1)	X_1	Y_1
hap(2)	X_2	Y_2
...	...	...
hap(n)	X_n	Y_n

Haplotype divergence between groups X and Y = $\frac{\sum_{i \neq j} X_i Y_j}{\sum X \sum Y}$

Fig. S14 Haplotype analysis on rice syntelogs. (**a**) Definition of haplotype diversity and divergence. Haplotype diversity and divergence represent average haplotype differences among sequences in a syntelog group within one group and among two groups, respectively, where Xi and Xj are the presence count of haplotype i and haplotype j in group X, and ∑X is the total sequence count within a syntelog group. (**b**) Brief scheme illustrating the assignment and visualization of ancestral haplotypes for each genome. A group priority-based referring strategy is used to assign ancestral haplotypes. Group information is prior based on whole-genome phylogeny or population structure. The most dominant sequence in syntelogs from Group 1 is set as hapI. If the most dominant sequence from Group 2 is not HapI, then define HapII, otherwise HapII is skipped. If the most dominant sequence from Group 3 is neither HapI nor HapII, then define HapIII, otherwise HapIII is skipped. By analogy, dominant haplotypes are determined and colored for each syntelog group. Rare haplotypes are named as HapR colored by dark gray. In this study, the group priority order is set as tmp > XI1A > XI1B > *aus* > XI3. Different orders have no influences on the calculation of haplotype diversity and divergence. (**c**) Mosaic graphs of ancestral haplotypes on chromosome 1 across 74 rice genomes with different priority orders: tmp > XI1A > XI1B > *aus* > XI3, XI1B > XI1A > tmp > *aus* > XI3, and XI1A > XI1B > tmp > *aus* > XI3. For each window, the same color indicates the same haplotype, and dark and light gray indicate rare haplotypes and syntelog absence, respectively.

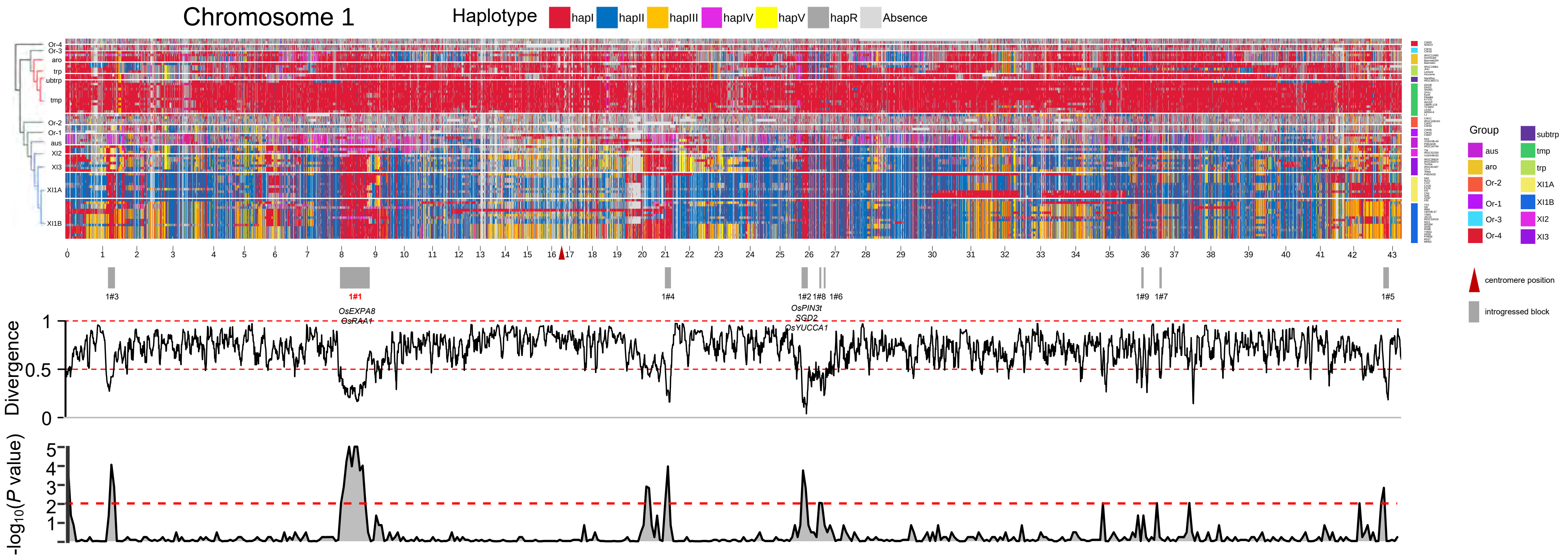

Chromosome 1
Haplotype hapI hapII hapIII hapIV hapV hapR Absence
Or-4
Or-3
aro
trp
subtrp
tmp
Or-2
Or-1
aus
XI2
XI3
XI1A
XI1B
Group
aus aro Or-2 Or-1 Or-3 Or-4 subtrp tmp trp XI1A XI1B XI2 XI3
1#3
1#1
OsEXPA8
OsRAA1
1#4
1#2 1#8 1#6
OsPIN3t
SGD2
OsYUCCA1
1#9 1#7
1#5
centromere position
introgressed block
Divergence
-log10(P value)

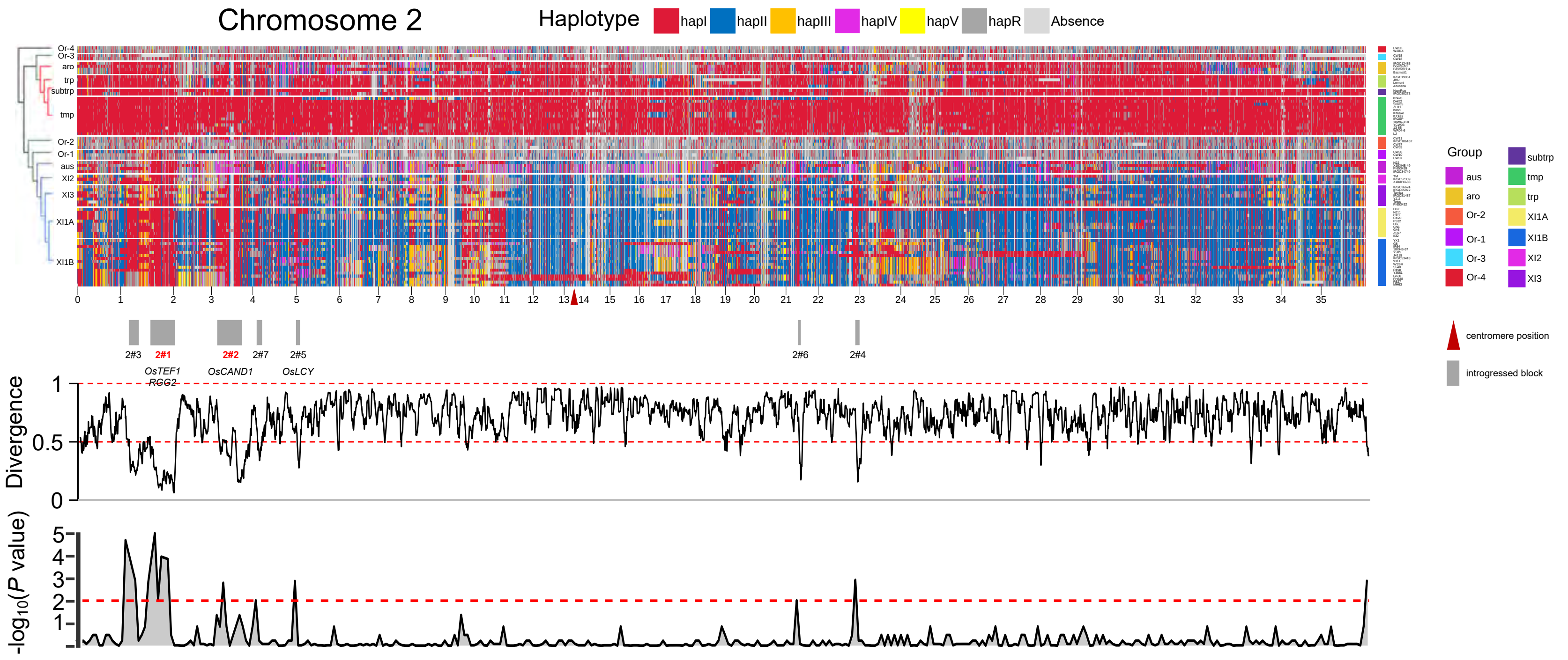

Chromosome 2
Haplotype hapI hapII hapIII hapIV hapV hapR Absence
Or-4
Or-3
aro
trp
subtrp
tmp
Or-2
Or-1
aus
XI2
XI3
XI1A
XI1B
Group
aus aro Or-2 Or-1 Or-3 Or-4 subtrp tmp trp XI1A XI1B XI2 XI3
2#3
2#1
OsTEF1
RGG2
2#2
OsCAND1
2#7
2#5
OsLCY
2#6
2#4
centromere position
introgressed block
Divergence
-log10(P value)

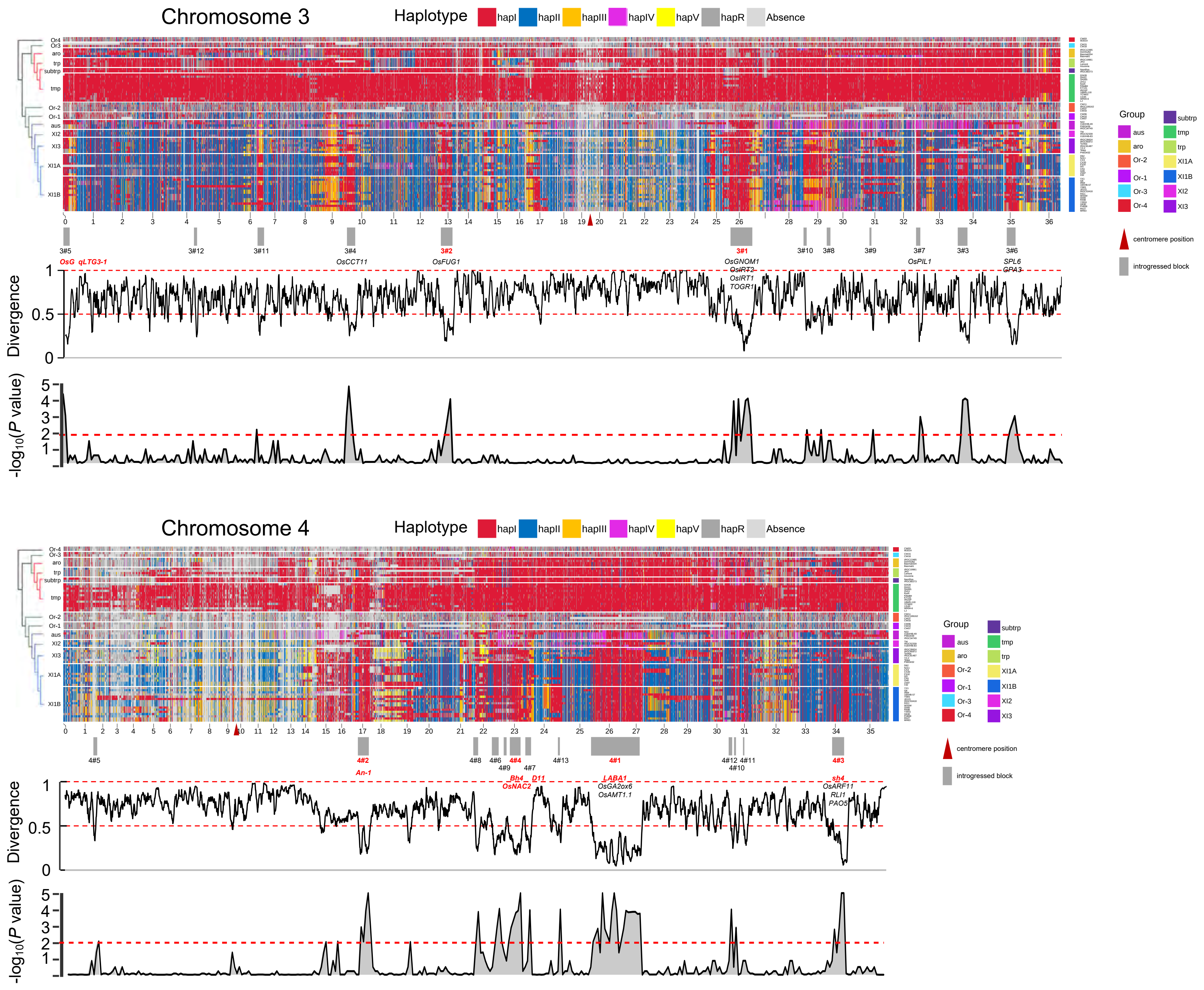

Chromosome 3
Haplotype
hapI
hapII
hapIII
hapIV
hapV
hapR
Absence
Or4
Or3
aro
trp
subtrp
tmp
Or-2
Or-1
aus
XI2
XI3
XI1A
XI1B
3#5
3#12
3#11
3#4
3#2
3#1
3#10
3#8
3#9
3#7
3#3
3#6
OsG qLTG3-1
OsCCT11
OsFUG1
OsGNOM1
OsIRT2
OsIRT1
TOGR1
OsPIL1
SPL6
GPA3
Divergence
-log10(P value)
Group
centromere position
introgressed block
Chromosome 4
Or-4
Or-3
4#5
4#2
4#8
4#6
4#9
4#4
4#7
4#13
4#1
4#12
4#11
4#10
4#3
An-1
Bh4
OsNAC2
D11
LABA1
OsGA2ox6
OsAMT1.1
sh4
OsARF11
RLI1
PAO5

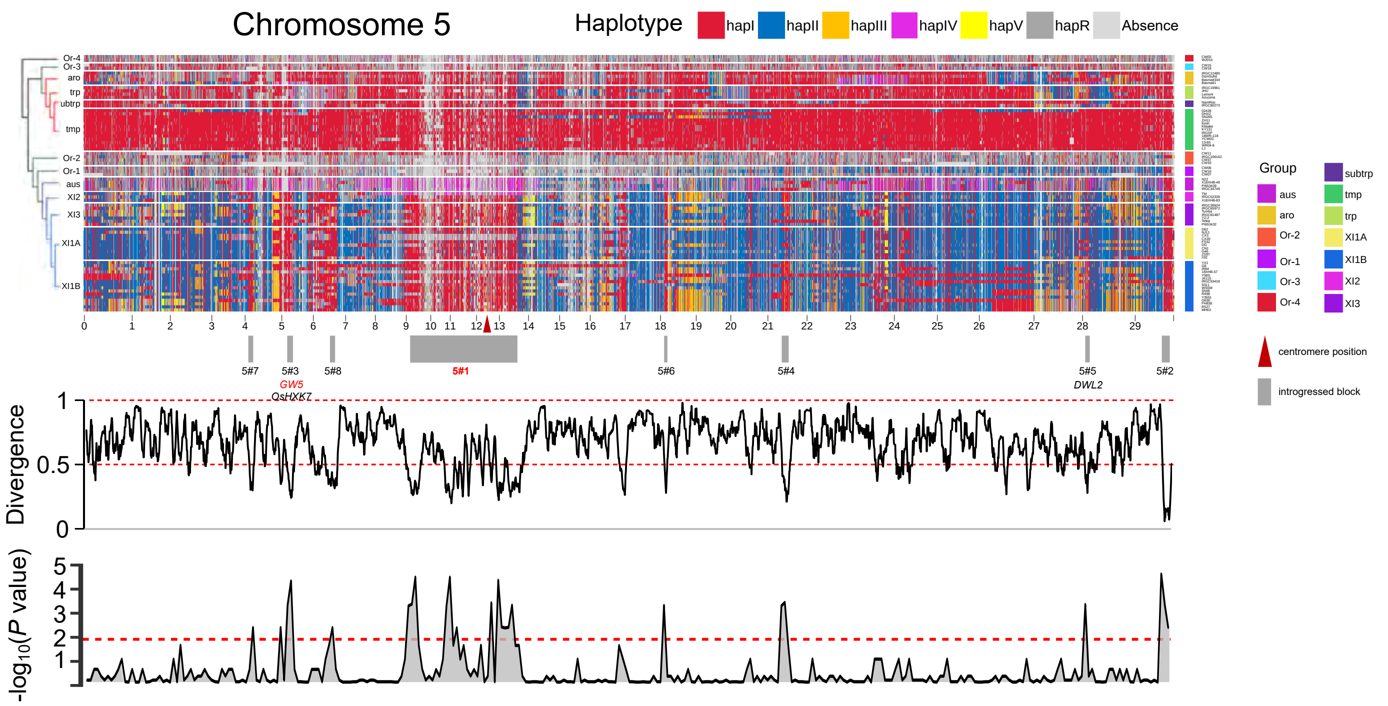
Chromosome 5
Haplotype
hapI
hapII
hapIII
hapIV
hapV
hapR
Absence
Or-4
Or-3
aro
trp
subtrp
tmp
Or-2
Or-1
aus
XI2
XI3
XI1A
XI1B
Group
centromere position
introgressed block
5#7
5#3
5#8
5#1
5#6
5#4
5#5
5#2
GW5
OsHXK7
DWL2
Divergence
-log10(P value)

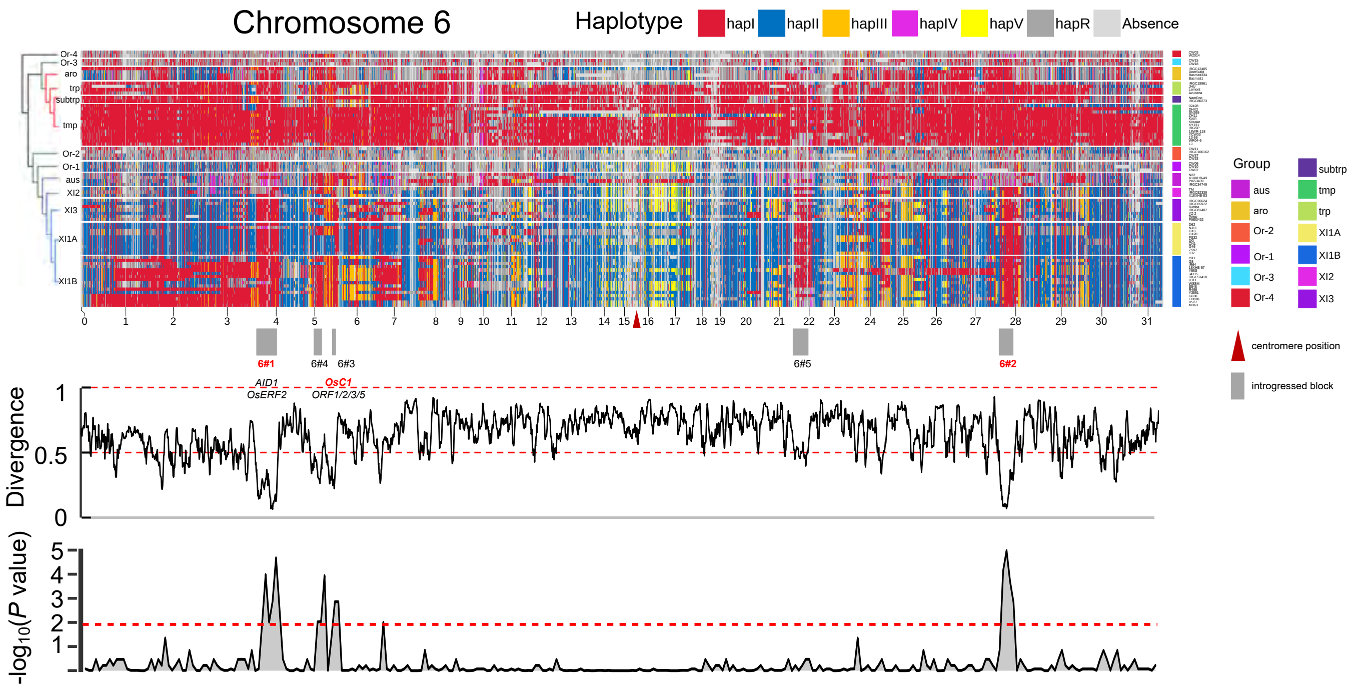
Chromosome 6
Haplotype
hapI
hapII
hapIII
hapIV
hapV
hapR
Absence
Or-4
Or-3
aro
trp
subtrp
tmp
Or-2
Or-1
aus
XI2
XI3
XI1A
XI1B
Group
centromere position
introgressed block
6#1
6#4
6#3
6#5
6#2
AID1
OsERF2
OsC1
ORF1/2/3/5
Divergence
-log10(P value)

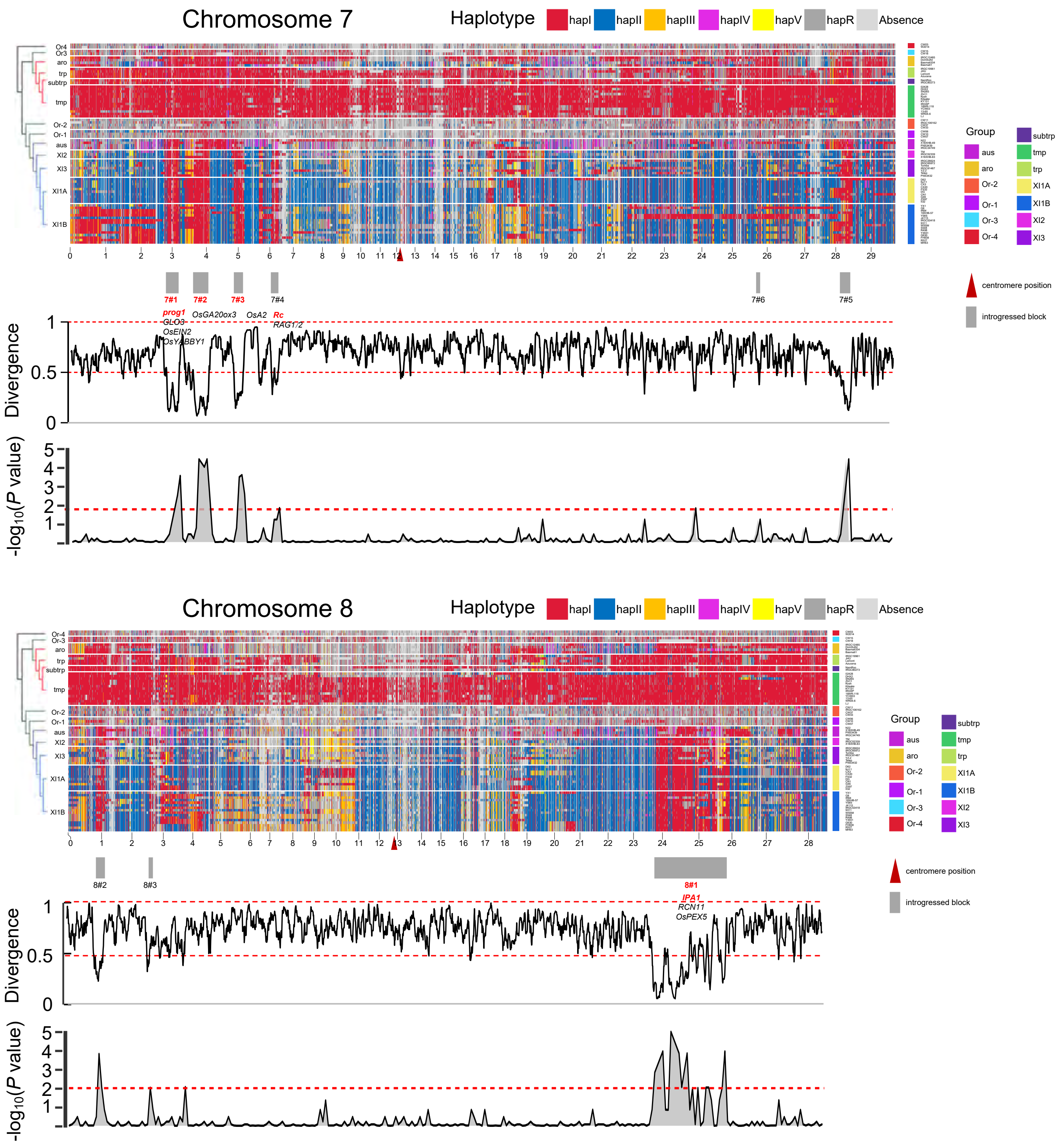

Chromosome 7
Haplotype
hapI
hapII
hapIII
hapIV
hapV
hapR
Absence
Or4
Or3
aro
trp
subtrp
tmp
Or-2
Or-1
aus
XI2
XI3
XI1A
XI1B
Group
aus
aro
Or-2
Or-1
Or-3
Or-4
subtrp
tmp
trp
XI1A
XI1B
XI2
XI3
centromere position
introgressed block
7#1
7#2
7#3
7#4
7#6
7#5
prog1
GLO3
OsEIN2
OsYABBY1
OsGA20ox3
OsA2
Rc
RAG1/2
Divergence
-log10(P value)
Chromosome 8
Haplotype
hapI
hapII
hapIII
hapIV
hapV
hapR
Absence
Or-4
Or-3
aro
trp
subtrp
tmp
Or-2
Or-1
aus
XI2
XI3
XI1A
XI1B
Group
aus
aro
Or-2
Or-1
Or-3
Or-4
subtrp
tmp
trp
XI1A
XI1B
XI2
XI3
centromere position
introgressed block
8#2
8#3
8#1
IPA1
RCN11
OsPEX5
Divergence
-log10(P value)

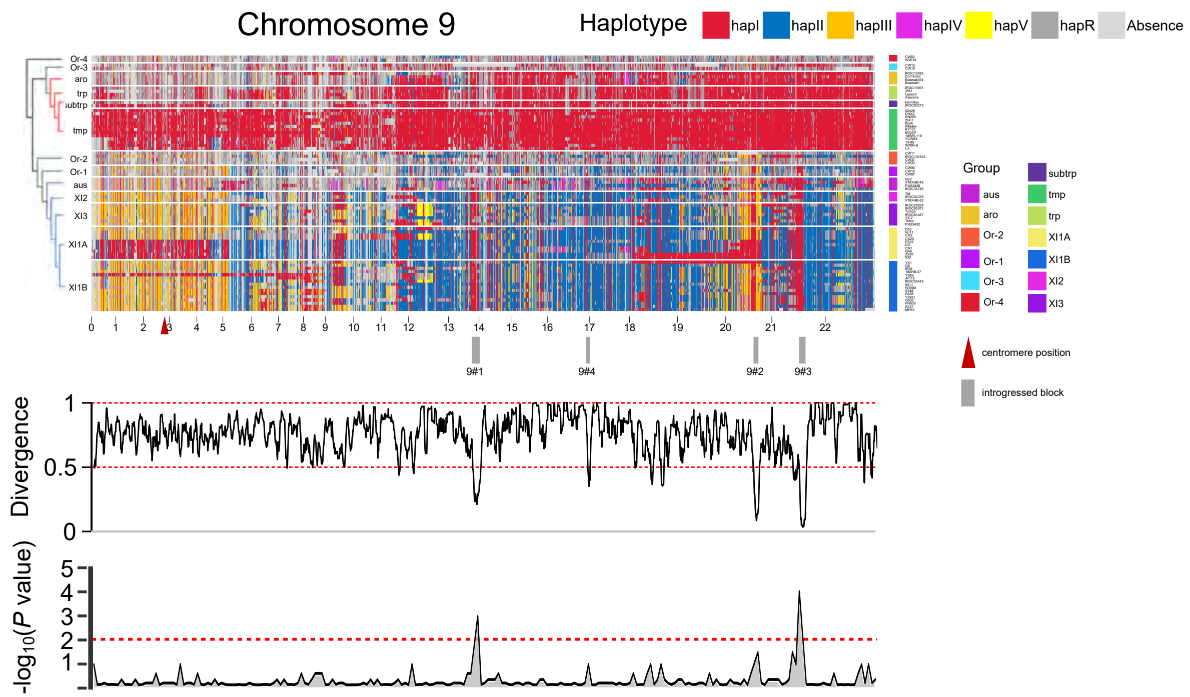
Chromosome 9
Haplotype
hapI
hapII
hapIII
hapIV
hapV
hapR
Absence
Or-4
Or-3
aro
trp
subtrp
tmp
Or-2
Or-1
aus
XI2
XI3
XI1A
XI1B
Group
aus
aro
Or-2
Or-1
Or-3
Or-4
subtrp
tmp
trp
XI1A
XI1B
XI2
XI3
centromere position
introgressed block
9#1
9#4
9#2
9#3
Divergence
-log10(P value)

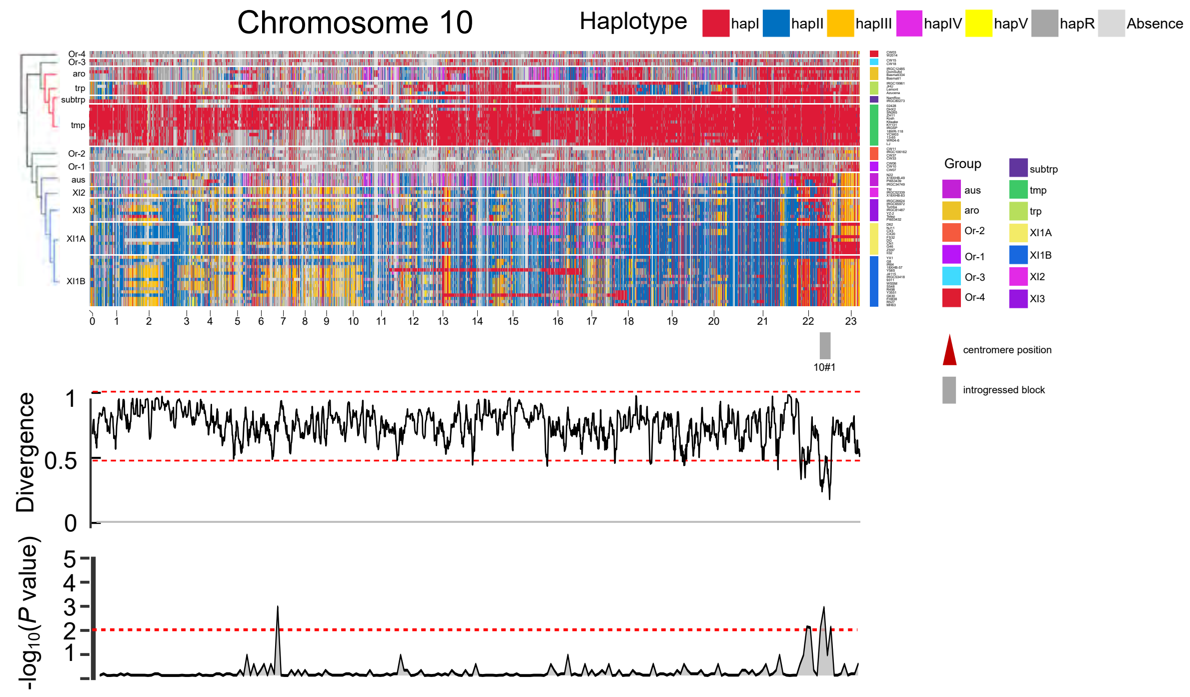
Chromosome 10
Haplotype
hapI
hapII
hapIII
hapIV
hapV
hapR
Absence
Or-4
Or-3
aro
trp
subtrp
tmp
Or-2
Or-1
aus
XI2
XI3
XI1A
XI1B
Group
aus
aro
Or-2
Or-1
Or-3
Or-4
subtrp
tmp
trp
XI1A
XI1B
XI2
XI3
centromere position
introgressed block
10#1
Divergence
-log10(P value)

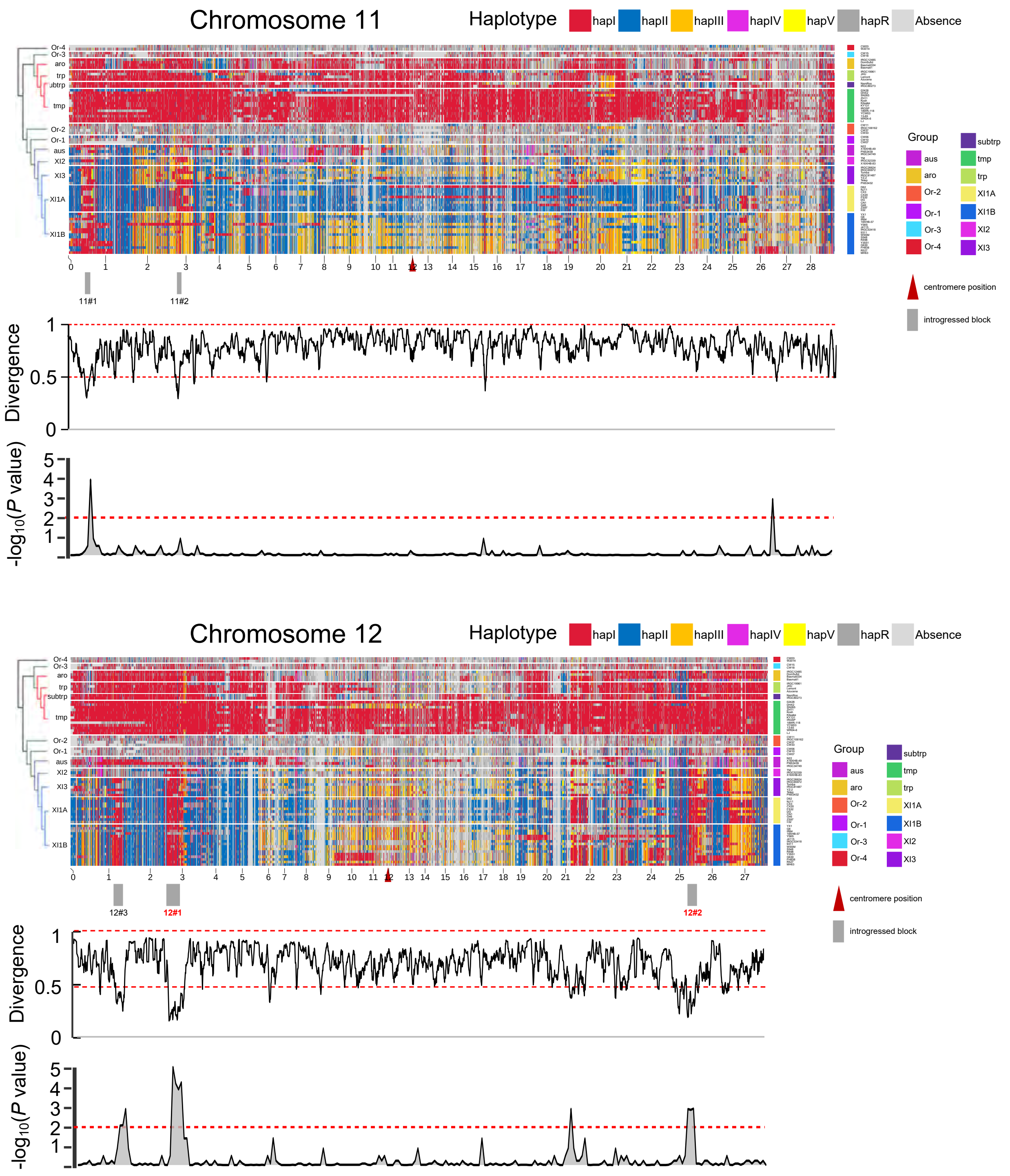
Chromosome 11
Haplotype
hapI
hapII
hapIII
hapIV
hapV
hapR
Absence
Or-4
Or-3
aro
trp
subtrp
tmp
Or-2
Or-1
aus
XI2
XI3
XI1A
XI1B
0 1 2 3 4 5 6 7 8 9 10 11 12 13 14 15 16 17 18 19 20 21 22 23 24 25 26 27 28
11#1
11#2
Group
aus
aro
Or-2
Or-1
Or-3
Or-4
subtrp
tmp
trp
XI1A
XI1B
XI2
XI3
centromere position
introgressed block
Divergence
1
0.5
0
-log10(P value)
5
4
3
2
1
Chromosome 12
Haplotype
hapI
hapII
hapIII
hapIV
hapV
hapR
Absence
Or-4
Or-3
aro
trp
subtrp
tmp
Or-2
Or-1
aus
XI2
XI3
XI1A
XI1B
0 1 2 3 4 5 6 7 8 9 10 11 12 13 14 15 16 17 18 19 20 21 22 23 24 25 26 27
12#3
12#1
12#2
Group
aus
aro
Or-2
Or-1
Or-3
Or-4
subtrp
tmp
trp
XI1A
XI1B
XI2
XI3
centromere position
introgressed block
Divergence
1
0.5
0
-log10(P value)
5
4
3
2
1

Fig. S15 Ancestral haplotype landscape on chromosome 1 to chromosome 12. Putative introgression blocks are shown in gray rectangles and numbered. Haplotype divergence and *P* values (scaled by $-\log_{10}$) of non-random distribution significance tests are shown along each chromosome. Red dashed lines represent thresholds to determine introgression blocks.

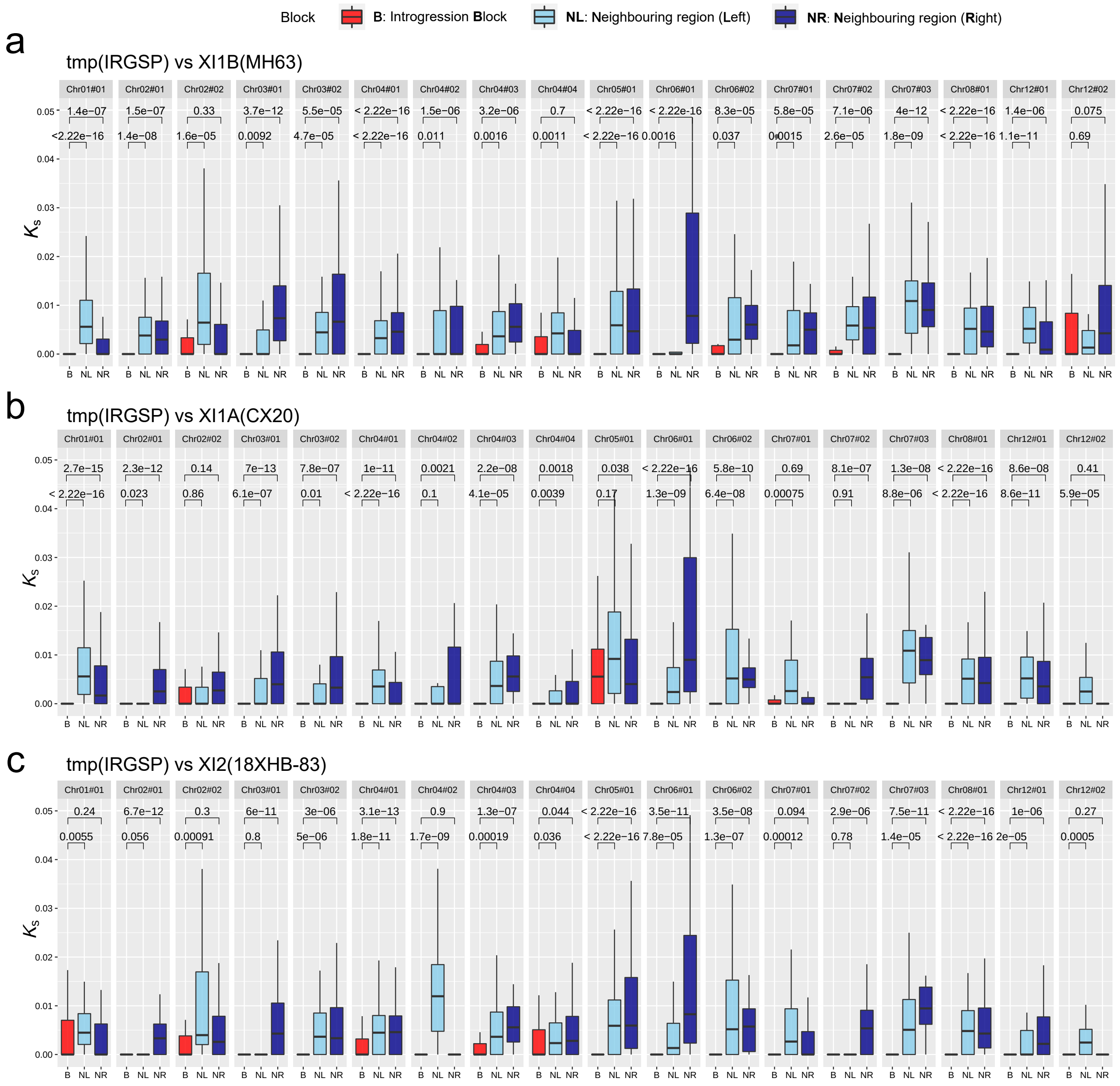

Fig. S16 Synonymous substitution rates (K_s) of genes in putative introgression blocks and their neighboring left and right regions. Three replicates (a, b, and c) between the GJ and XI genomes were performed. In the boxplots, the horizontal line shows the median value, and the whiskers show the 25% and 75% quartile values of K_s. *P* values are calculated using Wilcoxon test.

a

b

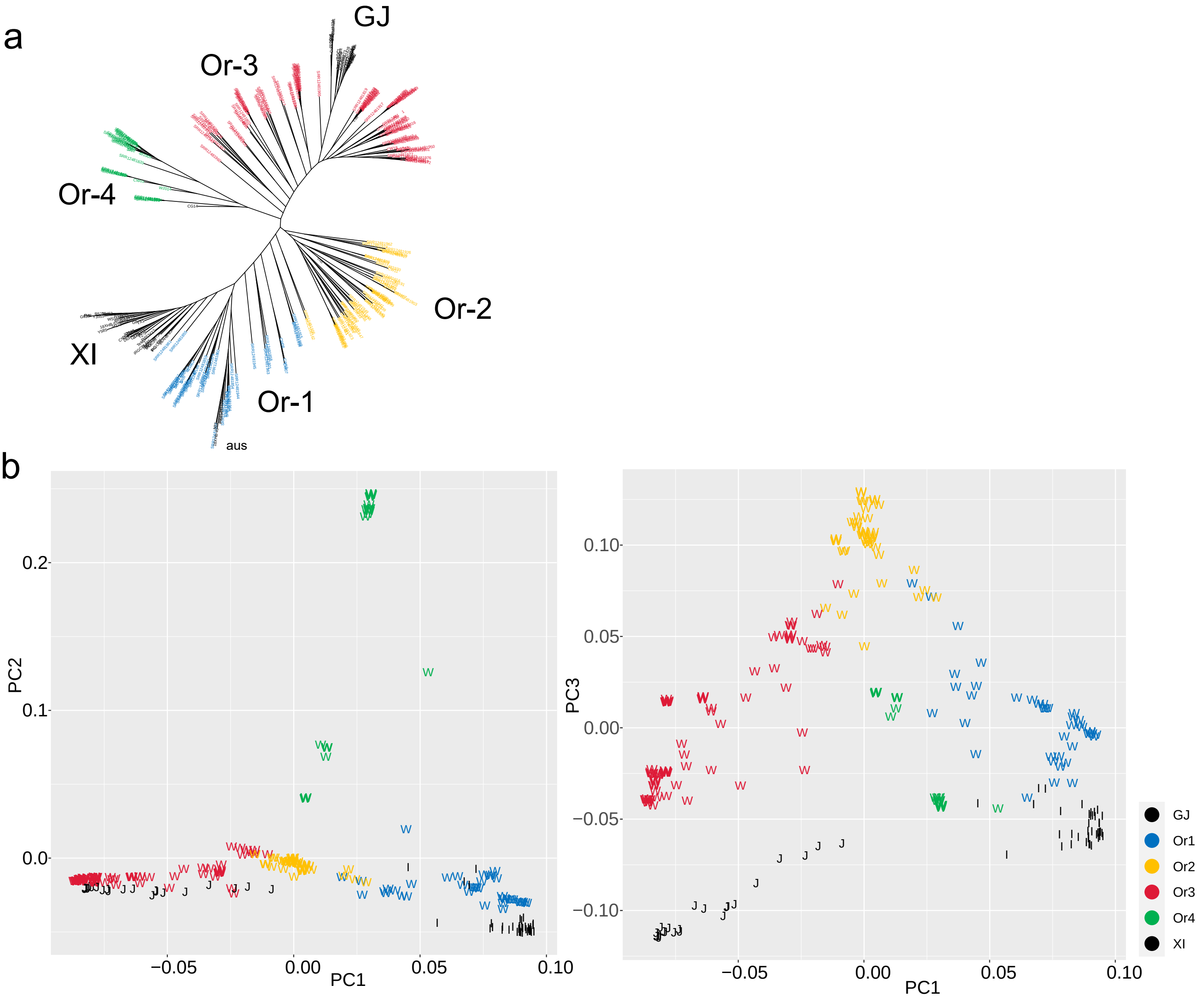

c

Group	India	Nepal	Sri Lanka	Bangladesh	Myanmar	Laos	Thailand	Cambodia	Vietnam	China	Indonesia	Papua New Guinea	Sum
Or-1	7		1	2	1	6	5	6	6	1	2		37
Or-2	1	1	3		4	4	2	5	3	10	4	5	42
Or-3	2	6	4		1		1		2	64			80
Or-4	7	8	5		5								25

Fig. S17 Population structure of wild rice accessions used in this study. (**a**) Phylogenetic tree of *Oryza rufipogon* and *Oryza sativa*. Four wild groups (Or-1, Or-2, Or-3 and Or-4) are indicated in different colors. (**b**) PCA plots of the first three principle components, where "W", "J" and "I" represent wild, GJ and XI accessions. (**c**) Geographical sources of wild accessions in different groups used in this study

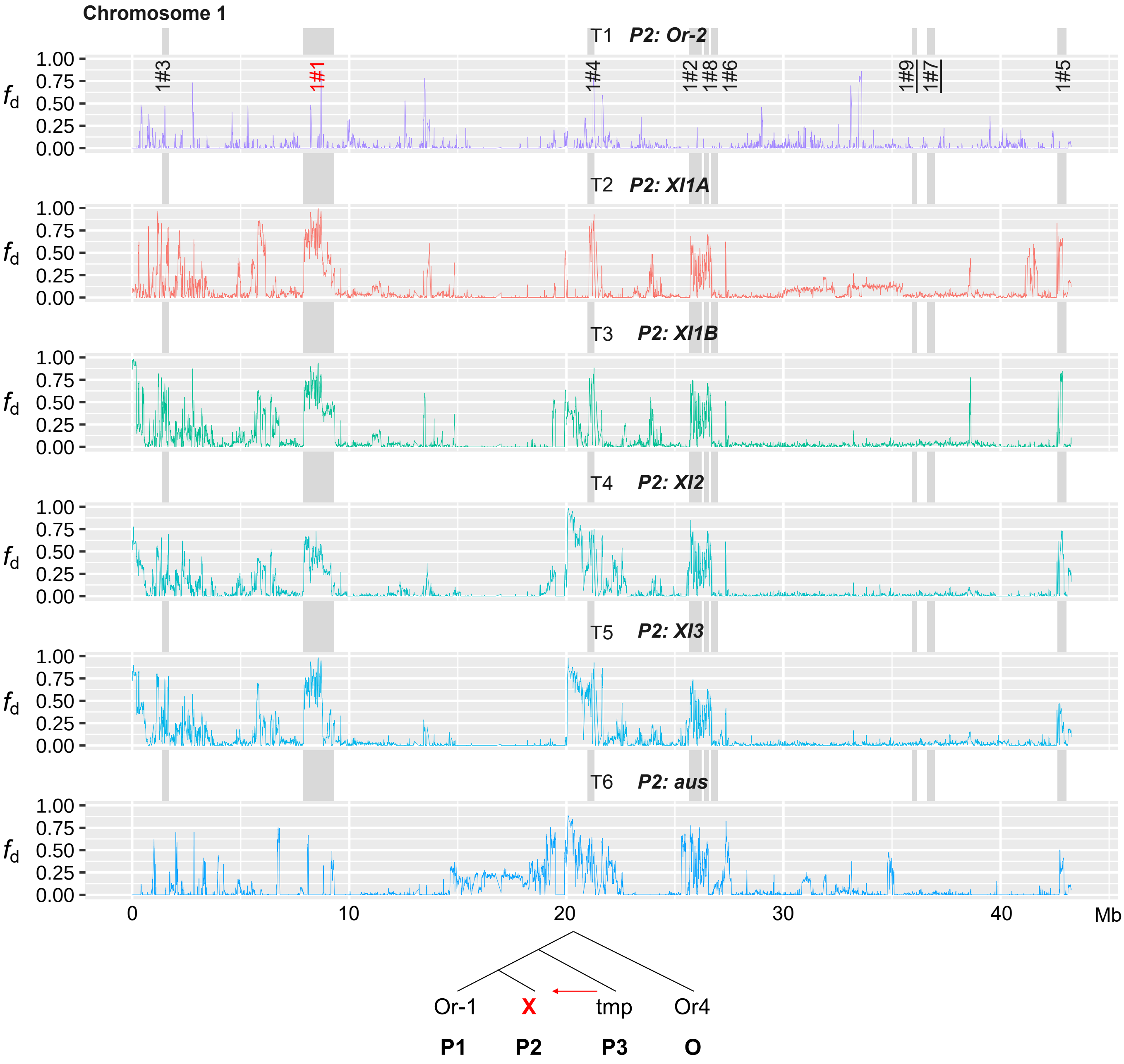

Fig. S18 Introgression fd distributions of ABBA-BABA test on chromosome 1 to chromosome 12 in topology T1 to T6. P1 is Or-1 (*n* = 37), P3 is tmp (GJ, *n* = 100), O/outgroup is Or-4 (*n* = 25), and P2 was set as Or-2 (*n* = 42), XI1A (*n* = 100), XI1B (*n* = 100), XI2 (*n* = 80), XI3 (*n* = 100) and aus (n=60), respectively. T1 is set as a background control in introgression detection. Genomic positions of putative introgression regions are indicated by gray rectangles and detailed coordinates are provided in Supplementary Table 2. Blocks larger than 300kb are highlighted in red and blocks not supported by f_d are underlined.

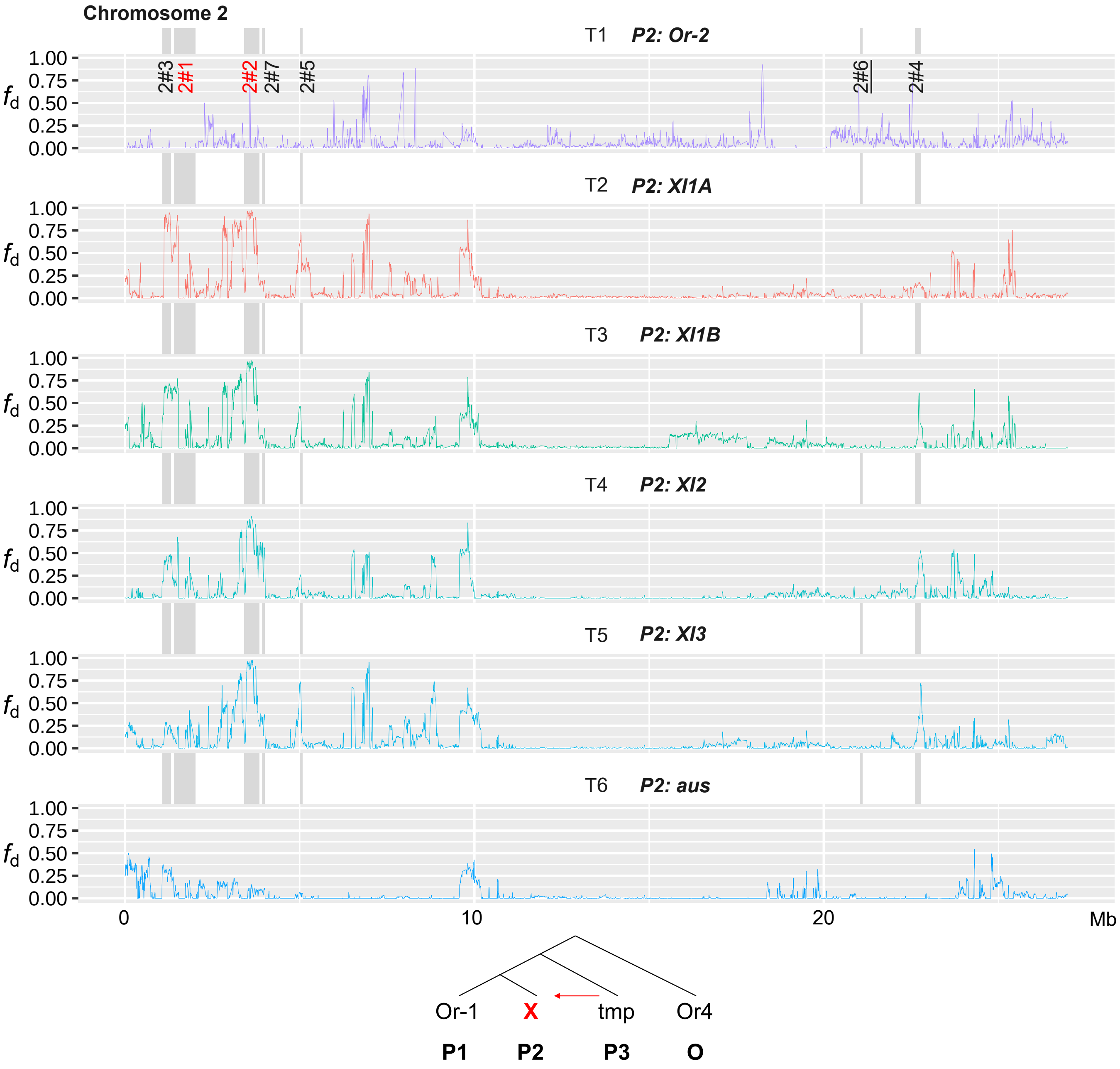
Chromosome 2
T1 P2: Or-2
T2 P2: Xl1A
T3 P2: Xl1B
T4 P2: Xl2
T5 P2: Xl3
T6 P2: aus
2#3
2#1
2#2
2#7
2#5
2#6
2#4
f_d
1.00
0.75
0.50
0.25
0.00
0
10
20
Mb
Or-1
X
tmp
Or4
P1
P2
P3
O

Fig. S18

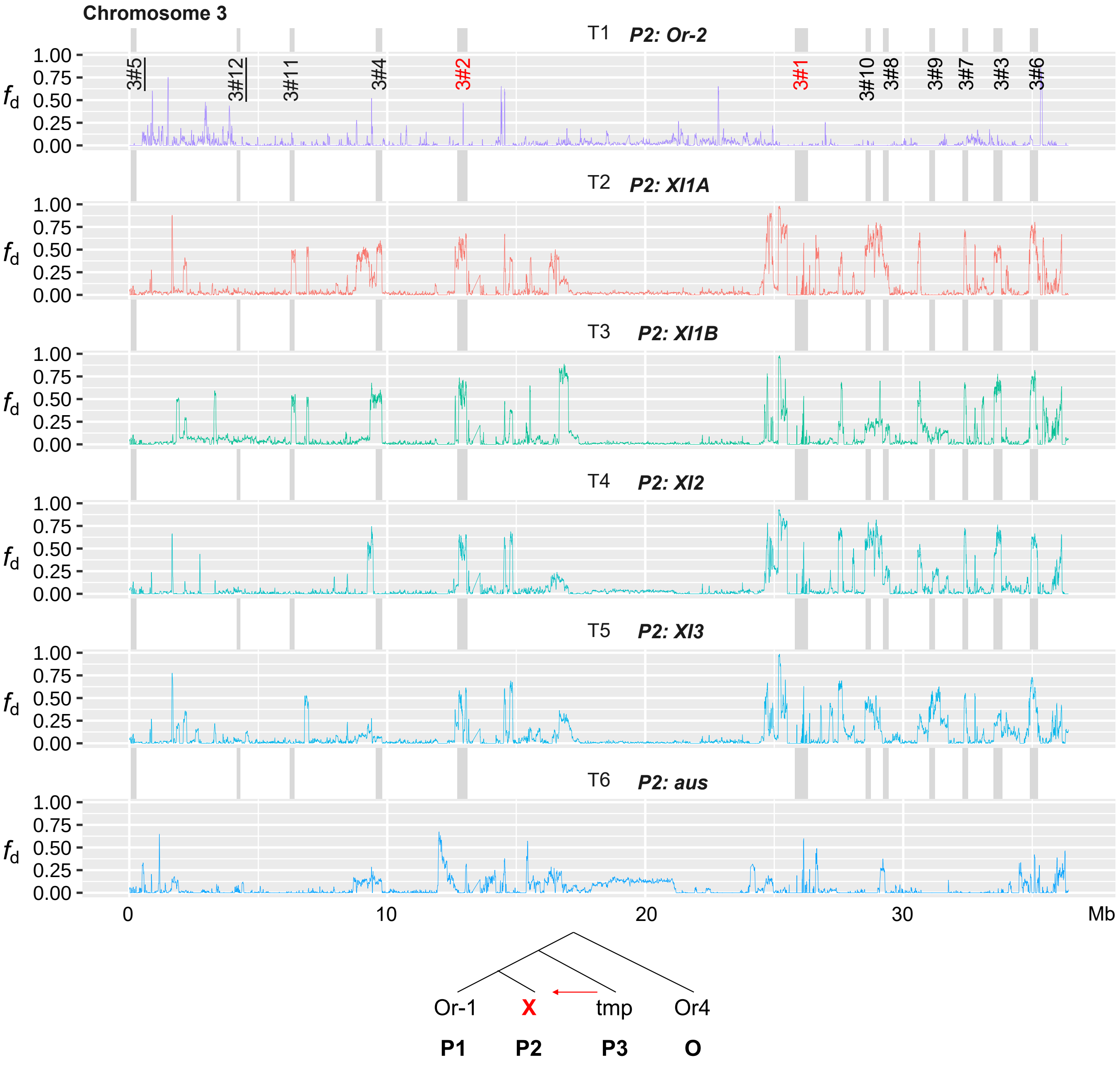
Chromosome 3
T1 P2: Or-2
T2 P2: Xl1A
T3 P2: Xl1B
T4 P2: Xl2
T5 P2: Xl3
T6 P2: aus
3#5
3#12
3#11
3#4
3#2
3#1
3#10
3#8
3#9
3#7
3#3
3#6
fd
1.00
0.75
0.50
0.25
0.00
0
10
20
30
Mb
Or-1
X
tmp
Or4
P1
P2
P3
O

Fig. S18

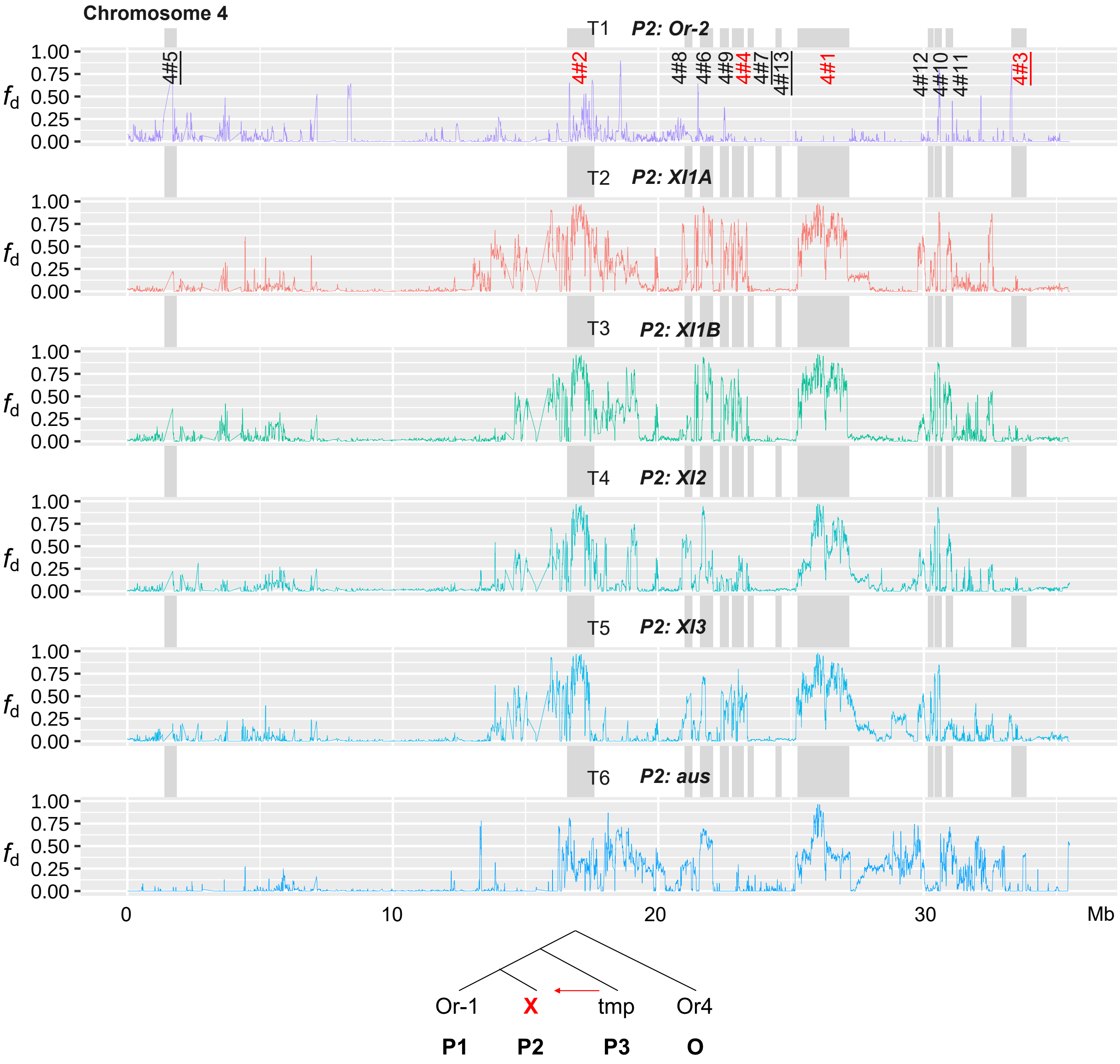
Chromosome 4
T1 P2: Or-2
T2 P2: XI1A
T3 P2: XI1B
T4 P2: XI2
T5 P2: XI3
T6 P2: aus
4#5
4#2
4#8
4#6
4#9
4#4
4#7
4#13
4#1
4#12
4#10
4#11
4#3
fd
1.00
0.75
0.50
0.25
0.00
0
10
20
30
Mb
Or-1
X
tmp
Or4
P1
P2
P3
O

Fig. S18

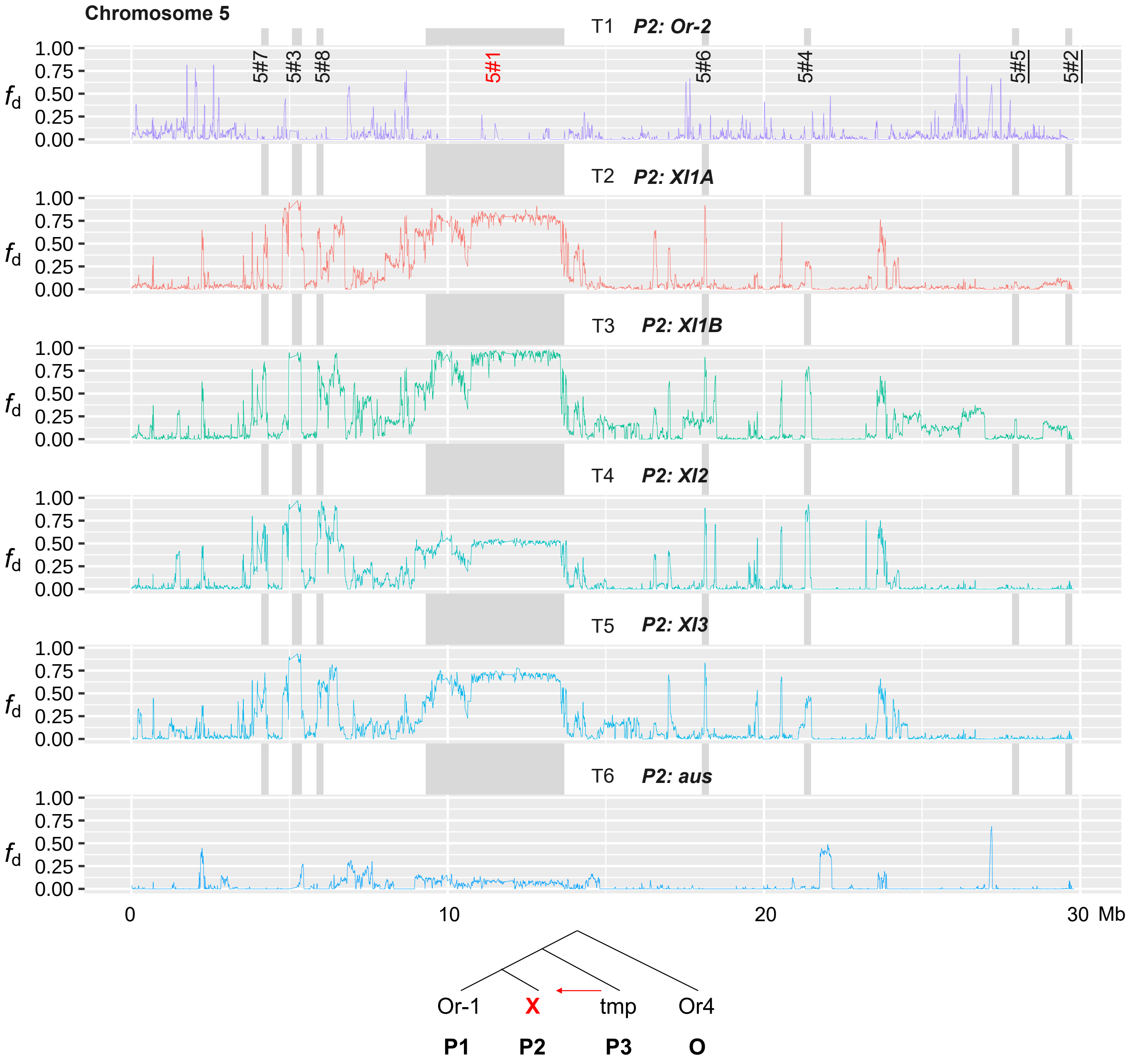

Chromosome 5
T1 P2: Or-2
T2 P2: Xl1A
T3 P2: Xl1B
T4 P2: Xl2
T5 P2: Xl3
T6 P2: aus
5#7
5#3
5#8
5#1
5#6
5#4
5#5
5#2
1.00
0.75
0.50
0.25
0.00
f_d
0
10
20
30 Mb
Or-1
X
tmp
Or4
P1
P2
P3
O

Fig. S18

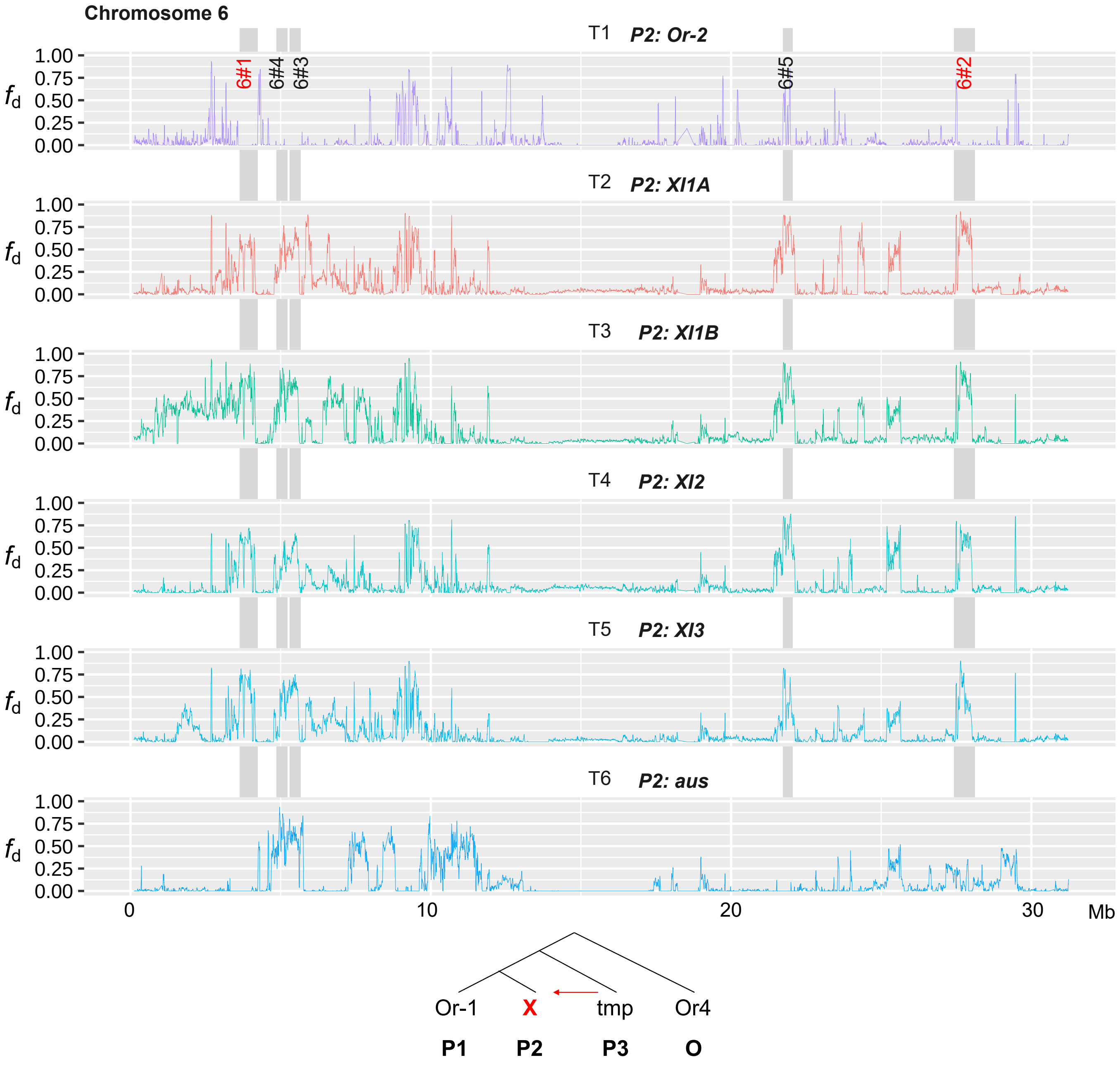

Chromosome 6
T1 P2: Or-2
T2 P2: Xl1A
T3 P2: Xl1B
T4 P2: Xl2
T5 P2: Xl3
T6 P2: aus
6#1
6#4
6#3
6#5
6#2
f_d
1.00
0.75
0.50
0.25
0.00
0
10
20
30
Mb
Or-1
X
tmp
Or4
P1
P2
P3
O

Fig. S18

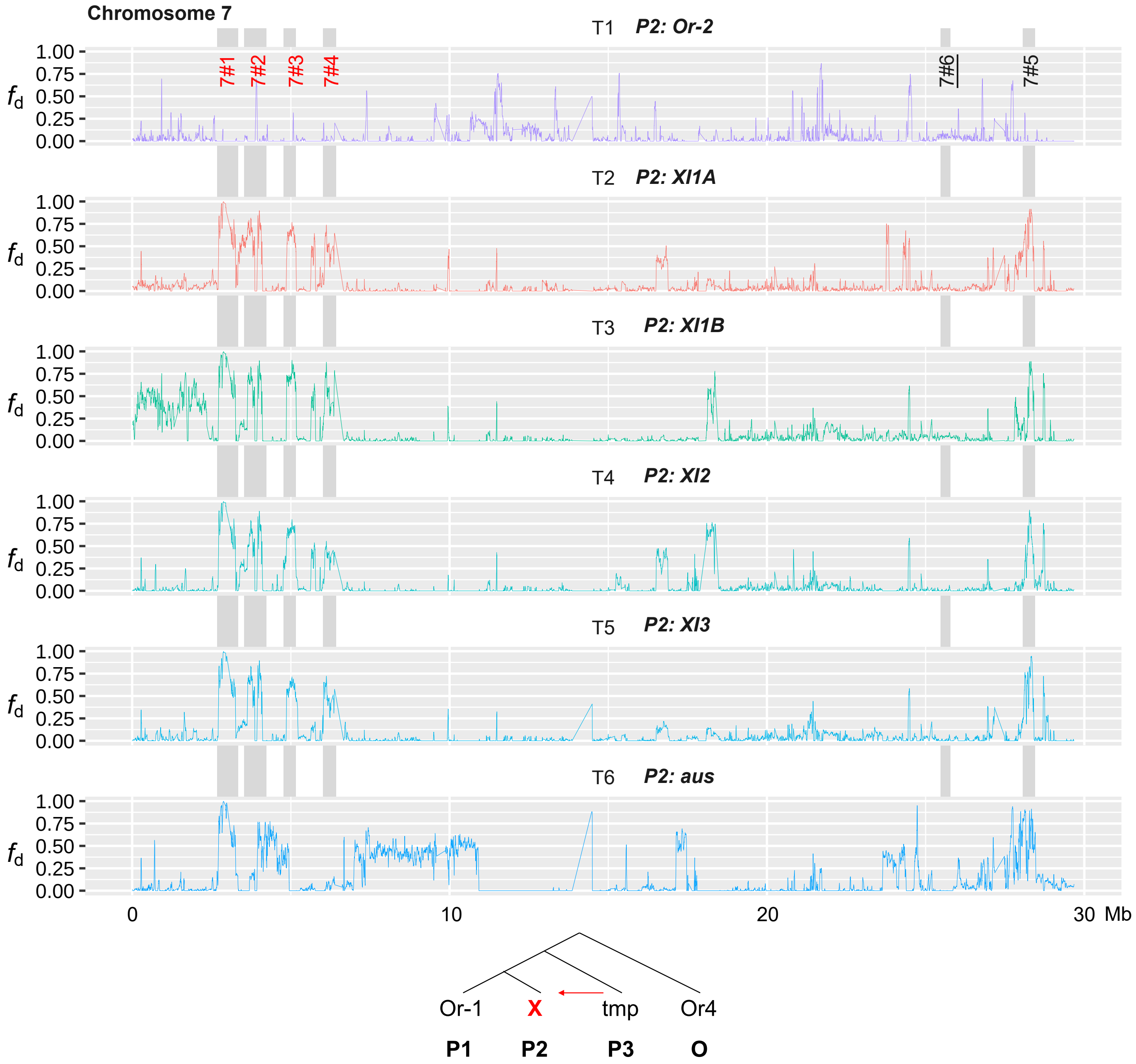

Chromosome 7
T1 P2: Or-2
7#1
7#2
7#3
7#4
7#6
7#5
T2 P2: Xl1A
T3 P2: Xl1B
T4 P2: Xl2
T5 P2: Xl3
T6 P2: aus
1.00
0.75
0.50
0.25
0.00
f_d
0
10
20
30 Mb
Or-1
X
tmp
Or4
P1
P2
P3
O

Fig. S18

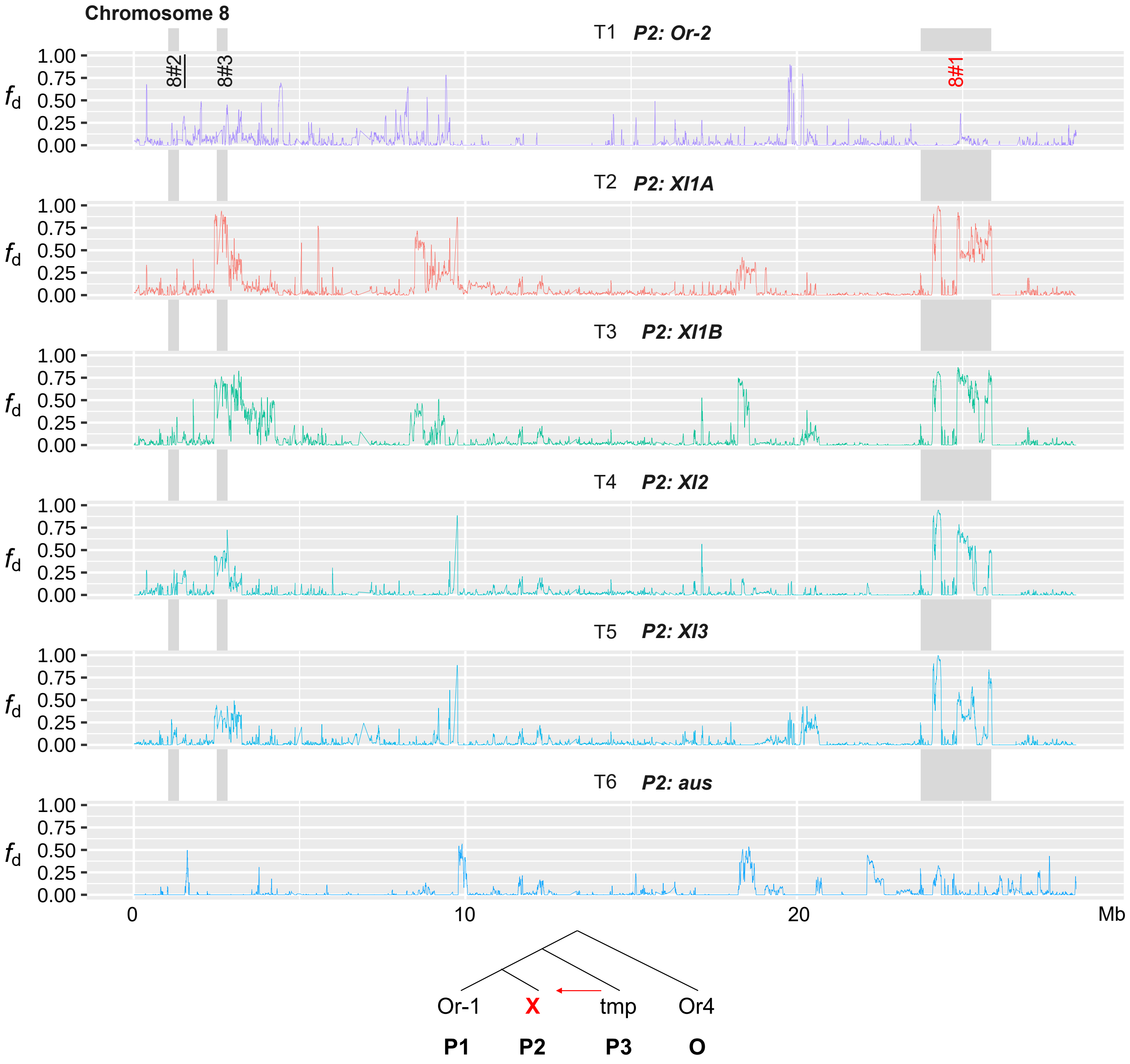
Chromosome 8
T1 P2: Or-2
8#2
8#3
8#1
T2 P2: XI1A
T3 P2: XI1B
T4 P2: XI2
T5 P2: XI3
T6 P2: aus
f_d
1.00
0.75
0.50
0.25
0.00
0
10
20
Mb
Or-1
X
tmp
Or4
P1
P2
P3
O

Fig. S18

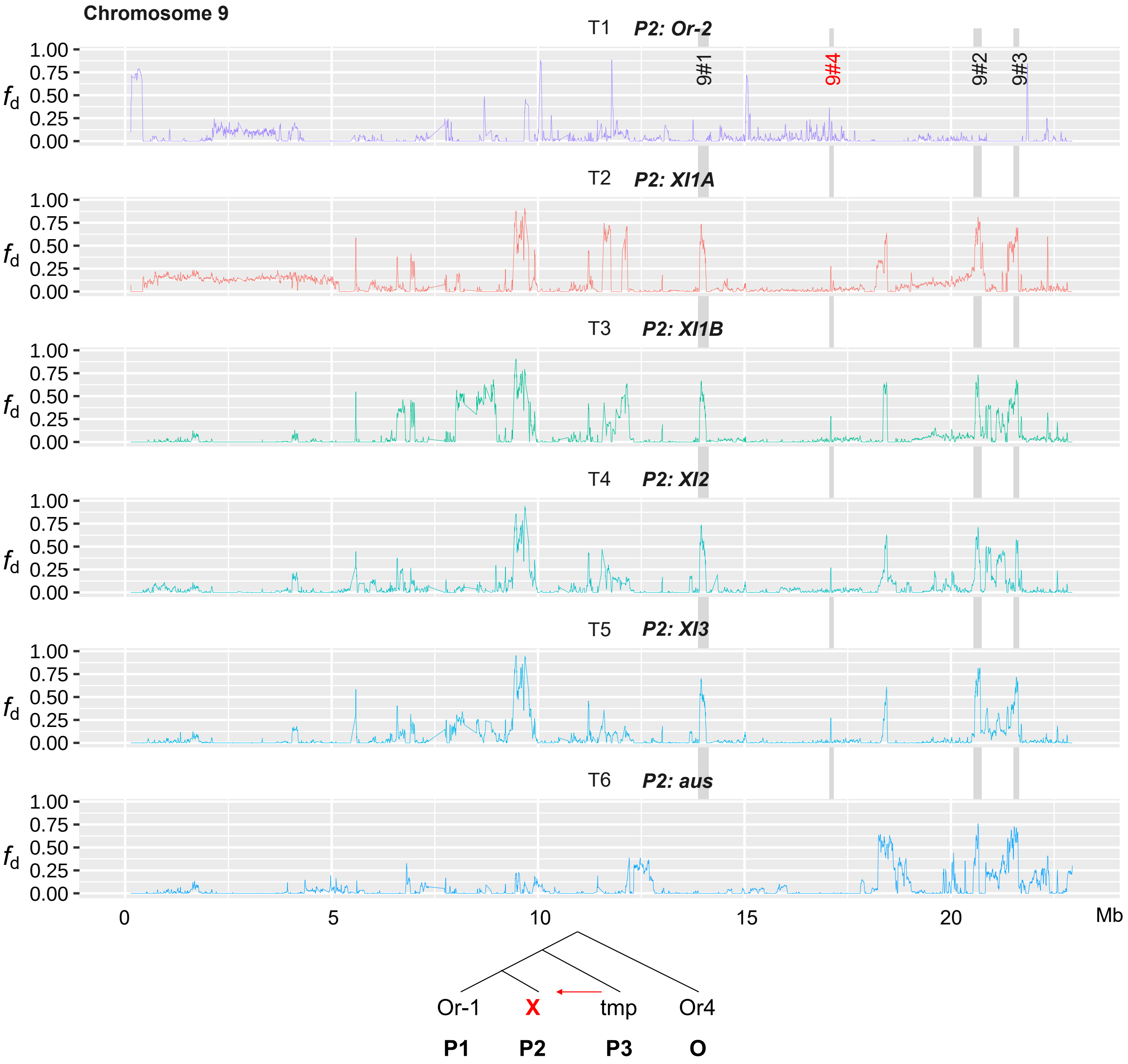

Chromosome 9
T1 P2: Or-2
T2 P2: XI1A
T3 P2: XI1B
T4 P2: XI2
T5 P2: XI3
T6 P2: aus
9#1
9#4
9#2
9#3
f_d
1.00
0.75
0.50
0.25
0.00
0
5
10
15
20
Mb
Or-1
X
tmp
Or4
P1
P2
P3
O

Fig. S18

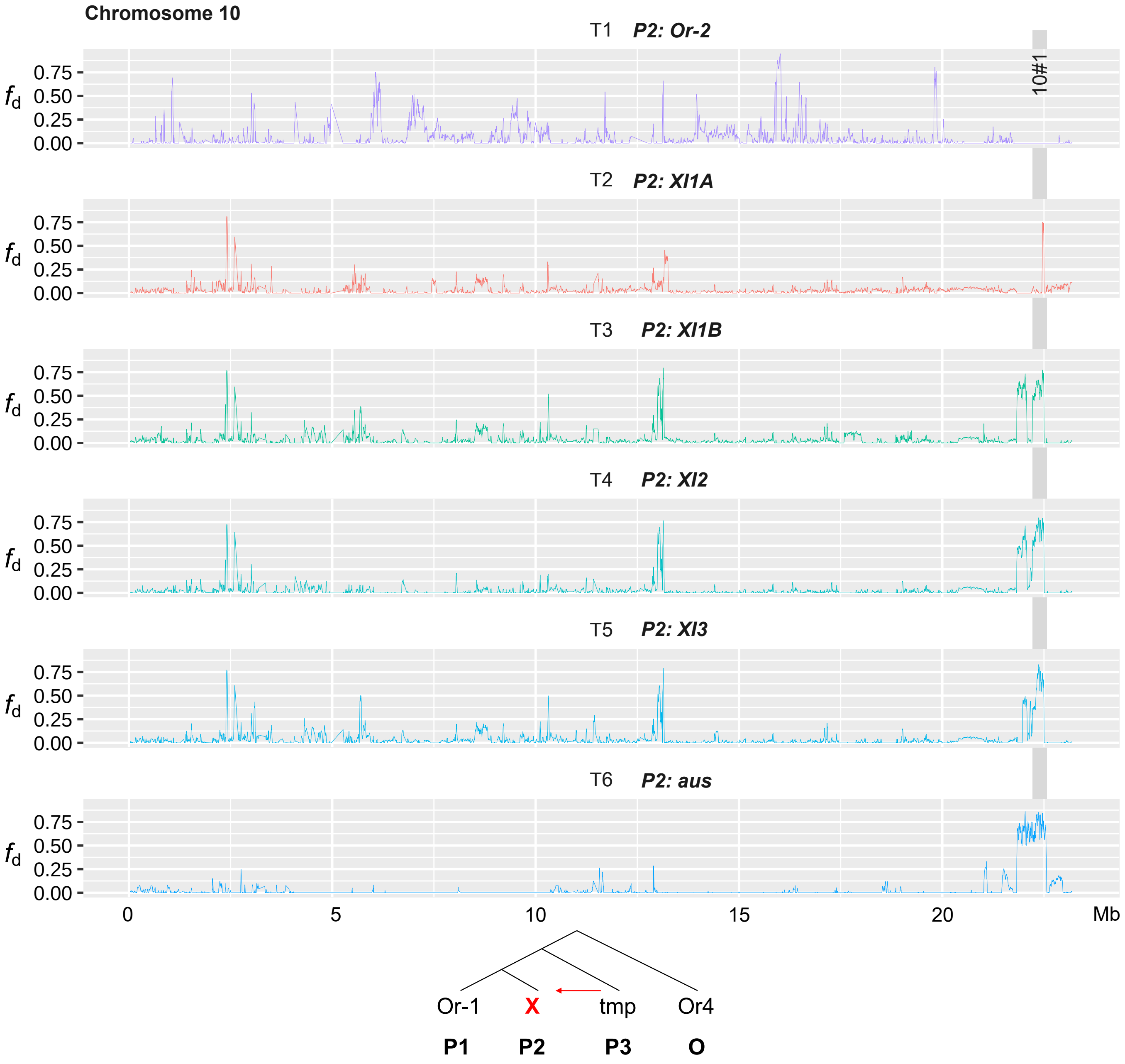
Chromosome 10
T1 P2: Or-2
T2 P2: XI1A
T3 P2: XI1B
T4 P2: XI2
T5 P2: XI3
T6 P2: aus
10#1
f_d
0.00
0.25
0.50
0.75
0
5
10
15
20
Mb
Or-1
X
tmp
Or4
P1
P2
P3
O

Fig. S18

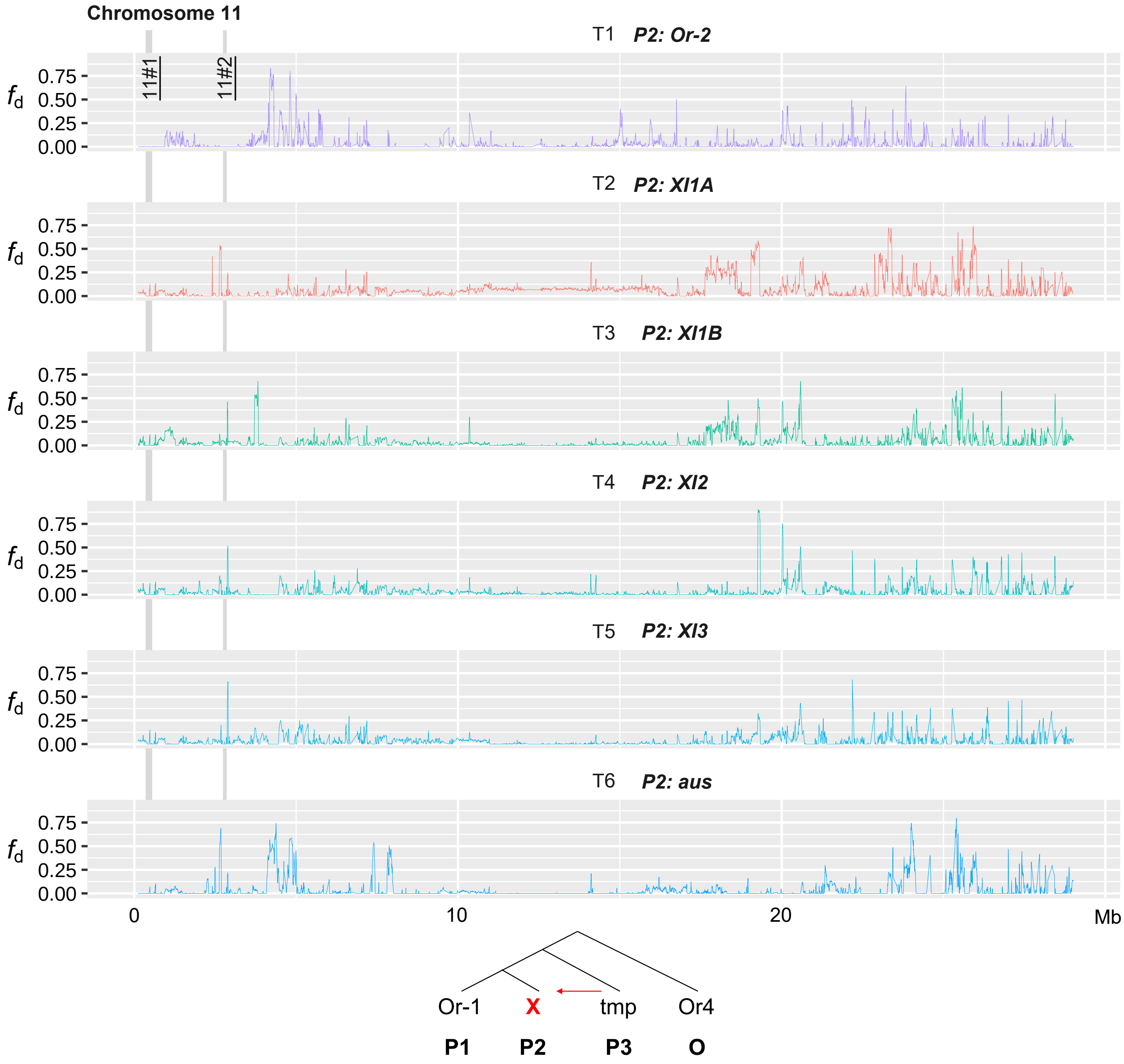
Chromosome 11
11#1
11#2
T1 P2: Or-2
T2 P2: XI1A
T3 P2: XI1B
T4 P2: XI2
T5 P2: XI3
T6 P2: aus
f_d
0.75
0.50
0.25
0.00
0
10
20
Mb
Or-1
X
tmp
Or4
P1
P2
P3
O

Fig. S18

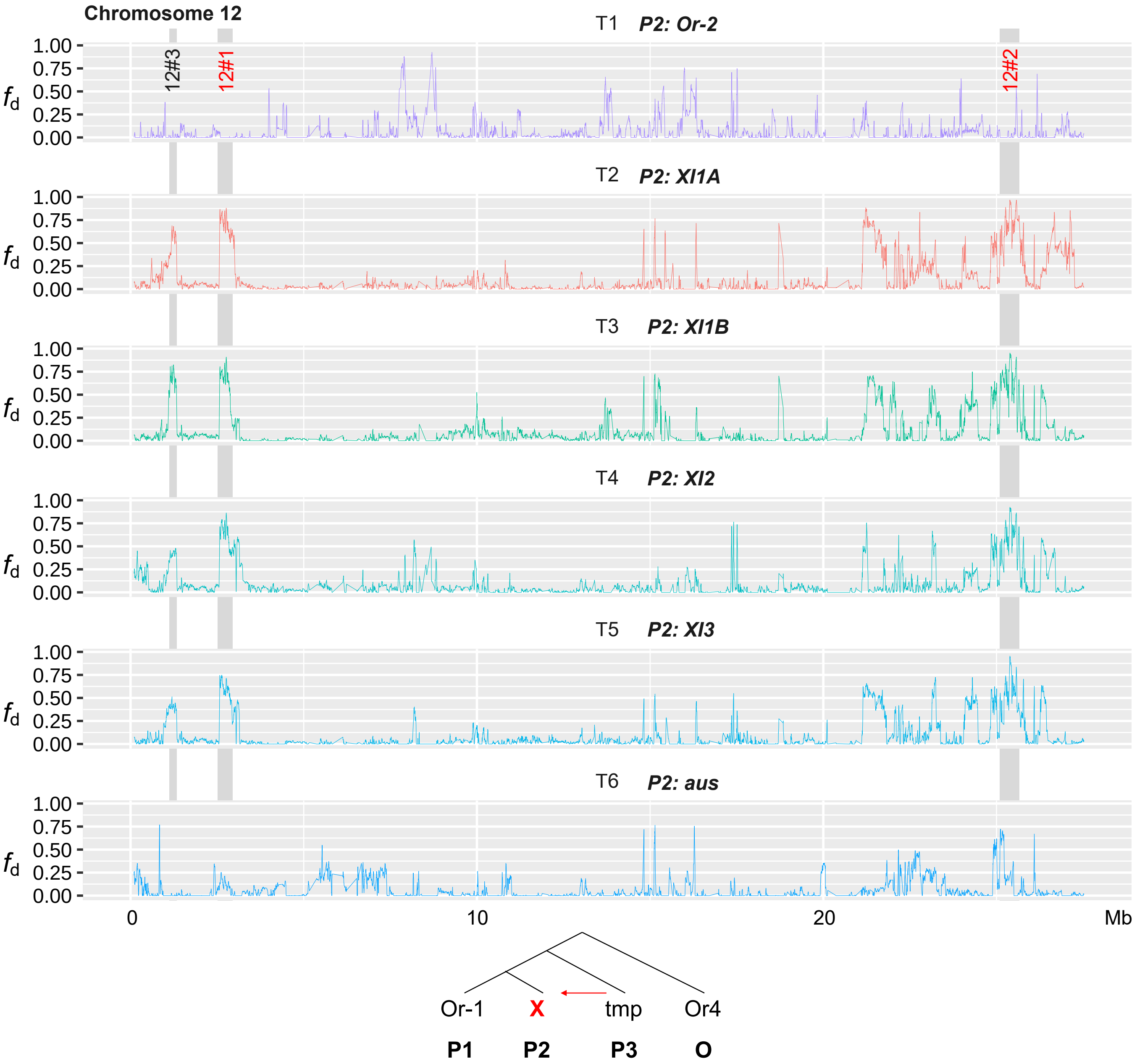
Chromosome 12
T1 P2: Or-2
T2 P2: XI1A
T3 P2: XI1B
T4 P2: XI2
T5 P2: XI3
T6 P2: aus
12#3
12#1
12#2
fd
1.00
0.75
0.50
0.25
0.00
0
10
20
Mb
Or-1
X
tmp
Or4
P1
P2
P3
O

Fig. S18

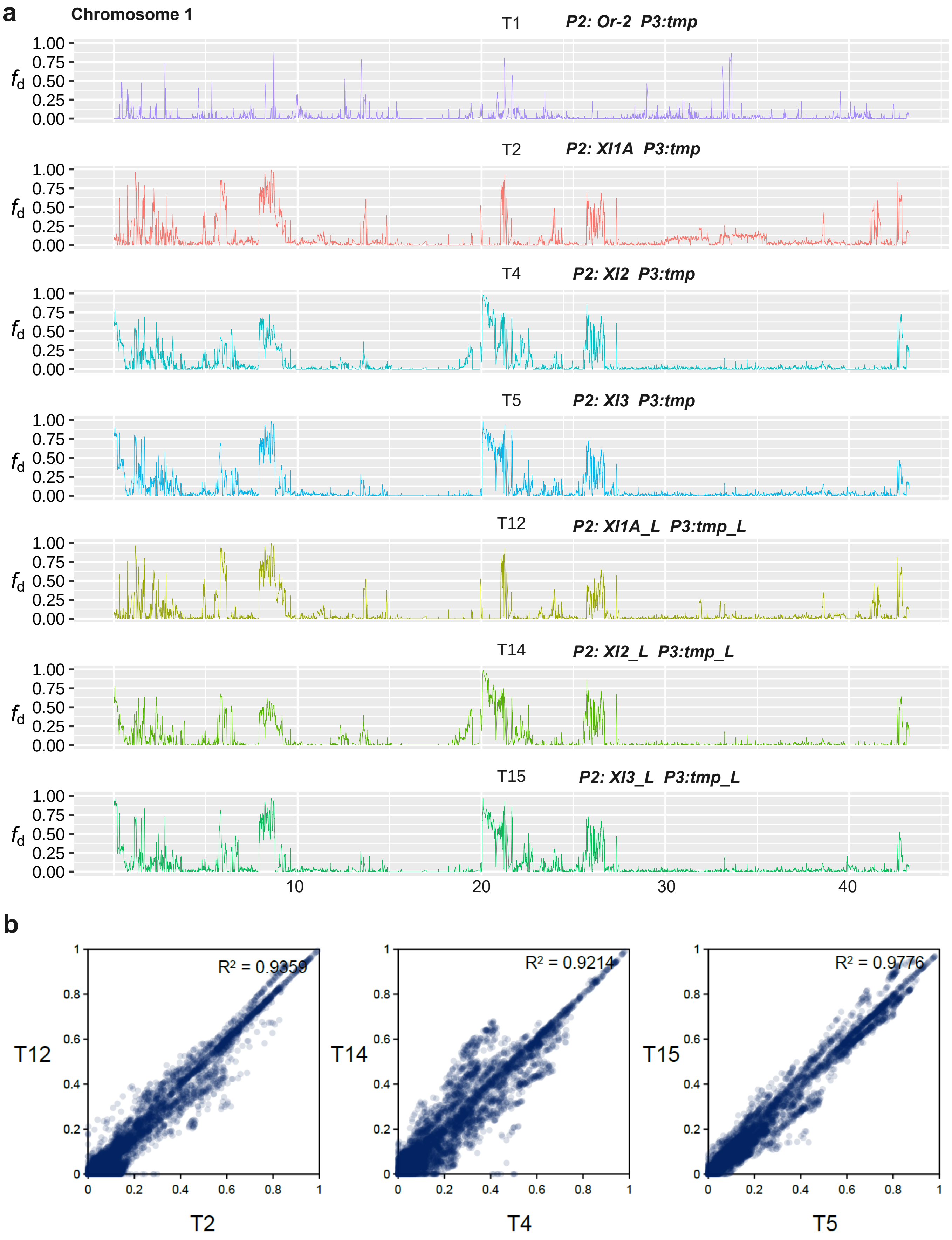

a
Chromosome 1
T1 P2: Or-2 P3:tmp
T2 P2: Xl1A P3:tmp
T4 P2: Xl2 P3:tmp
T5 P2: Xl3 P3:tmp
T12 P2: Xl1A_L P3:tmp_L
T14 P2: Xl2_L P3:tmp_L
T15 P2: Xl3_L P3:tmp_L
f_d
1.00
0.75
0.50
0.25
0.00
10
20
30
40
b
R^2 = 0.9359
R^2 = 0.9214
R^2 = 0.9776
T12
T14
T15
T2
T4
T5
0
0.2
0.4
0.6
0.8
1

Fig. S19 Comparison of f_d using all genomes and landraces only under different topologies on chromosome 1. (**a**) f_d distribution along chromosome 1. Group tmp, XI1A, XI2, XI3, and *aus* include 47, 24, 21, 52 and 32 landrace accessions, respectively. (**b**) Comparison of f_d on chromosome 1 in T2 *vs* T12, T4 *vs* T14, and T5 *vs* T15.

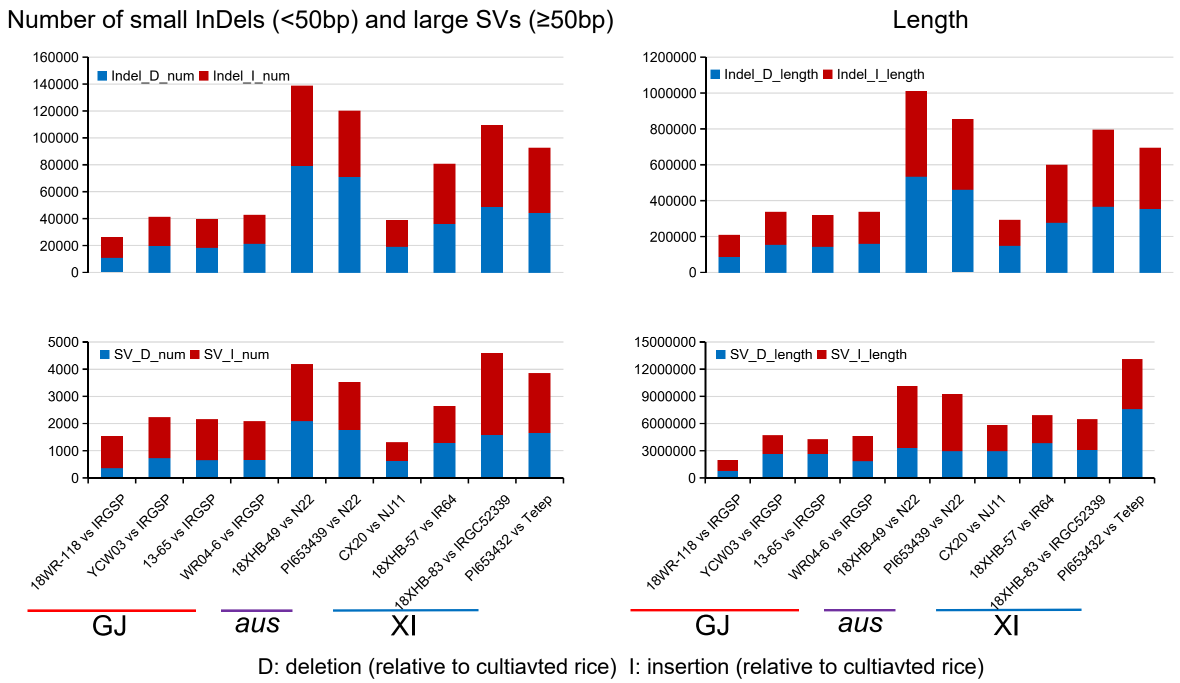

Fig. S20 Summary of structural variations between weedy and cultivated rice genomes.

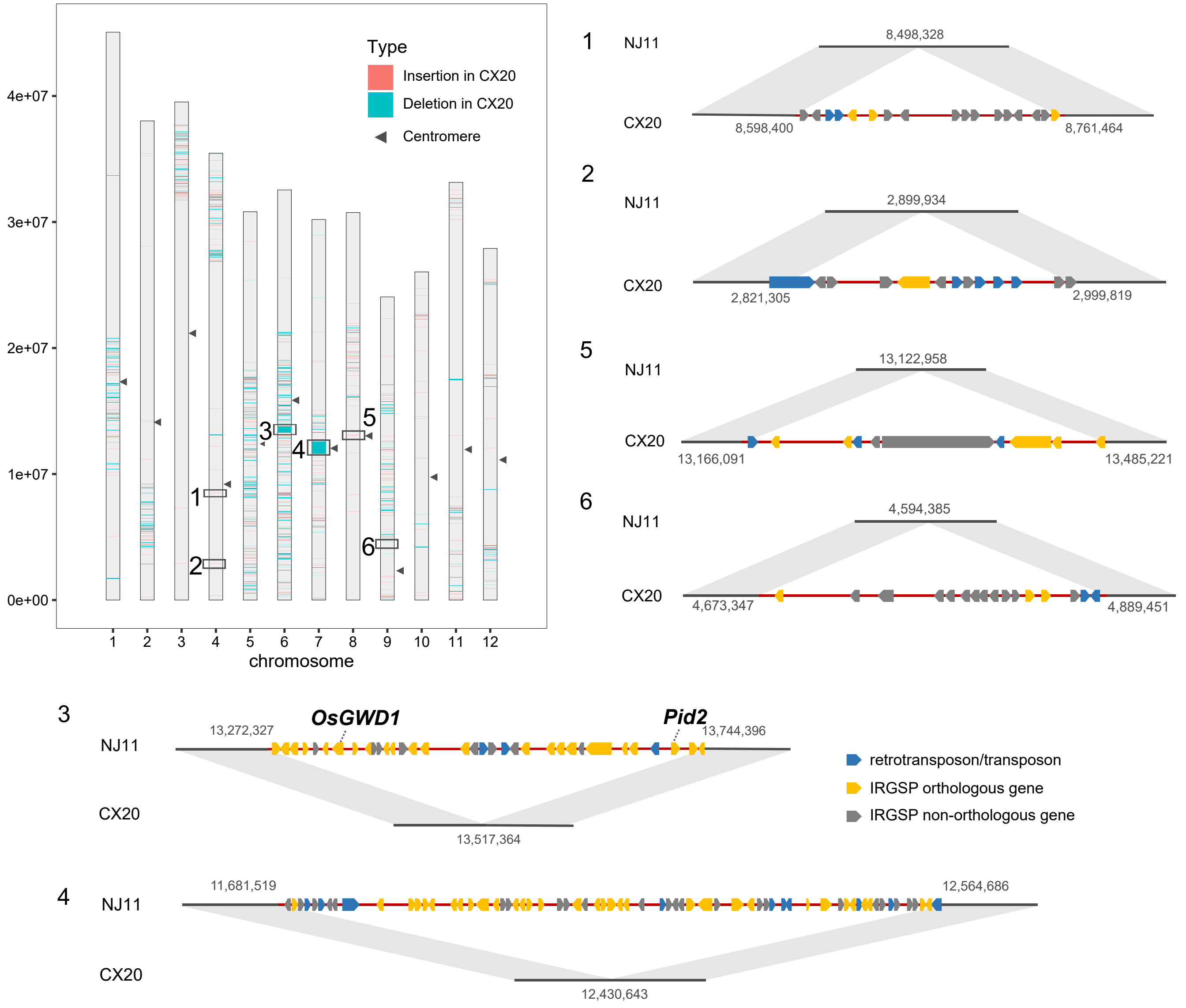

Fig. S21 Structural variations (>50bp) between assemblies of cultivar accession NJ11 and weedy rice accession CX20 on 12 chromosomes. The largest six SVs (numbered from 1 to 6) are zoomed in and annotated, including four insertions (1, 2, 5 and 6) and two deletions (3 and 4) in CX20.

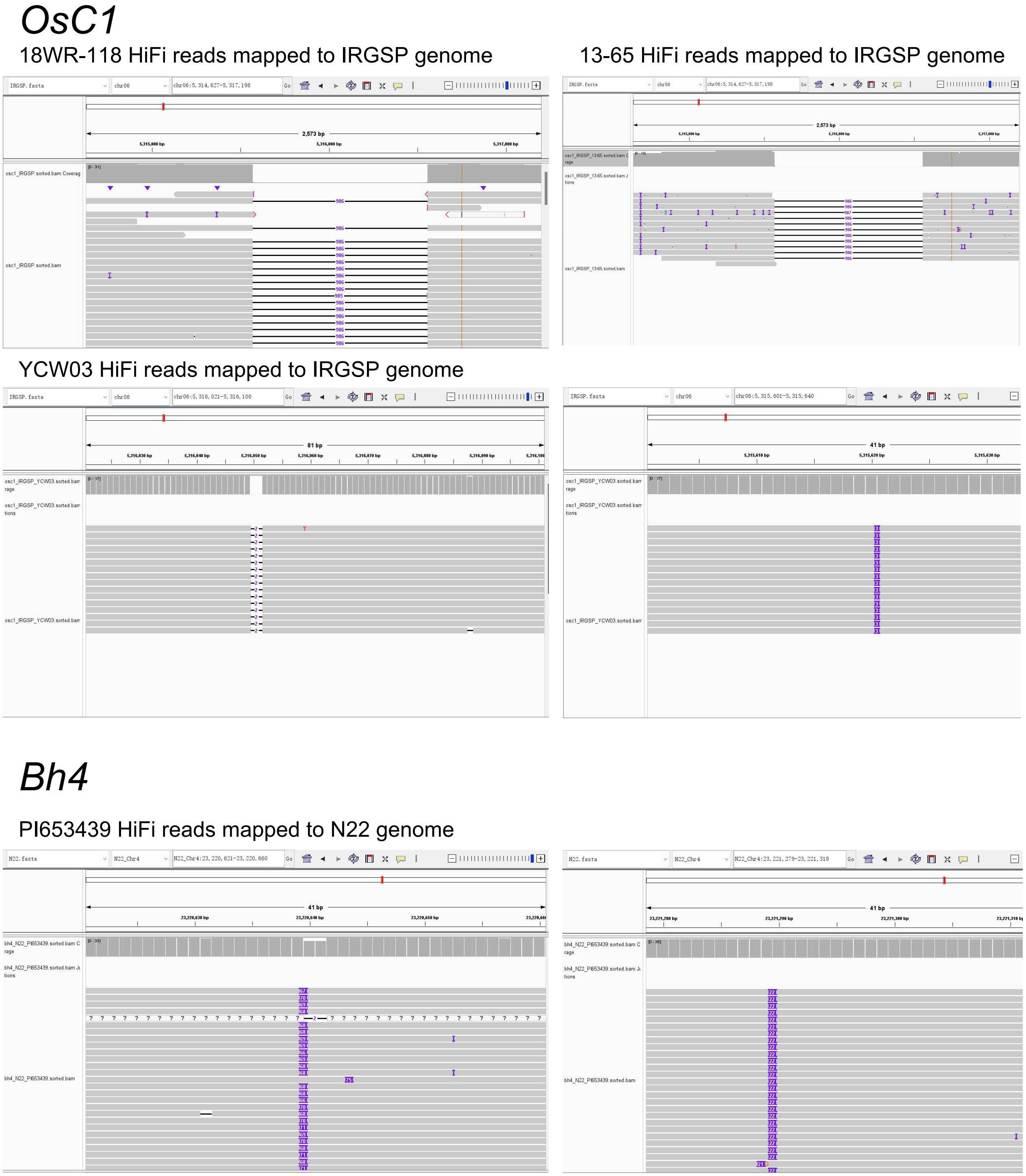

Fig. S22 The Integrative Genomics Viewer (IGV) snapshots show the structural variations in *OsC1* and *Bh4* between weedy and cultivated rice.

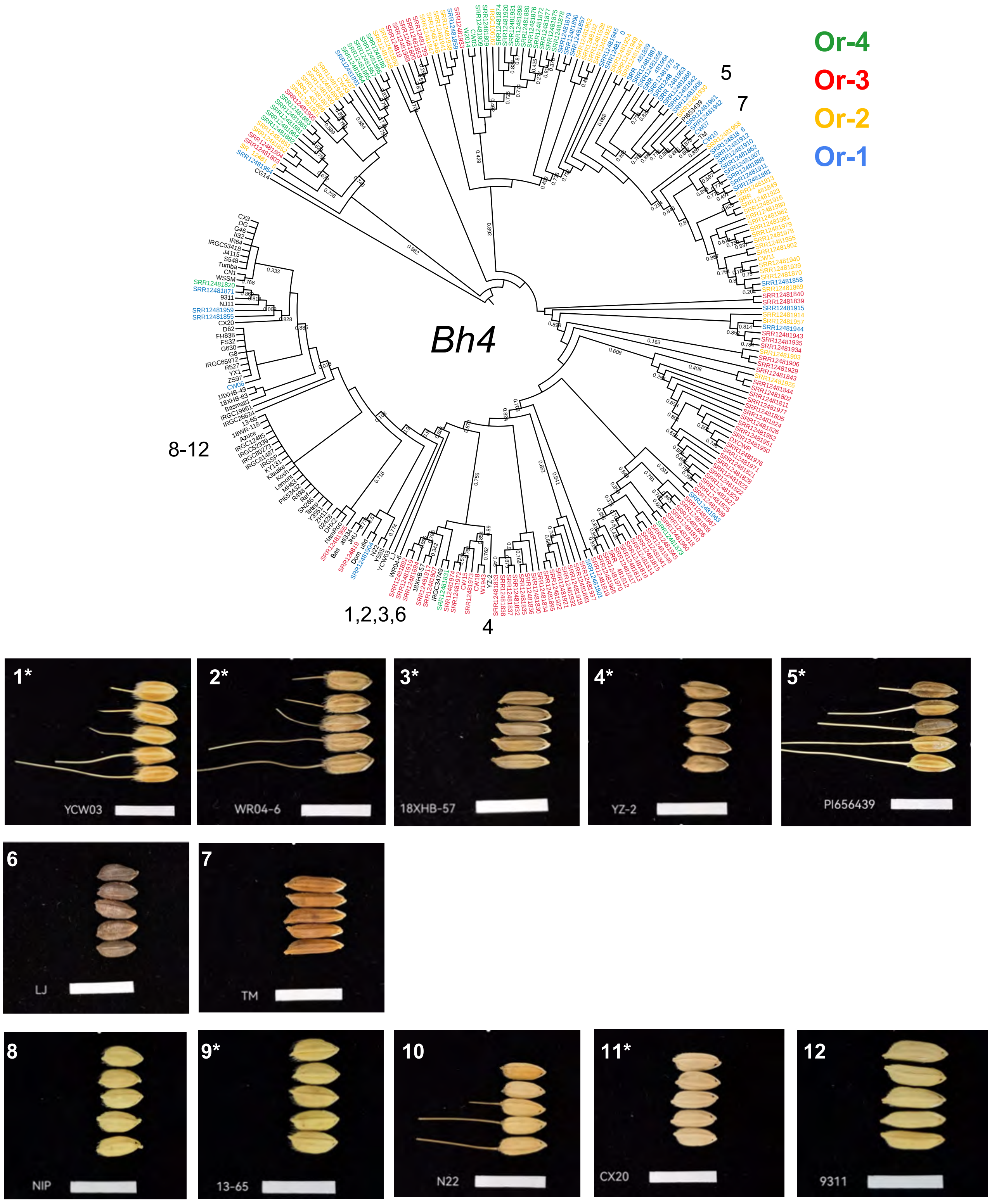

Fig. S23 ML phylogeny tree of *Bh4* in rice and morphology of rice seed hulls. Bootstrap values less than 0.90 are indicated on branches.
