# Supplementary material for: A syntelog-based pan-genome provides insights into rice domestication and de-domestication
Source: Genome Biol. 2023 Aug 3;24:179. doi: 10.1186/s13059-023-03017-5 (PMC10401782; doi:10.1186/s13059-023-03017-5)
Supplement: Supplementary file 3 — Additional file 3: Supplemental Note 1. Base-level quality evaluation of rice genomes. Supplemental Note 2. Rice NLRome. [file 13059_2023_3017_MOESM3_ESM.docx]

**Additional file 3: Supplemental Notes**

**Supplemental Note 1. Base-level quality evaluation of rice genomes**

Base-level accuracy of *de novo* assemblies in this study and previous released genomes based third-generation sequencing (through May 2022) were assessed in base level. The *k*-mer-based assembly validation results revealed higher assembly consensus quality values (QVs) for HiFi assemblies generated in this study (average QV = 44.16) than those for previous assemblies (Fig. 1a, Additional file 1: Fig. S4a). We quantified the assembly accuracy by calling homozygous single nucleotide polymorphisms (SNPs) and short insertions and deletions (InDels) by mapping available NGS reads for each accession against its own assembly. Raw short-read data were first cleaned by NGSQC-toolkit and mapped against the corresponding assembly by Bowtie2. Variants were detected using GATK (v3.7, default parameters) (McKenna et al., 2010) and annotated by SnpEff (v3.6) to profile their potential effects on the prediction of amino acid sequences and further gene functions (Cingolani et al., 2012). At the single-base level, averagely the HiFi assemblies showed fewer errors than the PacBio CLR mode and Nanopore sequencing (Fig. 1a, Additional file 1: Fig. S4b). In terms of InDels, HiFi assemblies showed no obvious differences from CLR mode assemblies, but there were fewer InDels in HiFi than in Nanopore assemblies. The annotation of the potential assembly errors for each accession suggested high (stop loss and gain, start loss, and frame-shift variants) and moderate effects (inframe insertion/deletion and missense variants) in the predicted gene models (Additional file 1: Fig. S4b). The low assembly quality at the base level directly interferes with the accuracy of haplotype inference, especially for Nanopore-based assemblies despite further polishing using short reads; thus, the recently released Nanopore-based assemblies of cultivated rice were excluded. Given that the available assemblies for wild rice using PacBio sequencing were only for W2014 (Ma et al., 2020) and IRGC106162 (Xie et al., 2020), nine Nanopore-based wild assemblies (Shang et al., 2022) were adopted. Finally 74 genomes (11 accessions of wild rice *Oryza rufipogon*, 51 of cultivated rice *Oryza sativa* and 12 of weedy rice *Oryza sativa* ssp. *spontanea*) were used in the following pan-genomic analysis. Genome assembly of African rice (*Oryza glaberrima*) accession CG14 was included as an outgroup (Qin et al., 2020).

**Supplemental Note 2. Rice NLRome**

Pan-genomes provide an opportunity to uncover the diversity of highly variable gene families, such as those encoding nucleotide-binding leucine-rich repeat (NLR) proteins related to disease resistance, across species (so-called pan-NLRome). we integrated multiple software predictions and gene synteny in rice genomes to obtain a comprehensive and complete rice NLRome. The NB-ARC domain was first predicted using hmmsearch (HMMER v3.1b2) against the Pfam database (v30.0) with a threshold *e*-value less than 1e-5. The LRR domains were predicted with NLR-parser (v3.0) (Steuernagel et al., 2015) by searching for motifs 9, 11 and 19; the coil domains were predicted by searching for motifs 16 and 17; and the TIR domains were predicted by searching for motifs 13, 15 and 18. All putative NLR types from genome-wide protein sequences were also determined using RGAugury (Li et al., 2016). After the NLR genes for each genome were identified by domain prediction, the NLR genes were mapped back to the SG-based pangenome to involve NLR syntelogs lacking canonical domains and find a more comprehensive and extensive NLR inventory.A total of 37,079 NLR genes in 74 rice genomes were identified by integrating NLR domains and SG information, and categorized into 998 SGs, ranging from 452 (W2014 from wild group Or-4) to 532 (FH838 from group XI1B) NLRs per genome. In total, 0.64% (*n* = 238) of all NLRs were present in only one accession, representing 238 private SGs, while the remaining 10,878 (29.3%), 14,760 (39.8%) and 11,203 (30.2%) NLRs grouped into 147 core, 206 soft-core and 407 dispensable SGs, respectively. Distinct from the NLR composition in the *Arabidopsis thaliana* pan-NLRome, no TIR-NLR (TNL) genes were found in the rice genomes. Rice NLRs were categorized into three types: CNL (including CC-NB-LRR or CC-NB), NL (including NB-LRR or NBS) and null (NLR genes identified by syntelogs whose encoding proteins contain no canonical NBS domain). The sizes of NLRs in cultivated and weedy genomes were both significantly larger than those in the wild group (*P*-value = 2.8e-5 and 4.3e-5, Student’s *t* test) (Additional file 1: Fig. S11a), which could be the consequence of disease-resistance gene aggregation during domestication and improvement. However, the relatively low assembly quality of the wild genomes may also be related to this difference, considering that the completeness of assemblies was significantly correlated with the NLR size (Pearson’s correlation = 0.53, *P*-value = 9.7e-7) (Additional file 1: Fig. S11b). At the subspecies level, although NLR gene numbers were similar between XI and GJ, the XI genomes contained more NLs than those in GJ (*P* = 4.3e-10, Student’s *t* test), and GJ had more CNLs (*P*-value = 3.6e-6, Student’s *t* test) (Additional file 1: Fig. S11c). Null NLRs without canonical NBS domains were more abundant in GJ than in XI (*P*-value = 1.6e-10, Student’s *t* test), implying that more NLRs in GJ may degenerate functionally by losing domains.

Adopting the definition by Wang et al. (2019) of an NLR cluster containing more than two NLR genes distributed within a 300-kb genomic region, 43.2%-59.9% of NLR genes in rice were located in such clusters, where GJ showed more clusters in both numbers and proportions across all NLRs than XI (Additional file 1: Fig. S11d). Head-to-head pairing of NLR genes is highly associated with disease resistance in plants, with one NLR acting in effector recognition (known as a sensor) and the other acting in signaling activation (known as a helper). We found 28 to 54 such paired NLRs per genome. A total of 6,008 NLRs encoded at least one non-canonical NLR domain (NBS, LRR and CC), also known as the integrated domain (ID), representing 116 distinct Pfam domains and 16.2% of the total NLRs, which was much higher than that in *Arabidopsis thaliana* (5.0%). We identified 480 distinct architectures in the NLRome, of which only 67 were found in the Nipponbare reference genome (IRGSP v1.0). Fewer than 3% of architectures, 12, correspond only to different configurations of the canonical CC, NBS and LRR domains, even though they accounted for the majority (83.8%) of NLRs. Although the NLR syntelogs among individuals were well defined by genome synteny, within a single SG, the functional types (CNL, NL or null) and structural types (e.g., in clusters or pairs) were diversified, particularly for the core NLR SGs (Additional file 1: Fig. S12).

**Reference**

McKenna, A., Hanna, M., Banks, E., Sivachenko, A., Cibulskis, K., Kernytsky, A., Garimella, K., Altshuler, D., Gabriel, S., and Daly, M., et al. (2010). The genome analysis toolkit: a MapReduce framework for analyzing next-generation DNA sequencing data. Genome Res 20:1297-1303.

Cingolani, P., Platts, A., Wang, L.L., Coon, M., Nguyen, T., Wang, L., Land, S.J., Lu, X., and Ruden, D.M. (2014). A program for annotating and predicting the effects of single nucleotide polymorphisms, snpeff. Fly 6:80-92.

Ma, X., Fan, J., Wu, Y., Zhao, S., Zheng, X., Sun, C., and Tan, L. (2020). Whole-genome de novo assemblies reveal extensive structural variations and dynamic organelle-to-nucleus DNA transfers in African and Asian rice. The Plant Journal 104:596-612.

Xie, X., Du, H., Tang, H., Tang, J., Tan, X., Liu, W., Li, T., Lin, Z., Liang, C., and Liu, Y. (2021). A chromosome-level genome assembly of the wild rice *Oryza rufipogon* facilitates tracing the origins of Asian cultivated rice. Science China Life Sciences 64:282-293.

Shang, L., Li, X., He, H., Yuan, Q., Song, Y., Wei, Z., Lin, H., Hu, M., Zhao, F., and Zhang, C., et al. (2022). A super pan-genomic landscape of rice. Cell Res 32:878-896.

Qin, P., Lu, H., Du, H., Wang, H., Chen, W., Chen, Z., He, Q., Ou, S., Zhang, H., and Li, X., et al. (2021). Pan-genome analysis of 33 genetically diverse rice accessions reveals hidden genomic variations. Cell 184:3542-3558.

Steuernagel, B., Jupe, F., Witek, K., Jones, J.D., and Wulff, B.B. (2015). NLR-parser: rapid annotation of plant NLR complements. Bioinformatics 31(10):1665-1667.

Li, P., Quan, X., Jia, G., Xiao, J., Cloutier, S., and You, F.M. (2016). RGAugury: a pipeline for genome-wide prediction of resistance gene analogs (RGAs) in plants. BMC Genomics 17(1):852.

Wang, L., Zhao, L., Zhang, X., Zhang, Q., Jia, Y., Wang, G., Li, S., Tian, D., Li, W., and Yang, S. (2019). Large-scale identification and functional analysis of NLR genes in blast resistance in the Tetep rice genome sequence. Proc Natl Acad Sci U S A 116:18479-18487.
