# Supplementary material for: A syntelog-based pan-genome provides insights into rice domestication and de-domestication
Source: Genome Biol. 2023 Aug 3;24:179. doi: 10.1186/s13059-023-03017-5 (PMC10401782; doi:10.1186/s13059-023-03017-5)
Supplement: Supplementary file 4 — Additional file 4. Review history. [file 13059_2023_3017_MOESM4_ESM.docx]

Review History

**First round of review**

**Reviewer 1**

**Were you able to assess all statistics in the manuscript, including the appropriateness of statistical tests used?**

Yes, and I have assessed the statistics in my report.

**Comments to author:**

In this manuscript Wu and colleagues produce an insightful view of rice evolution buy considering the pan-genome, and so can get a better grip on the introgression history involved. This is definitely the right direction. While many of the findings are 'rediscovering' previous findings (such as the japonica origin of most domestication syndrome genes), it is nevertheless useful to see from a different perspective, and the origins of weedy forms is particularly useful.

This is the second time I have reviewed this manuscript, and in the first round of reviews for a different journal I had a number of comments concerning the methodology which were well addressed. I therefore was comfortable at that point the manuscript should be accepted, and make the same recommendation to this journal.

**Reviewer 2**

**Were you able to assess all statistics in the manuscript, including the appropriateness of statistical tests used?**

Yes, and I have assessed the statistics in my report.

**Comments to author:**

I reviewed this manuscript in the previous submission in Nature Plants. The revised manuscript has adequately addressed my previous questions. I do not have further questions.
